# Food Sources of Total Energy and Nutrients among U.S. Infants and Toddlers: National Health and Nutrition Examination Survey 2005–2012

**DOI:** 10.3390/nu7085310

**Published:** 2015-08-14

**Authors:** Carley A. Grimes, Ewa A. Szymlek-Gay, Karen J. Campbell, Theresa A. Nicklas

**Affiliations:** 1 Centre for Physical Activity and Nutrition Research, School of Exercise and Nutrition Sciences, Deakin University, 221 Burwood Highway, Melbourne, VIC 3125, Australia; E-Mail: carley.grimes@deakin.edu.au; 2 Centre for Physical Activity and Nutrition Research, School of Exercise and Nutrition Sciences, Deakin University, 75 Pigdons Road, Geelong, VIC 3216, Australia; E-Mail: karen.campbell@deakin.edu.au; 3 Children’s Nutrition Research Center, Baylor College of Medicine, 1100 Bates Ave, Houston, TX 77030, USA; E-Mail: tnicklas@bcm.edu

**Keywords:** National Health and Nutrition Examination Survey (NHANES), infants, toddlers, nutrients, energy, food sources

## Abstract

Understanding the dietary intakes of infants and toddlers is important because early life nutrition influences future health outcomes. The aim of this study was to determine the dietary sources of total energy and 16 nutrients in a nationally representative sample of U.S. infants and toddlers aged 0–24 months. Data from the 2005–2012 National Health and Nutrition Examination Survey were analyzed. Dietary intake was assessed in 2740 subjects using one 24-h dietary recall. The population proportion was used to determine the contribution of foods and beverages to nutrient intakes. Overall infant formulas and baby foods were the leading sources of total energy and nutrients in infants aged 0–11.9 months. In toddlers, the diversity of food groups contributing to nutrient intakes was much greater. Important sources of total energy included milk, 100% juice and grain based mixed dishes. A number of foods of low nutritional quality also contributed to energy intakes including sweet bakery products, sugar-sweetened beverages and savory snacks. Overall non-flavored milks and ready-to-eat cereals were the most important contributors to micronutrient intakes. In conclusion this information can be used to guide parents regarding appropriate food selection as well as inform targeted dietary strategies within public health initiatives to improve the diets of infants and toddlers.

## 1. Introduction

Good nutrition during infancy and early childhood is important. During this period children have high nutrient needs and the consumption of nutrient rich foods is essential for adequate growth and development [1]. Feeding practices such as breast feeding and the timing of introduction of complimentary foods can influence the risk of overweight and obesity in childhood [2] and poor dietary intakes may trigger metabolic programming pathways which predispose children to chronic diseases in later life [3]. Furthermore, exposure to different foods in early life shapes the development of food preferences [4] and eating behaviors have been shown to track across the life course [5,6]. Together these factors emphasize the importance of focusing on nutrition practices during early life to protect future health.

Data from the cross-sectional Feeding Infants and Toddlers Study (FITS), conducted in 2002 (*n* = 3022, 0–24 months) and replicated in 2008 (*n* = 3273, 0–47 months), indicated that although the diets of U.S. infants and toddlers were overall nutritionally adequate some concerns regarding the quality of children’s diets were identified [7]. These related to low intakes of total fat, vitamin E, potassium, fiber, iron and zinc and excessive intakes of energy, saturated fat, sodium, folate, vitamin A and zinc. It should be noted that in some children zinc intake fell below dietary recommendations, yet in others zinc intake was high due to the contribution of zinc from dietary supplements, likewise supplements contributed to the high intakes of folate and vitamin A [7]. Other analyses based on data from the 2003–2010 National Health and Nutrition Examination Survey (NHANES) also indicate high intakes of sodium and low intakes of potassium in U.S. infants and toddlers [8]. More broadly in the general U.S. population aged 2 years and over nutrients that are considered to be of public health concern due to their relationship with adverse health outcomes, include calcium, vitamin D, iron, fiber, potassium, sodium and saturated fat. Of these nutrients calcium, vitamin D, iron, fiber and potassium are under consumed, whilst sodium and saturated fat are overconsumed [9]. Other identified shortfall nutrients in the diets of Americans aged 2 years and above include vitamin C and magnesium [9]. To help improve the diets of infants and toddlers it is important to understand the food sources of these nutrients.

Previously, Fox *et al.* used data from the 2002 FITS to report on the contribution of foods and supplements to energy and 24 selected nutrients in children aged 4–24 months [10]. More recently Maalouf *et al.* reported on the food sources of dietary sodium only in U.S. infants and toddlers using data from the U.S. 2003–2010 NHANES [11]. At present, comprehensive and recent national data identifying the food sources of nutrients which are identified as areas of concern in the diets of infants and toddlers are not available. An update on this information would be timely to help inform the development of the U.S. 2020 Dietary Guidelines for Infants and Toddlers [12]. Hence the primary aim of this study was to determine the contribution of foods and beverages to intakes of total energy and 16 selected nutrients in a nationally representative sample of U.S. infants and toddlers aged 0–24 months.

## 2. Methods

### 2.1. Study Design and Participants

Data come from the U.S. 2005–2012 National Health and Nutrition Examination Survey (NHANES). The NHANES is a cross-sectional survey which utilizes a complex, multistage, probability sampling procedure to provide nationally representative estimates on the health and nutritional status of the non-institutionalized U.S. civilian population. Full details of the sampling framework and methodology can be found elsewhere [13]. The NHANES was approved by the National Center for Health Statistics (NCHS) ethics review board [14]. This analysis includes participants aged from birth to 23.9 months. To obtain an adequate sample size to produce reliable estimates within this age group data from four survey cycles (2005–2006, 2007–2008, 2009–2010, and 2011–2012) were combined. Written parental/guardian consent was obtained for all participants.

### 2.2. Dietary Intake

One face-to-face 24-h dietary recall was completed on the infant or toddler using the parent as a proxy to determine dietary intake. The dietary recall was administered by trained interviewers using the United States Department of Agriculture (USDA) Automated Multiple–Pass Method [15]. Two 24-h dietary recalls are routinely collected in the NHANES, however we have included data only from the 1st 24-h dietary recall as this is an appropriate method to estimate population mean food group contributions to nutrient intake [16]. Of the 2857 children aged from birth to 23.9 months who agreed to participate, 2791 (98%) completed the 1st 24-h dietary recall. Participants with dietary recall data that was deemed unreliable by the NCHS were excluded (*n* = 51). This left an analytical sample of 2740, including 765 infants aged 0–5.9 months, 854 infants aged 6–11.9 months and 1121 toddlers aged 12–23.9 months. Food and beverages consumed were converted to nutrient intakes using the USDA’s Food and Nutrient Database for Dietary Studies (FNDDS) [17]. The FNDDS uses food composition data from the USDA National Nutrient Database for Standard Reference (NNDSR) [18]. Each NHANES survey cycle has its own corresponding FNDDS and NNDSR. Of note, within NHANES vitamin D has only been assessed since 2007–2008. To determine the vitamin D content of foods consumed in the earlier cycle 2005–2006 values from the vitamin D addendum to FNDDS3 food composition database were inputted [19].

During the 24-h dietary recall, the child proxy reported the number of occasions the child consumed human milk. No data on the amount of human milk consumed or timing of the feeding occasion was recorded. Five hundred and sixty one participants (20% weighted) consumed human milk on the day of the dietary recall. To determine the amount of human milk consumed and corresponding nutrient intake provided we utilized the methodology previously described in the Feeding Infants and Toddler Study [20] and a previous NHANES assessment [8]. Participants who were exclusively breastfed were allocated a standard reference value of 780 mL/day of human milk if aged 0–5.9 months and 600 mL/day of human milk if aged 6.0–11.9 months. If the participant was partially breastfed the amount of human milk allocated was 780 mL/day minus the total amount of “other milks (mL/day)” consumed on the day of the recall if aged 0–5.9 months; or 600 mL/day minus the total amount of “other milks (mL/day)” consumed if aged 6–11.9 months. “Other milks” included infant formula, cow’s/goat’s milk, flavored milk or soy/rice milk. If the total daily volume of “other milks” exceeded the age specific daily reference value (*i.e.*, 780 mL or 600 mL), the subject was allocated 89 mL of human milk per reported feeding occasion. In children aged 12–17.9 months and 18–23.9 months, the total daily amount of human milk was calculated as 89 mL or 59 mL, respectively, for every reported feeding occasion. The nutrient content of human milk was obtained from the USDA NNDSR 26 [18].

### 2.3. Food Group Classification

The What We Eat in America (WWEIA) food category classification system [18] was used to calculate the contribution of energy and nutrients from each food category. The classification scheme includes 150 individual food categories, which are organized within major food categories (*n* = 15 e.g., “milk and dairy”, “protein foods”) and sub-major food categories (*n* = 47 e.g., “milk”, “flavored milk”, “dairy drinks and substitutes”, “cheese” and “yogurt”) [18]. FNDDS contains approximately 7200 unique USDA food codes and under the WWEIA food category classification system each food code is assigned to one of the 150 WWEIA food categories. A copy of the WWEIA food group category classification system can be found at the United States Department of Agriculture website [18]. Of note, as per this classification system “100% juice” includes 100% fruit and vegetable juices and “milk” includes non-flavored whole, reduced fat, low fat and non-fat milk varieties. All flavored milks fall under the category “flavored milk”. Under the WWEIA food category classification system “Baby beverages” is a major food category which includes two sub-major food categories: “Baby juice” and “Baby water”. These sub-major food categories include juice and water products that are specifically marketed as baby beverage products within the US food supply.

### 2.4. Other Measures

Demographic characteristics of the study child, including age and sex, were collected from the infant or toddler proxy via questionnaires. Weight and recumbent length were measured at the Mobile Examination Centre (MEC) interview and BMI-for-age *z*-scores were calculated using the World Health Organization Child Growth Standards [21]. The child proxy self-reported the study child’s race or ethnic group according to pre-defined categories used in the NHANES. In this analysis race/ethnic categories reported included Non-Hispanic White, Non-Hispanic Black, Mexican American, and due to the small sample size in the groups which included Other Hispanic, Other Race—including Multi-Racial and Non-Hispanic Asian, these three categories were combined. To describe the socioeconomic background of participants the highest level of education attained by the Head of Household (defined as the person who rents or owns the household residence and is aged 18 years or over) was used. Education status was grouped into one of three categories: low: included those with some or no high school education (<9th grade and 9–11th grade), medium: included those with high school/grad/General Educational Development equivalent, or high: included those with some college or Associate’s Degree, College Graduate or above.

### 2.5. Statistical Analysis

Statistical analyses were completed using STATA/SE 13.1 software (StataCorp, College Station, TX, USA). The complex survey design of the NHANES (*i.e.*, clustering and stratification) was accounted for in all analyses. To produce nationally representative estimates and account for non-response and day of the week for the dietary recall we used a combined 8-year dietary day one sample weight [22]. Descriptive statistics (mean and standard errors or *n* and % weighting) are reported for demographic characteristics, nutrient intakes and the proportion of participants who were exclusively breastfed, partially breastfed or not breastfed on the day of the 24-h dietary recall. Dietary sources of total energy, macronutrients (protein, total fat, saturated fat, total carbohydrate and total sugars), fiber and selected micronutrients (vitamin A, folate, vitamin C, vitamin D, vitamin E, potassium, calcium, magnesium, iron and zinc) are reported. Although sodium is identified as a nutrient of concern in this age group, food sources of sodium in U.S. infant and toddlers using recent NHANES data were previously reported [11] and hence not replicated here. The population proportion formula [23] was used to determine the contribution of each nutrient from each WWEIA sub-major (*n* = 47) food group:

Percentage contribution of food group to nutrient intake = [*sum of nutrient intake from food group ÷ total sum of nutrient from all foods*] × 100

Only food groups that contributed ≥1% to daily nutrient intakes are reported and are ranked in order across each age group. Food group contribution calculations were performed separately for each age group, 0–5.9 months, 6–11.9 months and 12–23.9 months. Within supplemental Tables S1–S17 the contribution of nutrients from the WWEIA individual (*n* = 150) food groups are reported.

## 3. Results

Table 1 shows participant demographic characteristics and mean nutrient intakes by age group. Of the 2740 participants, 50.4% were boys and just over half were Non-Hispanic White (54.1%) and had a high socioeconomic background (55.8%). On the day of the 24-h dietary recall just under half (41.5%) of infants aged 0–5.9 months were either exclusively (*i.e.*, received no other foods/beverages during the 24-h period except for breast milk) or partially breastfed. Only 24.3% of infants aged 6.0–11.9 months and 7.5% of toddlers received some breast milk and in toddlers the majority (92.5%) received no breast milk (Table 1). Table 2, Table 3, Table 4, Table 5, Table 6, Table 7, Table 8, Table 9, Table 10, Table 11, Table 12, Table 13, Table 14, Table 15, Table 16, Table 17 and Table 18 show the dietary sources of total energy and 16 nutrients from WWEIA sub-major food groups. The contribution of total energy and nutrients from WWEIA minor food groups are provided in the supplemental Tables S1–S17.

### Total Energy, Macronutrients and Dietary Fiber

In the first year of life the top three sources of total energy were infant formulas, human milk and baby foods (Table 2). Cumulatively these contributed to 99.3% and 73.5% of total energy intake among infants 0–5.9 months and 6.0–11.9 months, respectively. Other sources of total energy contributing at least 2% among 6.0–11.9 months were milk, fruits and mixed dishes-grain based. Among toddlers the contribution of total energy intake from milk-based products was much lower than for infants, however milk was still the most important source of total energy intake (22.4%). Within this age group the number and diversity of food groups contributing to total energy intake was far greater. Food groups which contributed to at least 3% of total energy intake included 100% juice, grain based mixed dishes, fruits, sweet bakery products, breads, rolls and tortillas, poultry and sugar-sweetened beverages.

**Table 1:** Demographic characteristics and nutrient intake in U.S. infants and toddlers aged 0–23.9 months by age group: NHANES 2005–2012 (*n* = 2740).

Characteristic	Age Group		
	0–5.9 months	6–11.9 months	12–23.9 months
Participants ^a^	765 (23.3%)	854 (27.0%)	1121 (49.7%)
Sex ^a^			
Boy	407 (53.0%)	422 (46.7%)	570 (51.2%)
Girl	358 (47.0%)	432 (53.3%)	551 (48.8%)
Age (months) ^b^	2.8 ± 0.06	8.6 ± 0.08	17.4 ± 0.13
BMI-for-age ^b,c^	0.31 ± 0.04	0.46 ± 0.06	0.63 ± 0.05
Race/ethnic group ^a^			
Non-Hispanic White	238 (52.9%)	276 (56.8%)	333 (53.3%)
Non-Hispanic Black	134 (14.5%)	135 (12.1%)	236 (13.5%)
Mexican American	271 (18.6%)	297 (17.4%)	345 (17.7%)
Other Hispanic, Other Race and Multi-Racial	122 (14.0%)	146 (13.7%)	207 (15.6%)
Head of Household Education Status ^a,d^			
Low (less than high school)	242 (23.9%)	256 (21.1%)	326 (19.9%)
Mid (high school)	167 (20.6%)	226 (24.3%)	276 (23.4%)
High (more than high school)	332 (55.5%)	342 (54.6%)	487 (56.7%)
Breastfeeding rates ^a,e^			
Exclusively breastfed	122 (21.4%)	4 (0.8%)	0 (0%)
Partially breastfed	182 (20.1%)	177 (23.5%)	76 (7.5%)
Not breastfed	461 (58.4%)	673 (75.7%)	1045 (92.5%)
Nutrient intake			
Energy (kcal/day) ^b^	612.5 ± 6.4	847.3 ± 13.3	1217.2 ± 20.5
Protein (g/day) ^b^	12.1 ± 0.2	21.3 ± 0.5	46.3 ± 0.8
Total fat (g/day) ^b^	33.3 ± 0.3	35.4 ± 0.6	45.6 ± 0.7
Saturated fat (g/day) ^b^	14.0 ± 0.2	14.5 ± 0.3	18.6 ± 0.3
Total carbohydrate (g/day) ^b^	66.8 ± 0.8	112.9 ± 1.9	159.1 ± 3.2
Total sugars (g/day) ^b^	61.4 ± 0.7	77.4 ± 1.1	92.2 ± 1.8
Fiber (g/day) ^b^	0.4 ± 0.0	5.0 ± 0.2	8.2 ± 0.2
Vitamin A (ug/day) ^b^	537.8 ± 7.4	675.4 ± 12.4	562.9 ± 14.5
Folate (ug/day) ^b^	78.6 ± 1.7	136.2 ± 4.0	218.6 ± 5.8
Vitamin C (mg/day) ^b^	60.1 ± 1.2	94.1 ± 2.4	79.4 ± 3.3
Vitamin D (ug/day) ^b^	6.4 ± 0.2	7.1 ± 0.2	8.2 ± 0.2
Vitamin E (mg/day) ^b^	5.3 ± 0.2	6.9 ± 0.2	3.7 ± 0.1
Potassium (mg/day) ^b^	616.8 ± 9.4	1129.3 ± 22.5	1866.9 ± 31.0
Calcium (mg/day) ^b^	469.7 ± 9.6	649.0 ± 12.4	1011.7 ± 21.0
Magnesium (mg/day)	52.7 ± 1.2	108.9 ± 2.5	172.1 ± 3.3
Iron (mg/day) ^b^	9.2 ± 0.3	15.9 ± 15.9	9.4 ± 0.3
Zinc (mg/day) ^b^	4.2 ± 0.1	6.1 ± 0.2	6.9 ± 0.1

^a^ *n* (% Weighted); ^b^ mean ± SEM; ^c^ Includes data from 2715 participants; ^d^ Includes data from 2654 participants: 57 participants had missing data, 1 refused to answer the question and 28 responded “don’t know”; ^e^ Represents breastfeeding rates on the day of the 24-h dietary recall.

Among infants 0–11.9 months the major source of all macronutrients (protein, total fat, saturated fat, total carbohydrate and total sugars) was infant formulas (Table 3, Table 4, Table 5, Table 6, Table 7 and Table 8). For 0–5.9 months this was followed by either human milk or baby foods and together these food groups accounted for over 98% of macronutrient intakes. For 6.0–11.9 months human milk and baby foods were still important sources of each macronutrient but generally to a lesser degree as the number of other food groups contributing to intakes increased. At this age, milk became another important source of the different macronutrients. In addition grain based mixed dishes and sweet bakery products consistently contributed more than 1% of intake for each macronutrient. Cheese, eggs and meat/poultry based products each contributed more than 1% of intake for protein, total fat and saturated fat. With regards to total carbohydrate and total sugars, fruits, baby beverages and 100% juice were other important food groups contributing roughly 3%–4% of intakes.

As expected, compared to infants, in toddlers a greater number of food groups contributed to more than 1% of macronutrient intakes (Table 2, Table 3, Table 4, Table 5, Table 6 and Table 7). Across all macronutrients milk was the top source, contributing to 32.7% of protein, 30.1% of total fat, 42.9% of saturated fat, 14.1% of total carbohydrate and 26.3% of total sugars. Other important sources contributing to more than 4% of intakes for protein included poultry (8.5%), grain based mixed dishes (5.7%), cheese (4.9%), and meat, poultry, seafood mixed dishes (4.1%). For total fat intake other important sources were cured meats/poultry (5.2%), poultry (5.2%), grain based mixed dishes (5.0%), cheese (5.0%), sweet bakery products (4.6%) and eggs (4.3%); and for saturated fat intake cheese (7.4%), cured meats/poultry (4.5%) and grain based mixed dishes (4.4%). With regard to total carbohydrate intake 100% juice (11.1%), fruits (9.5%), and sugar-sweetened beverages (5.8%), grain based mixed dishes (5.6%), sweet bakery products (5.4%), and breads, rolls and tortillas (5.2%) were all important food sources. With the exception of grain based mixed dishes and breads, rolls and tortillas, these food groups were also important food sources of total sugars.

The highest ranked sources of dietary fiber at 0–5.9 months were baby foods (87.5%), infant formulas (3.5%) and fruits (2.6%) (Table 8). Similarly in 6.0–11.9 month olds baby foods (55.7%) and fruits (11.8%) were important sources, along with vegetables—excluding potatoes (5.7%), plant-based protein foods (4.7%) and grain based mixed dishes (4.2%). Among toddlers the top five sources of fiber included fruits (21.8%), grain based mixed dishes (8.4%), vegetables—excluding potatoes (8.3%), breads, rolls and tortillas (7.3%), and baby foods (6.5%).

The dietary sources of micronutrients (*i.e.*, vitamin A, folate, vitamin C, vitamin D, vitamin E, potassium, calcium, magnesium, iron and zinc) are shown in Table 9, Table 10, Table 11, Table 12, Table 13, Table 14, Table 15, Table 16, Table 17 and Table 18. Among 0–5.9 month old infants, infant formulas were the top food source of all micronutrients ranging from a contribution of 66.7% for vitamin A to 95.8% for vitamin D. The two other important food sources in this age group were human milk and baby foods. The contribution of human milk was greatest for vitamin A, vitamin C, folate, potassium, calcium, magnesium and zinc (all >10%). Whereas human milk contributed substantially less to vitamin D (4.1%), vitamin E (4.0%) and iron intakes (0.9%). Baby foods accounted for about 3%–4% of intakes for vitamin A, vitamin C, folate, vitamin E and zinc, whereas these foods contributed to higher intakes for potassium (6.3%), calcium (7.2%), magnesium (16.6%) and iron (21.4%). The types of baby foods contributing to micronutrient intakes are shown in the supplemental Tables (S8–S17).

**Table 2:** Food sources of total energy among US infants and toddlers aged 0–23.9 months by age group: NHANES 2005–2012 (*n* = 2740) ^a^.

0–5.9 Months				6–11.9 Months				12–23.9 Months			
Rank	Food Category	% of Daily Intake	% Cumulative	Rank	Food Category	% of Daily Intake	% Cumulative	Rank	Food Category	% of Daily Intake	% Cumulative
1	Infant formulas	65.4	65.4	1	Infant formulas	47.1	47.1	1	Milk	22.4	22.4
2	Human milk	30.1	95.5	2	Baby foods	16.5	63.6	2	100% juice	5.9	28.3
3	Baby foods	3.7	99.3	3	Human milk	9.9	73.5	3	Mixed dishes—grain-based	5.5	33.9
				4	Milk	3.1	76.6	4	Fruits	4.8	38.7
				5	Fruits	2.3	79.0	5	Sweet bakery products	4.6	43.2
				6	Mixed dishes—grain-based	2.3	81.3	6	Breads, rolls, tortillas	3.8	47.1
				7	Sweet bakery products	1.8	83.1	7	Poultry	3.6	50.77
				8	Baby beverages	1.5	84.7	8	Sugar-sweetened beverages	3.1	53.8
				9	100% juice	1.5	86.2	9	Baby foods	2.6	56.4
				10	Breads, rolls, tortillas	1.1	87.3	10	Cheese	2.6	59.0
				11	Mixed dishes—Soups	1.0	88.3	11	Cured Meats/Poultry	2.5	61.5
								12	Crackers	2.4	64.0
								13	Savory snacks	2.4	66.3
								14	Ready-to-eat cereals	2.3	68.6
								15	Eggs	2.2	70.8
								16	Infant formulas	2.1	72.9
								17	Mixed dishes—meat, poultry, seafood	2.0	74.9
								18	White potatoes	2.0	76.9
								19	Yoghurt	1.7	78.6
								20	Quick breads & bread products	1.6	80.2
								21	Plant-based protein foods	1.6	81.8
								22	Mixed dishes—Soups	1.5	83.3
								23	Mixed dishes—Pizza	1.5	84.8
								24	Cooked cereals	1.4	86.2
								25	Flavored milk	1.3	87.5
								26	Human milk	1.3	88.8
								27	Candy	1.3	90.1
								28	Other desserts	1.2	91.3
								29	Vegetables, excluding potatoes	1.2	92.5

^a^ only WWEIA sub-major food categories that contributed ≥1% to daily intake are reported.

**Table 3:** Food sources of protein among US infants and toddlers aged 0–23.9 months by age group: NHANES 2005–2012 (*n* = 2740).

0–5.9 Months				6–11.9 Months				12–23.9 Months			
Rank	Food Category	% of Daily Intake	% Cumulative	Rank	Food Category	% of Daily Intake	% Cumulative	Rank	Food Category	% of Daily Intake	% Cumulative
1	Infant formulas	72.9	72.9	1	Infant formulas	41.6	41.6	1	Milk	32.7	32.7
2	Human milk	22.4	95.3	2	Baby foods	16.1	57.7	2	Poultry	8.5	41.2
3	Baby foods	4.4	99.7	3	Milk	7.0	64.7	3	Mixed dishes—grain-based	5.7	46.9
				4	Human milk	5.8	70.5	4	Cheese	4.9	51.8
				5	Mixed dishes—grain-based	3.6	74.1	5	Mixed dishes—meat, poultry, seafood	4.1	55.9
				6	Poultry	2.4	76.5	6	Cured meats/poultry	3.9	59.8
				7	Mixed dishes—Soups	2.1	78.7	7	Eggs	3.9	63.7
				8	Cheese	2.0	80.7	8	Breads, rolls, tortillas	3.5	67.2
				9	Mixed dishes—meat, poultry, seafood	2.0	82.6	9	Meats	2.3	69.6
				10	Eggs	1.7	84.3	10	Mixed dishes—Soups	2.1	71.7
				11	Cured meats/poultry	1.7	86.0	11	Yoghurt	1.9	73.6
				12	Yoghurt	1.7	87.7	12	Baby foods	1.9	75.5
				13	Plant-based protein foods	1.5	89.1	13	Plant-based protein foods	1.9	77.5
				14	Breads, rolls, tortillas	1.4	90.6	14	Mixed dishes—Pizza	1.7	79.2
				15	Sweet bakery products	1.1	91.7	15	Sweet bakery products	1.6	80.8
				16	Meats	1.0	92.7	16	Fruits	1.3	82.1
								17	Flavored milk	1.3	83.4
								18	Ready-to-eat cereals	1.3	84.7
								19	Infant formulas	1.3	86.0
								20	Cooked cereals	1.2	87.2
								21	Vegetables, excluding potatoes	1.1	88.4
								22	Crackers	1.1	89.5
								23	Mixed dishes—Sandwiches (single code)	1.1	90.6
								24	Quick breads & bread products	1.0	91.6
								25	Dairy drinks and substitutes	1.0	92.6
								26	Savory snacks	1.0	93.6

^a^ only WWEIA sub-major food categories that contributed ≥1% to daily intake are reported.

**Table 4:** Food sources of total fat among US infants and toddlers aged 0–23.9 months by age group: NHANES 2005–2012 (*n* = 2740).

0–5.9 Months				6–11.9 Months				12–23.9 Months			
Rank	Food Category	% of Daily Intake	% Cumulative	Rank	Food Category	% of Daily Intake	% Cumulative	Rank	Food Category	% of Daily Intake	% Cumulative
1	Infant formulas	64.4	64.4	1	Infant formulas	60.4	60.4	1	Milk	30.1	30.1
2	Human milk	34.7	99.1	2	Human milk	14.8	75.2	2	Cured Meats/Poultry	5.2	35.3
				3	Baby foods	5.0	80.2	3	Poultry	5.2	40.5
				4	Milk	3.8	84.0	4	Mixed dishes—grain-based	5.0	45.5
				5	Mixed dishes—grain-based	1.9	85.9	5	Cheese	5.0	50.5
				6	Cured meats/poultry	1.6	87.5	6	Sweet bakery products	4.6	55.1
				7	Sweet bakery products	1.5	89.0	7	Eggs	4.3	59.44
				8	Cheese	1.3	90.3	8	Savory snacks	3.1	62.5
				9	Eggs	1.1	91.4	9	Infant formulas	2.9	65.4
								10	Crackers	2.9	68.3
								11	Plant-based protein foods	2.4	70.7
								12	White potatoes	2.4	73.1
								13	Mixed dishes—meat, poultry, seafood	2.2	75.3
								14	Human milk	2.2	77.5
								15	Fats and oils	2.0	79.5
								16	Mixed dishes—Pizza	1.9	81.3
								17	Breads, rolls, tortillas	1.7	83.1
								18	Quick breads and bread products	1.5	84.6
								19	Mixed dishes—Soups	1.5	86.1
								20	Mixed dishes—Sandwiches (single code)	1.2	87.3
								21	Baby foods	1.2	88.5
								22	Meats	1.2	89.6
								23	Other desserts	1.1	90.7
								24	Flavored milk	1.1	91.8
								25	Mixed dishes—Mexican	1.0	92.8

^a^ only WWEIA sub-major food categories that contributed ≥1% to daily intake are reported.

**Table 5:** Food sources of saturated fat among US infants and toddlers aged 0–23.9 months by age group: NHANES 2005–2012 (*n* = 2740).

0–5.9 Months				6–11.9 Months				12–23.9 Months			
Rank	Food Category	% of Daily Intake	% Cumulative	Rank	Food Category	% of Daily Intake	% Cumulative	Rank	Food Category	% of Daily Intake	% Cumulative
1	Infant formulas	61.8	61.8	1	Infant formulas	61.8	61.8	1	Milk	42.9	42.9
2	Human milk	37.7	99.5	2	Human milk	16.6	78.3	2	Cheese	7.4	50.2
				3	Milk	5.4	83.7	3	Cured Meats/Poultry	4.5	54.8
				4	Baby foods	3.1	86.8	4	Mixed dishes—grain-based	4.4	59.2
				5	Cheese	1.9	88.7	5	Eggs	3.5	62.7
				6	Mixed dishes—grain-based	1.7	90.4	6	Sweet bakery products	3.1	65.8
				7	Cured Meats/Poultry	1.4	91.8	7	Infant formulas	2.8	68.7
				8	Sweet bakery products	1.0	92.8	8	Poultry	2.7	71.3
				9	Eggs	1.0	93.8	9	Human milk	2.5	73.8
								10	Fats and oils	1.9	75.7
								11	Mixed dishes—Pizza	1.8	77.5
								12	Mixed dishes—meat, poultry, seafood	1.7	79.2
								13	Flavored milk	1.6	80.8
								14	Crackers	1.5	82.3
								15	White potatoes	1.5	83.8
								16	Other desserts	1.5	85.2
								17	Yoghurt	1.4	86.6
								18	Savory snacks	1.3	87.9
								19	Plant-based protein foods	1.1	89.0
								20	Meats	1.1	90.1
								21	Mixed dishes—Soups	1.1	91.1
								22	Mixed dishes—Sandwiches (single code)	1.0	92.2

^a^ only WWEIA sub-major food categories that contributed ≥1% to daily intake are reported.

**Table 6:** Food sources of total carbohydrate among US infants and toddlers aged 0–23.9 months by age group: NHANES 2005–2012 (*n* = 2740).

0–5.9 Months				6–11.9 Months				12–23.9 Months			
Rank	Food Category	% of Daily Intake	% Cumulative	Rank	Food Category	% of Daily Intake	% Cumulative	Rank	Food Category	% of Daily Intake	% Cumulative
1	Infant formulas	64.4	64.4	1	Infant formulas	38.0	38.0	1	Milk	14.1	14.1
2	Human milk	27.2	91.6	2	Baby foods	25.0	63.0	2	100% juice	11.1	25.2
3	Baby foods	6.9	98.4	3	Human milk	7.3	70.2	3	Fruits	9.5	34.7
				4	Fruits	4.5	74.8	4	Sugar-sweetened beverages	5.8	40.5
				5	Baby beverages	2.8	77.6	5	Mixed dishes—grain-based	5.6	46.1
				6	100% juice	2.8	80.4	6	Sweet bakery products	5.4	51.5
				7	Mixed dishes—grain-based	2.3	82.8	7	Breads, rolls, tortillas	5.2	56.7
				8	Sweet bakery products	2.2	85.0	8	Ready-to-eat cereals	3.8	60.5
				9	Milk	1.9	86.9	9	Baby foods	3.8	64.3
				10	Breads, rolls, tortillas	1.5	88.4	10	Crackers	2.5	66.8
				11	Ready-to-eat cereals	1.1	89.5	11	Savory snacks	2.3	69.1
				12	Yoghurt	1.1	90.6	12	Yoghurt	2.2	71.3
								13	White potatoes	2.1	73.4
								14	Candy	2.0	75.4
								15	Quick breads & bread products	1.9	77.3
								16	Cooked cereals	1.9	79.1
								17	Infant formulas	1.7	80.8
								18	Vegetables, excluding potatoes	1.6	82.4
								19	Other desserts	1.5	83.9
								20	Flavored milk	1.4	85.4
								21	Cooked grains	1.3	86.7
								22	Mixed dishes—Soups	1.3	88.0
								23	Mixed dishes—meat, poultry, seafood	1.2	89.3
								24	Mixed dishes—Pizza	1.1	90.4
								25	Plant-based protein foods	1.0	91.4
								26	Sugars	1.0	92.4
								27	Poultry	1.0	93.5
								28	Human milk	1.0	94.4

^a^ only WWEIA sub-major food categories that contributed ≥1% to daily intake are reported.

**Table 7:** Food sources of total sugars among US infants and toddlers aged 0–23.9 months by age group: NHANES 2005–2012 (*n* = 2740).

0–5.9 Months				6–11.9 Months				12–23.9 Months			
Rank	Food Category	% of Daily Intake	% Cumulative	Rank	Food Category	% of Daily Intake	% Cumulative	Rank	Food Category	% of Daily Intake	% Cumulative
1	Infant formulas	67.7	67.7	1	Infant formulas	53.1	53.1	1	Milk	26.3	26.3
2	Human milk	29.6	97.3	2	Baby foods	13.3	66.4	2	100% juice	16.6	42.9
3	Baby foods	1.5	98.7	3	Human milk	10.6	77.0	3	Fruits	11.8	54.7
				4	Fruits	4.5	81.5	4	Sugar-sweetened beverages	9.0	63.7
				5	100% juice	3.6	85.2	5	Sweet bakery products	4.3	68.0
				6	Baby beverages	3.6	88.7	6	Yoghurt	3.5	71.5
				7	Milk	3.1	91.8	7	Infant formulas	2.9	74.4
				8	Yoghurt	1.6	93.4	8	Candy	2.4	76.8
				9	Sweet bakery products	1.3	94.7	9	Flavored milk	2.3	79.1
				10	Sugar-sweetened beverages	1.0	95.7	10	Baby foods	2.2	81.2
								11	Other desserts	2.1	83.4
								12	Ready-to-eat cereals	1.9	85.2
								13	Human milk	1.7	86.9
								14	Mixed dishes—grain-based	1.4	88.4
								15	Sugars	1.4	89.7
								16	Baby beverages	1.2	90.9

^a^ only WWEIA sub-major food categories that contributed ≥1% to daily intake are reported.

**Table 8:** Food sources of fiber among US infants and toddlers aged 0–23.9 months by age group: NHANES 2005–2012 (*n* = 2740).

0–5.9 Months				6–11.9 Months				12–23.9 Months			
Rank	Food Category	% of Daily Intake	% Cumulative	Rank	Food Category	% of Daily Intake	% Cumulative	Rank	Food Category	% of Daily Intake	% Cumulative
1	Baby foods	87.5	87.5	1	Baby foods	55.7	55.7	1	Fruits	21.8	21.8
2	Infant formulas	3.5	90.9	2	Fruits	11.8	67.5	2	Mixed dishes—grain-based	8.4	30.2
3	Fruits	2.6	93.5	3	Vegetables, excluding potatoes	5.7	73.2	3	Vegetables, excluding potatoes	8.3	38.5
4	Vegetables, excluding potatoes	1.7	95.3	4	Plant-based protein foods	4.7	77.8	4	Breads, rolls, tortillas	7.3	45.8
5	Baby beverages	1.6	96.8	5	Mixed dishes—grain-based	4.2	82.0	5	Baby foods	6.5	52.3
				6	Ready-to-eat cereals	2.7	84.7	6	Ready-to-eat cereals	6.2	58.6
				7	Breads, rolls, tortillas	2.5	87.2	7	Plant-based protein foods	5.4	64.0
				8	Mixed dishes—Soups	1.7	88.9	8	White potatoes	3.6	67.6
				9	White potatoes	1.6	90.6	9	100% juice	3.6	71.2
				10	Sweet bakery products	1.5	92.0	10	Cooked cereals	3.3	74.6
				11	Cooked cereals	1.1	93.1	11	Savory snacks	2.9	77.5
				12	100% juice	1.0	94.1	12	Sweet bakery products	2.8	80.3
								13	Mixed dishes—meat, poultry, seafood	2.3	82.6
								14	Mixed dishes—Soups	2.1	84.8
								15	Crackers	1.9	86.7
								16	Quick breads and bread products	1.8	88.5
								17	Mixed dishes—Pizza	1.5	90.0
								18	Poultry	1.4	91.3
								19	Flavored milk	1.1	92.4
								20	Mixed dishes—Mexican	1.1	93.5
								21	Dairy drinks and substitutes	1.0	94.5

^a^ only WWEIA sub-major food categories that contributed ≥1% to daily intake are reported.

**Table 9:** Food sources of Vitamin A among US infants and toddlers aged 0–23.9 months by age group: NHANES 2005–2012 (*n* = 2740).

0–5.9 Months				6–11.9 Months				12–23.9 Months			
Rank	Food Category	% of Daily Intake	% Cumulative	Rank	Food Category	% of Daily Intake	% Cumulative	Rank	Food Category	% of Daily Intake	% Cumulative
1	Infant formulas	66.7	66.7	1	Infant formulas	53.1	53.1	1	Milk	37.4	37.4
2	Human milk	29.9	96.6	2	Baby foods	21.3	74.3	2	Ready-to-eat cereals	8.1	45.4
3	Baby foods	3.3	99.9	3	Human milk	10.8	85.1	3	Vegetables, excluding potatoes	7.5	53.0
				4	Vegetables, excluding potatoes	3.6	88.7	4	Baby foods	4.7	57.7
				5	Milk	3.0	91.7	5	Infant formulas	4.0	61.7
				6	Ready-to-eat cereals	1.8	93.5	6	Eggs	4.0	65.7
								7	Cooked cereals	3.7	69.5
								8	Cheese	3.4	72.8
								9	Mixed dishes—grain-based	3.1	75.9
								10	Human milk	2.5	78.4
								11	Fruits	2.0	80.4
								12	Flavored milk	1.8	82.1
								13	Mixed dishes—meat, poultry, seafood	1.5	83.7
								14	Dairy drinks and substitutes	1.5	85.2
								15	Fats & oils	1.3	86.5
								16	100% juice	1.3	87.8
								17	Quick breads & bread products	1.3	89.1
								18	Mixed dishes—Soups	1.2	90.3
								19	Sugar-sweetened beverages	1.1	91.4
								20	Snack/meal bars	1.0	92.4
								21	Sweet bakery products	1.0	93.4

^a^ only WWEIA sub-major food categories that contributed ≥1% to daily intake are reported.

**Table 10:** Food sources of folate (includes fortified (folic acid in foods) and folate) among US infants and toddlers aged 0–23.9 months by age group: NHANES 2005–2012 (*n* = 2740).

0–5.9 Months				6–11.9 Months				12–23.9 Months			
Rank	Food Category	% of Daily Intake	% Cumulative	Rank	Food Category	% of Daily Intake	% Cumulative	Rank	Food Category	% of Daily Intake	% Cumulative
1	Infant formulas	79.2	79.2	1	Infant formulas	44.5	44.5	1	Ready-to-eat cereals	21.6	21.6
2	Human milk	16.8	96.0	2	Baby foods	14.9	59.5	2	Milk	10.7	32.4
3	Baby foods	3.4	99.4	3	Ready-to-eat cereals	9.4	68.8	3	Mixed dishes—grain-based	8.1	40.5
				4	Human milk	4.4	73.2	4	Breads, rolls, tortillas	7.3	47.8
				5	Mixed dishes—grain-based	3.9	77.2	5	Fruits	4.4	52.3
				6	Plant-based protein foods	2.4	79.6	6	Vegetables, excluding potatoes	4.1	56.3
				7	Fruits	2.4	82.1	7	Sweet bakery products	3.8	60.1
				8	Breads, rolls, tortillas	2.2	84.3	8	Crackers	3.3	63.4
				9	Vegetables, excluding potatoes	2.0	86.3	9	Plant-based protein foods	2.7	66.2
				10	Sweet bakery products	1.8	88.1	10	100% juice	2.6	68.7
				11	Milk	1.7	89.8	11	Cooked cereals	2.6	71.3
				12	Mixed dishes—Soups	1.4	91.2	12	Baby foods	2.1	73.4
				13	Cooked cereals	1.1	92.2	13	Quick breads and bread products	2.1	75.4
				14	Crackers	1.0	93.2	14	Eggs	2.1	77.5
								15	Savory snacks	2.0	79.5
								16	Infant formulas	2.0	81.5
								17	Mixed dishes—Pizza	2.0	83.5
								18	Cooked grains	2.0	85.5
								19	Mixed dishes—meat, poultry, seafood	2.0	87.5
								20	Mixed dishes—Soups	1.8	89.3
								21	White potatoes	1.0	90.3
								22	Poultry	1.0	91.3
								23	Sugar-sweetened beverages	1.0	92.3

^a^ only WWEIA sub-major food categories that contributed ≥1% to daily intake are reported.

**Table 11:** Food sources of Vitamin C among US infants and toddlers aged 0–23.9 months by age group: NHANES 2005–2012 (*n* = 2740).

0–5.9 Months				6–11.9 Months				12–23.9 Months			
Rank	Food Category	% of Daily Intake	% Cumulative	Rank	Food Category	% of Daily Intake	% Cumulative	Rank	Food Category	% of Daily Intake	% Cumulative
1	Infant formulas	70.1	70.1	1	Infant formulas	44.4	44.4	1	100% juice	41.2	41.2
2	Human milk	21.9	92.1	2	Baby foods	22.5	66.8	2	Fruits	14.1	55.3
3	Baby foods	3.7	95.8	3	Baby beverages	13.6	80.4	3	Sugar-sweetened beverages	12.3	67.6
4	Baby beverages	3.5	99.2	4	Human milk	6.3	86.7	4	Baby beverages	7.1	74.6
				5	100% juice	5.9	92.6	5	Baby foods	4.3	79.0
				6	Fruits	2.9	95.5	6	Vegetables, excluding potatoes	4.2	83.2
				7	Vegetables, excluding potatoes	1.2	96.7	7	Infant formulas	3.3	86.5
				8	Sugar-sweetened beverages	1.0	97.7	8	Ready-to-eat cereals	2.2	88.6
								9	Candy	2.0	90.7
								10	Human milk	1.4	92.1
								11	White potatoes	1.3	93.4
								12	Mixed dishes—grain-based	1.2	94.6
								13	Mixed dishes—meat, poultry, seafood	1.0	95.6

^a^ only WWEIA sub-major food categories that contributed ≥1% to daily intake are reported.

**Table 12:** Food sources of Vitamin D among US infants and toddlers aged 0–23.9 months by age group: NHANES 2005–2012 (*n* = 2740).

0–5.9 Months				6–11.9 Months				12–23.9 Months			
Rank	Food Category	% of Daily Intake	% Cumulative	Rank	Food Category	% of Daily Intake	% Cumulative	Rank	Food Category	% of Daily Intake	% Cumulative
1	Infant formulas	95.8	95.8	1	Infant formulas	85.0	85.0	1	Milk	74.0	74.0
2	Human milk	4.1	100.0	2	Milk	8.3	93.3	2	Infant formulas	4.6	78.6
				3	Human milk	1.7	95.0	3	Eggs	3.0	81.5
								4	Flavored milk	2.9	84.5
								5	Ready-to-eat cereals	2.8	87.2
								6	Dairy drinks and substitutes	2.0	89.3
								7	Yoghurt	1.5	90.8
								8	Cheese	1.3	92.1
								9	Mixed dishes—grain-based	1.3	93.4
								10	100% juice	1.0	94.4
								11	Cured meats/poultry	1.0	95.4

^a^ only WWEIA sub-major food categories that contributed ≥1% to daily intake are reported.

**Table 13:** Food sources of Vitamin E among US infants and toddlers aged 0–23.9 months by age group: NHANES 2005–2012 (*n* = 2740).

0–5.9 Months				6–11.9 Months				12–23.9 Months			
Rank	Food Category	% of Daily Intake	% Cumulative	Rank	Food Category	% of Daily Intake	% Cumulative	Rank	Food Category	% of Daily Intake	% Cumulative
1	Infant formulas	91.0	91.0	1	Infant formulas	71.2	71.2	1	Mixed dishes—grain-based	8.7	8.7
2	Baby foods	4.7	95.7	2	Baby foods	16.7	87.9	2	Infant formulas	8.4	17.1
3	Human milk	4.0	99.7	3	Human milk	1.4	89.3	3	Milk	7.3	24.5
				4	Mixed dishes—grain-based	1.4	90.6	4	Plant-based protein foods	5.9	30.4
				5	Baby beverages	1.1	91.7	5	Baby foods	5.8	36.2
				6	Fruits	1.0	92.7	6	Fruits	5.5	41.7
								7	Savory snacks	5.4	47.0
								8	Eggs	4.5	51.6
								9	Vegetables, excluding potatoes	3.9	55.5
								10	Poultry	3.9	59.4
								11	Crackers	3.5	62.9
								12	Sweet bakery products	3.3	66.2
								13	Ready-to-eat cereals	2.9	69.2
								14	Sugar-sweetened beverages	2.8	71.9
								15	Mixed dishes—meat, poultry, seafood	2.6	74.6
								16	100% juice	2.2	76.8
								17	Fats & oils	2.1	78.9
								18	Mixed dishes—Soups	2.1	81.0
								19	Breads, rolls, tortillas	2.1	83.1
								20	White potatoes	2.0	85.0
								21	Mixed dishes—Pizza	1.6	86.7
								22	Quick breads & bread products	1.3	88.0
								23	Flavored or enhanced water	1.2	89.2

^a^ only WWEIA sub-major food categories that contributed ≥1% to daily intake are reported.

**Table 14:** Food sources of potassium among US infants and toddlers aged 0–23.9 months by age group: NHANES 2005–2012 (*n* = 2740).

0–5.9 Months				6–11.9 Months				12–23.9 Months			
Rank	Food Category	% of Daily Intake	% Cumulative	Rank	Food Category	% of Daily Intake	% Cumulative	Rank	Food Category	% of Daily Intake	% Cumulative
1	Infant formulas	70.2	70.2	1	Infant formulas	38.1	38.1	1	Milk	34.8	34.8
2	Human milk	21.8	92.0	2	Baby foods	23.9	62.0	2	100% juice	9.5	44.3
3	Baby foods	6.3	98.3	3	Milk	5.7	67.7	3	Fruits	9.3	53.6
				4	Human milk	5.4	73.1	4	Mixed dishes—grain-based	3.7	57.3
				5	Fruits	5.2	78.3	5	Vegetables, excluding potatoes	2.9	60.2
				6	100% juice	2.7	81.0	6	White potatoes	2.8	63.1
				7	Baby beverages	2.3	83.4	7	Poultry	2.7	65.7
				8	Vegetables, excluding potatoes	2.1	85.5	8	Baby foods	2.6	68.3
				9	Mixed dishes—grain-based	1.8	87.3	9	Yoghurt	2.1	70.5
				10	Mixed dishes—Soups	1.7	89.0	10	Sugar-sweetened beverages	2.1	72.6
				11	Yoghurt	1.4	90.4	11	Mixed dishes—meat, poultry, seafood	2.1	74.7
				12	White potatoes	1.3	91.7	12	Mixed dishes—Soups	1.9	76.5
				13	Plant-based protein foods	1.3	92.9	13	Cured Meats/Poultry	1.8	78.4
								14	Plant-based protein foods	1.7	80.1
								15	Flavored milk	1.6	81.8
								16	Infant formulas	1.5	83.3
								17	Savory snacks	1.5	84.7
								18	Breads, rolls, tortillas	1.4	86.1
								19	Eggs	1.3	87.4
								20	Ready-to-eat cereals	1.3	88.7
								21	Dairy drinks and substitutes	1.1	89.8
								22	Cooked cereals	1.0	90.8

^a^ only WWEIA sub-major food categories that contributed ≥1% to daily intake are reported.

**Table 15:** Food sources of calcium among US infants and toddlers aged 0–23.9 months by age group: NHANES 2005–2012 (*n* = 2740).

0–5.9 Months				6–11.9 Months				12–23.9 Months			
Rank	Food Category	% of Daily Intake	% Cumulative	Rank	Food Category	% of Daily Intake	% Cumulative	Rank	Food Category	% of Daily Intake	% Cumulative
1	Infant formulas	74.3	74.3	1	Infant formulas	53.4	53.4	1	Milk	53.6	53.6
2	Human milk	18.0	92.3	2	Baby foods	18.4	71.8	2	Cheese	6.9	60.5
3	Baby foods	7.2	99.5	3	Milk	8.2	80.0	3	100% juice	3.7	64.1
				4	Human milk	5.9	85.9	4	Yoghurt	3.1	67.2
				5	Cheese	2.1	88.0	5	Mixed dishes—grain-based	2.9	70.1
				6	Yoghurt	1.9	89.8	6	Baby foods	2.7	72.8
				7	Mixed dishes—grain-based	1.1	90.9	7	Infant formulas	2.4	75.2
								8	Flavored milk	2.3	77.5
								9	Breads, rolls, tortillas	2.0	79.5
								10	Dairy drinks and substitutes	1.9	81.4
								12	Cooked cereals	1.6	84.8
								13	Eggs	1.3	86.2
								14	Mixed dishes—Pizza	1.2	87.4
								15	Plain water	1.2	88.6
								16	Sugar-sweetened beverages	1.0	89.6
								17	Quick breads & bread products	1.0	90.5

^a^ only WWEIA sub-major food categories that contributed ≥1% to daily intake are reported.

**Table 16:** Food sources of magnesium among US infants and toddlers aged 0–23.9 months by age group: NHANES 2005–2012 (*n* = 2740).

0–5.9 Months				6–11.9 Months				12–23.9 Months			
Rank	Food Category	% of Daily Intake	% Cumulative	Rank	Food Category	% of Daily Intake	% Cumulative	Rank	Food Category	% of Daily Intake	% Cumulative
1	Infant formulas	66.9	66.9	1	Baby foods	34.0	34.0	1	Milk	27.9	27.9
2	Baby foods	16.6	83.5	2	Infant formulas	32.3	66.3	2	Fruits	6.5	34.5
3	Human milk	15.0	98.5	3	Milk	4.4	70.7	3	100% juice	5.8	40.2
				4	Fruits	3.7	74.4	4	Mixed dishes—grain-based	4.9	45.1
				5	Human milk	3.3	77.7	5	Baby foods	3.8	48.9
				6	Mixed dishes—grain-based	2.2	79.9	6	Breads, rolls, tortillas	3.8	52.7
				7	Vegetables, excluding potatoes	1.8	81.7	7	Ready-to-eat cereals	3.2	55.9
				8	100% juice	1.6	83.3	8	Plant-based protein foods	3.2	59.1
				9	Plant-based protein foods	1.6	84.8	9	Vegetables, excluding potatoes	2.8	61.9
				10	Ready-to-eat cereals	1.4	86.3	10	Poultry	2.6	64.6
				11	Baby beverages	1.4	87.6	11	Cooked cereals	2.5	67.1
				12	Mixed dishes—Soups	1.2	88.9	12	Sugar-sweetened beverages	2.2	69.3
				13	Breads, rolls, tortillas	1.2	90.0	13	Dairy drinks and substitutes	2.1	71.3
				14	Yoghurt	1.1	91.1	14	Mixed dishes—meat, poultry, seafood	2.0	73.3
								15	Savory snacks	1.9	75.2
								16	Sweet bakery products	1.9	77.1
								17	White potatoes	1.9	79.0
								18	Yoghurt	1.8	80.7
								19	Plain water	1.7	82.4
								20	Flavored milk	1.6	84.0
								21	Cheese	1.5	85.5
								22	Mixed dishes—Soups	1.5	87.0
								23	Infant formulas	1.4	88.5
								24	Eggs	1.2	89.7
								25	Cured meats/poultry	1.1	90.8
								26	Crackers	1.1	91.9

^a^ only WWEIA sub-major food categories that contributed ≥1% to daily intake are reported.

**Table 17:** Food sources of iron among US infants and toddlers aged 0–23.9 months by age group: NHANES 2005–2012 (*n* = 2740).

0–5.9 Months				6–11.9 Months				12–23.9 Months			
Rank	Food Category	% of Daily Intake	% Cumulative	Rank	Food Category	% of Daily Intake	% Cumulative	Rank	Food Category	% of Daily Intake	% Cumulative
1	Infant formulas	77.4	77.4	1	Infant formulas	44.8	44.8	1	Ready-to-eat cereals	18.8	18.8
2	Baby foods	21.4	98.8	2	Baby foods	43.1	87.9	2	Baby foods	14.5	33.3
				3	Ready-to-eat cereals	3.1	91.0	3	Breads, rolls, tortillas	5.9	39.2
				4	Mixed dishes—grain-based	1.0	91.9	4	Mixed dishes—grain-based	5.7	44.9
								5	Cooked cereals	5.5	50.4
								6	Infant formulas	4.7	55.1
								7	Sweet bakery products	4.5	59.6
								8	100% juice	3.1	62.7
								9	Crackers	2.8	65.5
								10	Fruits	2.5	67.9
								11	Eggs	2.3	70.3
								12	Quick breads & bread products	2.3	72.6
								13	Mixed dishes—meat, poultry, seafood	2.3	74.8
								14	Mixed dishes—Soups	2.1	77.0
								15	Vegetables, excluding potatoes	1.9	78.9
								16	Plant-based protein foods	1.8	80.7
								17	Poultry	1.8	82.5
								18	Savory snacks	1.7	84.3
								19	Cured meats/poultry	1.5	85.8
								20	Milk	1.5	87.3
								21	Mixed dishes—Pizza	1.4	88.6
								22	Sugar-sweetened beverages	1.3	89.9
								23	Meats	1.0	90.9
								24	Cooked grains	1.0	91.9

^a^ only WWEIA sub-major food categories that contributed ≥1% to daily intake are reported.

**Table 18:** Food sources of zinc among US infants and toddlers aged 0–23.9 months by age group: NHANES 2005–2012 (*n* = 2740).

0–5.9 Months				6–11.9 Months				12–23.9 Months			
Rank	Food Category	% of Daily Intake	% Cumulative	Rank	Food Category	% of Daily Intake	% Cumulative	Rank	Food Category	% of Daily Intake	% Cumulative
1	Infant formulas	85.7	85.7	1	Infant formulas	58.8	58.8	1	Milk	27.2	27.2
2	Human milk	10.7	96.4	2	Baby foods	15.8	74.6	2	Ready-to-eat cereals	11.8	39.1
3	Baby foods	3.4	99.8	3	Ready-to-eat cereals	4.1	78.7	3	Mixed dishes—grain-based	4.9	44.0
				4	Human milk	3.3	82.0	4	Cheese	4.1	48.1
				5	Milk	3.0	85.0	5	Mixed dishes—meat, poultry, seafood	3.8	51.9
				6	Plant-based protein foods	1.8	86.9	6	Cured meats/poultry	3.5	55.4
				7	Mixed dishes—grain-based	1.7	88.5	7	Infant formulas	3.4	58.7
				8	Mixed dishes—meat, poultry, seafood	1.1	89.6	8	Poultry	3.2	62.0
				9	Yoghurt	1.0	90.6	9	Baby foods	3.1	65.1
								10	Meats	3.1	68.2
								11	Eggs	2.7	70.9
								12	Breads, rolls, tortillas	2.6	73.5
								13	Yoghurt	2.2	75.7
								14	Plant-based protein foods	2.1	77.8
								15	Cooked cereals	1.6	79.4
								16	Fruits	1.5	80.9
								17	Mixed dishes—Soups	1.5	82.4
								18	Flavored milk	1.4	83.8
								19	Vegetables, excluding potatoes	1.4	85.2
								20	Mixed dishes—Pizza	1.4	86.5
								21	Savory snacks	1.3	87.8
								22	Sweet bakery products	1.2	89.1
								23	Mixed dishes—Sandwiches (single code)	1.0	90.1

^a^ only WWEIA sub-major food categories that contributed ≥1% to daily intake are reported.

Infant formulas were also the major food source of all micronutrients (except magnesium) for 6–11.9 month old infants (Table 9, Table 10, Table 11, Table 12, Table 13, Table 14, Table 15, Table 16, Table 17 and Table 18). With the exception of vitamin D, the second ranked food source for all micronutrients were baby foods. Cumulatively infant formulas and baby foods accounted for 74.3% of vitamin A, 59.5% of folate, 66.9% of vitamin C, 87.9% of vitamin E, 62.0% of potassium, 71.8% of calcium, 66.3% of magnesium, 87.9% of iron and 74.6% of zinc intake. In the case of vitamin D, milk (8.3%) was the second ranked food source. Additional information on the types of baby foods that contributed to micronutrient intakes can be found in the supplemental Tables S8–S17. For folate, iron and zinc, ready-to-eat cereals were ranked as the third food source followed by either grain based mixed dishes or human milk. For vitamins A, D and E human milk was ranked as the third food source, whereas for potassium, calcium and magnesium, milk was ranked as the third food source.

Overall among toddlers, milk, and ready-to-eat cereals were the most important contributors to micronutrient intakes (Table 9, Table 10, Table 11, Table 12, Table 13, Table 14, Table 15, Table 16, Table 17 and Table 18). With the exception of iron and vitamin C, milk was among the top three ranked food groups for all other micronutrients: vitamin A (37.4%), folate (10.7%), vitamin D (74.0%), vitamin E (7.3%), potassium (34.8%), calcium (53.6%), magnesium (27.9%) and zinc (27.2%). Whereas ready-to-eat cereals were important food sources for vitamin A (8.1%), folate (21.6%), iron (18.8%) and zinc (11.8%). Other notable food sources contributing more than 5% to nutrient intakes were vegetables -excluding potatoes for vitamin A, grain based mixed dishes and breads, rolls and tortillas for folate; grain based mixed dishes, infant formula, milk, plant-based protein, baby foods, fruits and savory snacks for vitamin E; 100% juice and fruits for vitamin C, potassium and magnesium; cheese for calcium and baby foods, breads, rolls and tortillas, grain based mixed dishes and cooked cereals for iron.

## 4. Discussion

In this study the contribution of food and beverages to energy and nutrient intakes in U.S. infants and toddlers during 2005–2012 are reported. To our knowledge this is the first update of this information since the 2002 Feeding Infants and Toddlers Study (FITS) [10]. It is difficult to make direct comparisons to the earlier work of Fox *et al.* [10] because we did not disaggregate foods, we did not include vitamin and mineral supplements and we utilized a different food group classification system and food composition database.

Overall, in infants aged 0–5.9 months infant formulas provided the majority of total energy and nutrient needs, followed by human milk. This is not surprising, given that less than half (41.5%) of 0–5.9 months olds received breastmilk on the day of the dietary recall. Likewise in infants aged 6 to 11.9 months infant formulas were an important contributor to nutrient intakes (range 38%–85% across nutrients).

The 2002 FITS reported similar contributions of infant formulas to nutrient intakes [10]. The American Academy of Pediatrics recommends exclusive breastfeeding until about 6 months of age. Following this complimentary foods should be introduced and breastfeeding continued for 1 year or longer [24]. National estimates indicate breastfeeding rates in the US have increased over time. In 2003 the proportion of children who had ever been breastfed was 72.6%, compared to 79.2% in 2011 [25]. Similarly there was an increase in the proportion of children who were exclusively breastfed through to 3 months, 29.6% *vs.* 40.7% and through to 6 months, 10.3% *vs.* 18.8%, in 2003 and 2011, respectively [25]. Whilst such figures are promising, our findings indicated that during 2005–2012 more than two thirds of infants aged 0–11.9 months were not breastfed on the day of the dietary recall (67.7%). Therefore, efforts that support and encourage breastfeeding during the first year of life are needed.

After around the first 6 months of life the infant requires complimentary foods in addition to breast milk to help meet nutrient requirements for growth and development and to enable the development of eating skills [26]. Iron is of particular importance during this time as the infant’s iron stores, which are laid down during gestation, are declining [27]. The 2008 FITS found that 12% of infants aged 6 to 11 months did not meet the Estimated Average Requirement for iron [7]. National estimates in toddlers aged 12 to 35 months indicated iron deficiency occurred in 9.2% of toddlers and iron deficiency anemia in 2.1%, however estimates were higher in certain ethic and socioeconomic subgroups [27]. Given the detrimental consequences of iron deficiency disorders on cognitive and neurological development [28], it is recommended that iron fortified cereals and meat/poultry be introduced during complimentary feeding, furthermore an iron fortified formula should always be used for formula fed infants [27]. Our findings show that both infant formulas and commercial baby foods were the major food sources of iron in infants aged 6–11.9 months. Within baby foods, cereal-based foods (39%) were by far the most important source of iron compared to vegetable- (0.4%), meat- (0.1%) or fruit-based baby foods (0.1%). In toddlers, a similar trend was seen whereby non-heme sources of iron, *i.e.*, cereal-based foods, which included ready-to-eat cereals, breads, grain based mixed dishes and baby cereal accounted for the majority (about 55%) of iron intake. Comparatively, foods naturally rich in highly bioavailable heme iron such as poultry (1.8%) and meats (1.0%) contributed far less to iron intakes. The importance of non-heme food sources relative to heme food sources of iron was also demonstrated in the 2002 FITS [10]. It appears that there is a need to reinforce the message to parents on the benefits and appropriateness of introducing meat as a complimentary food [29]. Meat is also rich in bioavailable zinc, which has been shown to be a limiting nutrient in some infants [7]. In industrialized countries, including the U.S., meat-based interventions have been shown to improve iron and zinc intakes in infants and toddlers [29,30,31] and appear to have a positive effect on iron status in the first two years of life [30,32]. Importantly, the focus should be on the inclusion of iron rich lean red, non-processed meats [33].

As young children have a small stomach capacity and high nutrient needs relative to their energy needs, complimentary foods should be nutrient dense (*i.e.*, relatively low in calories and high in vitamins and minerals). There is little room for the inclusion of energy rich foods and beverages which are of low nutritional quality [26]. Whilst it is reassuring to see some nutrient dense foods amongst the top food sources of total energy for infants 6.0–11.9 months, *i.e.*, milk and fruits, and toddlers, *i.e.*, milk, fruits, poultry, cheese and eggs, it is also concerning that many poorer food choices make significant contributions to total energy intake. For example, sugar-sweetened beverages, savory snacks and sweet bakery products (*i.e.*, cookies, cakes, doughnuts), with the latter also contributing to saturated fat intakes. As higher total energy intakes during the complimentary feeding period have been linked to increased BMI in childhood [34], it is important that energy rich foods, which provide no nutritional benefit are limited. In line with this, the American Academy of Pediatrics advises that the introduction of sugar-sweetened beverages should be avoided [35].

Also of note is the relative contribution of 100% juice, which includes fruit and vegetables juices, to total energy intakes. In toddlers, these beverages ranked as the 2nd source of total energy (5.9%), and these findings are similar to those previously reported by Fox *et al.* [10]. These 100% juices are often fortified with vitamins, such as calcium and iron, and in this study these beverages made important contributions to intakes of folate, vitamin C, potassium, magnesium, calcium and iron. Whilst the inclusion of 100% juices in the diets of toddlers in limited quantities may provide some nutritional benefit, the American Academy of Pediatrics recommends that intake be limited to no more than 4–6 ounces/day [35]. Furthermore, parents should be reminded that the inclusion of whole fruits or vegetables over juice, will also help with increasing fiber intakes in toddlers, which are generally low [7]. Additionally greater intakes of 100% juice, as well as sugar-sweetened beverages, have been associated with lower intakes of calcium, indicating these beverages may displace milk [36]. However, other studies have shown that 100% fruit juice is associated with better nutrient intake and diet quality [37]. In summary, the message of appropriate beverage choices needs to be reinforced amongst parents and health professionals with a focus on encouraging breast milk or infant formula during the first year of life, followed by water, milk and no more than 4–6 ounces of 100% juice during the toddler years [35].

Our findings also indicated that the inclusion of cured meats and poultry was relatively common in the diets of toddlers. This food group, which includes cold cuts, bacon, frankfurters and sausages, contributed to a number of nutrients including, total energy, protein, saturated fat, potassium and zinc. These foods are generally high in sodium and have previously been identified as contributing to the high intakes of sodium reported in U.S. infants and toddlers [11]. Messages are needed to encourage a switch from these types of meat products to non-processed, lean meats, which are naturally rich in zinc, iron and potassium and low in sodium and saturated fat.

Low fiber intakes in U.S. toddlers have previously been reported [38]. In this study, fruit, vegetables, grain based products and breads were found to be the top contributors to toddlers’ fiber intakes. These foods which are high in fiber also contain an array of other important micronutrients, some of which are also shortfall nutrients in toddlers’ diets, for example potassium [7]. To improve intakes of fiber and potassium a variety of fruits and vegetables should be encouraged. In addition care should be taken to select whole grain varieties of breads and cereals, which are rich in fiber and potassium.

Previously Malouf *et al.* [11] identified that commercial baby foods contributed a relatively high proportion (9%) of sodium to the diets of U.S. infants aged 6–11.9 months. In this study, within this age group of infants commercial baby foods were an important source of most micronutrients, ranking as the 2nd source after infant formulas. As the majority of U.S. children aged 1–3 years exceed the upper level for sodium intake [8] companies are encouraged to lower the sodium content of commercial baby foods.

The major strengths of this study include the use of a nationally representative sample of U.S. infants and toddlers and the robust and standardized collection of dietary data within the NHANES. To determine food group contributions the WWEI food group categories were used. An advantage of this food group classification system is that similar foods and beverages are grouped together based on usage and nutrient content, which can be useful for providing practical advice to parents and health professionals about the types of foods that should be encouraged in the diets of infants and toddlers. Alternatively, as this food group classification system does not disaggregate food items into ingredients, comparisons with previous work is limited [10]. Other limitations include the potential for the proxy to over or under-report foods consumed by their child and the imputation of estimated amounts of human milk for breastfed infants.

## 5. Conclusions

In conclusion, this study provides detailed information on the food sources of total energy and a range of nutrients which require special attention during early life. This information can be used to inform targeted dietary strategies within public health initiatives to improve the diets of infants and toddlers and to guide parents regarding appropriate food selection. Finally, this information is useful to monitor changes in eating habits of U.S. infants and toddlers over time.

## Supplementary Files

Supplementary File 1
