# Supplementary material for: Food Sources of Total Energy and Nutrients among U.S. Infants and Toddlers: National Health and Nutrition Examination Survey 2005–2012
Source: Nutrients. 2015 Aug 14;7(8):6797–836. doi: 10.3390/nu7085310 (PMC4555149; doi:10.3390/nu7085310)
Supplement: Supplementary File 1 [file nutrients-07-05310-s001.docx]

**Supplementary Information**

**Table S1.** Food sources of total energy among U.S. infants and toddlers aged 0–23.9 months by age group: NHANES 2005-12 (*n* = 2740) ^a, b^.

| **WWEIA Minor Food category code** | **Food category** | **% of daily intake** | | |
| --- | --- | --- | --- | --- |
|  |  | **0–5.9 mo (*n* = 765)** | **6–11.9 mo (*n* = 854)** | **12–23.9 mo  (*n* = 1121)** |
| **1002** | **Milk, whole** | **0.0** | **2.5** | **18.1** |
| **1004** | **Milk, reduced fat** | **0.0** | **0.4** | **3.8** |
| 1006 | Milk, low-fat | 0.0 | 0.2 | 0.3 |
| 1008 | Milk, non-fat | 0.0 | 0.0 | 0.3 |
| 1202 | Flavored milk, whole | 0.0 | 0.0 | 0.8 |
| 1204 | Flavored milk, reduced fat | 0.0 | 0.0 | 0.3 |
| 1206 | Flavored milk, low-fat | 0.0 | 0.0 | 0.2 |
| 1208 | Flavored milk, non-fat | 0.0 | 0.0 | 0.0 |
| 1402 | Milk shakes & other dairy drinks | 0.0 | 0.0 | 0.0 |
| 1404 | Milk substitutes | 0.0 | 0.2 | 0.8 |
| **1602** | **Cheese** | **0.0** | **0.7** | **2.5** |
| 1604 | Cottage/ricotta cheese | 0.0 | 0.0 | 0.1 |
| 1802 | Yogurt, whole & reduced fat | 0.0 | 0.5 | 0.8 |
| **1804** | **Yogurt, low-fat & non-fat** | **0.0** | **0.5** | **1.0** |
| 2002 | Beef, excludes ground | 0.0 | 0.1 | 0.3 |
| 2004 | Ground beef | 0.0 | 0.2 | 0.4 |
| 2006 | Pork | 0.0 | 0.0 | 0.1 |
| 2008 | Lamb, goat, game | 0.0 | 0.0 | 0.0 |
| 2010 | Liver & organ meats | 0.0 | 0.0 | 0.0 |
| **2202** | **Chicken, whole pieces** | **0.0** | **0.2** | **1.5** |
| **2204** | **Chicken patties, nuggets & tenders** | **0.0** | **0.2** | **2.0** |
| 2206 | Turkey, duck, other poultry | 0.0 | 0.1 | 0.1 |
| 2402 | Fish | 0.0 | 0.0 | 0.2 |
| 2404 | Shellfish | 0.0 | 0.0 | 0.0 |
| **2502** | **Eggs & omelettes** | **0.0** | **0.6** | **2.2** |
| 2602 | Cold cuts & cured meats | 0.0 | 0.1 | 0.5 |
| 2604 | Bacon | 0.0 | 0.0 | 0.1 |
| 2606 | Frankfurters | 0.0 | 0.3 | 1.3 |
| 2608 | Sausages | 0.0 | 0.3 | 0.6 |
| 2802 | Beans, peas, legumes | 0.0 | 0.5 | 0.7 |
| 2804 | Nuts & seeds | 0.0 | 0.1 | 0.8 |
| 2806 | Processed soy products | 0.0 | 0.1 | 0.1 |
| 3002 | Meat mixed dishes | 0.0 | 0.4 | 0.9 |
| 3004 | Poultry mixed dishes | 0.0 | 0.2 | 1.0 |
| 3006 | Seafood mixed dishes | 0.0 | 0.0 | 0.2 |
| 3202 | Rice mixed dishes | 0.0 | 0.3 | 0.5 |
| **3204** | **Pasta mixed dishes, excludes macaroni & cheese** | **0.0** | **1.1** | **2.6** |
| **3206** | **Macaroni & cheese** | **0.0** | **0.9** | **2.2** |
| 3208 | Turnovers & other grain-based items | 0.0 | 0.0 | 0.1 |
| 3402 | Fried rice & lo/chow mein | 0.0 | 0.0 | 0.3 |
| 3404 | Stir-fry & soy-based sauce mixtures | 0.0 | 0.1 | 0.1 |
| 3406 | Egg rolls, dumplings, sushi | 0.0 | 0.0 | 0.0 |
| 3502 | Burritos & tacos | 0.0 | 0.0 | 0.3 |
| 3504 | Nachos | 0.0 | 0.0 | 0.0 |
| 3506 | Other Mexican mixed dishes | 0.0 | 0.1 | 0.5 |
| **3602** | **Pizza** | **0.0** | **0.1** | **1.5** |
| 3702 | Burgers (single code) | 0.0 | 0.1 | 0.3 |
| 3704 | Chicken/turkey sandwiches (single code) | 0.0 | 0.0 | 0.1 |
| 3706 | Egg/breakfast sandwiches (single code) | 0.0 | 0.0 | 0.0 |
| 3708 | Other sandwiches (single code) | 0.0 | 0.0 | 0.4 |
| **3802** | **Soups** | **0.0** | **1.0** | **1.5** |
| 4002 | Rice | 0.0 | 0.4 | 0.6 |
| 4004 | Pasta, noodles, cooked grains | 0.0 | 0.0 | 0.2 |
| **4202** | **Yeast breads** | **0.0** | **0.7** | **2.5** |
| 4204 | Rolls & buns | 0.0 | 0.1 | 0.6 |
| 4206 | Bagels & English muffins | 0.0 | 0.0 | 0.2 |
| 4208 | Tortillas | 0.0 | 0.2 | 0.4 |
| 4402 | Biscuits, muffins, quick breads | 0.0 | 0.2 | 0.7 |
| **4404** | **Pancakes, waffles, French toast** | **0.0** | **0.2** | **1.0** |
| **4602** | **Ready-to-eat cereal, higher sugar (>21.2g/100g)** | **0.0** | **0.2** | **1.1** |
| **4604** | **Ready-to-eat cereal, lower sugar (≤21.2g/100g)** | **0.0** | **0.5** | **1.2** |
| **4802** | **Oatmeal** | **0.0** | **0.3** | **1.1** |
| 4804 | Grits & other cooked cereals | 0.0 | 0.1 | 0.3 |
| 5002 | Potato chips | 0.0 | 0.0 | 0.6 |
| **5004** | **Tortilla, corn, other chips** | **0.0** | **0.4** | **1.2** |
| 5006 | Popcorn | 0.0 | 0.0 | 0.1 |
| 5008 | Pretzels/snack mix | 0.0 | 0.1 | 0.5 |
| **5202** | **Crackers, excludes saltines** | **0.0** | **0.6** | **2.2** |
| 5204 | Saltine crackers | 0.0 | 0.2 | 0.3 |
| 5402 | Cereal bars | 0.0 | 0.1 | 0.6 |
| 5404 | Nutrition bars | 0.0 | 0.0 | 0.0 |
| 5502 | Cakes & pies | 0.0 | 0.2 | 0.7 |
| **5504** | **Cookies & brownies** | **0.0** | **1.5** | **3.0** |
| 5506 | Doughnuts, sweet rolls, pastries | 0.0 | 0.2 | 0.9 |
| 5702 | Candy containing chocolate | 0.0 | 0.0 | 0.3 |
| **5704** | **Candy not containing chocolate** | **0.0** | **0.1** | **1.0** |
| 5802 | Ice cream & frozen dairy desserts | 0.0 | 0.2 | 0.8 |
| 5804 | Pudding | 0.0 | 0.1 | 0.2 |
| 5806 | Gelatins, ices, sorbets | 0.0 | 0.1 | 0.2 |
| 6002 | Apples | 0.0 | 0.5 | 0.9 |
| **6004** | **Bananas** | **0.0** | **1.1** | **1.6** |
| 6006 | Grapes | 0.0 | 0.1 | 0.4 |
| 6008 | Peaches & nectarines | 0.0 | 0.2 | 0.3 |
| 6010 | Berries | 0.0 | 0.1 | 0.2 |
| 6012 | Citrus fruits | 0.0 | 0.1 | 0.3 |
| 6014 | Melons | 0.0 | 0.1 | 0.2 |
| 6016 | Dried fruits | 0.0 | 0.1 | 0.3 |
| 6018 | Other fruits & fruit salads | 0.0 | 0.2 | 0.7 |
| 6402 | Tomatoes | 0.0 | 0.0 | 0.0 |
| 6404 | Carrots | 0.0 | 0.0 | 0.1 |
| 6406 | Other red & orange vegetables | 0.0 | 0.1 | 0.1 |
| 6408 | Dark green vegetables, excludes lettuce | 0.0 | 0.0 | 0.1 |
| 6410 | Lettuce & lettuce salads | 0.0 | 0.0 | 0.0 |
| 6412 | String beans | 0.0 | 0.0 | 0.1 |
| 6414 | Onions | 0.0 | 0.0 | 0.0 |
| 6416 | Corn | 0.0 | 0.1 | 0.2 |
| 6418 | Other starchy vegetables | 0.0 | 0.2 | 0.2 |
| 6420 | Other vegetables & combinations | 0.0 | 0.2 | 0.2 |
| 6422 | Vegetable mixed dishes | 0.0 | 0.0 | 0.2 |
| 6802 | White potatoes, baked or boiled | 0.0 | 0.0 | 0.1 |
| **6804** | **French fries & other fried white potatoes** | **0.0** | **0.3** | **1.3** |
| 6806 | Mashed potatoes & white potato mixtures | 0.0 | 0.4 | 0.5 |
| 7002 | Citrus juice | 0.0 | 0.1 | 0.8 |
| **7004** | **Apple juice** | **0.1** | **0.7** | **2.8** |
| **7006** | **Other fruit juice** | **0.1** | **0.7** | **2.4** |
| 7008 | Vegetable juice | 0.0 | 0.0 | 0.0 |
| 7102 | Diet soft drinks | 0.0 | 0.0 | 0.0 |
| 7106 | Other diet drinks | 0.0 | 0.0 | 0.0 |
| 7202 | Soft drinks | 0.0 | 0.0 | 0.4 |
| **7204** | **Fruit drinks** | **0.0** | **0.3** | **2.4** |
| 7206 | Sport & energy drinks | 0.0 | 0.1 | 0.2 |
| 7208 | Nutritional beverages | 0.0 | 0.0 | 0.2 |
| 7302 | Coffee | 0.0 | 0.0 | 0.0 |
| 7304 | Tea | 0.0 | 0.0 | 0.2 |
| 7702 | Tap water | 0.0 | 0.0 | 0.0 |
| 7704 | Bottled water | 0.0 | 0.0 | 0.0 |
| 7802 | Flavored or carbonated water | 0.0 | 0.0 | 0.0 |
| 7804 | Enhanced or fortified water | 0.0 | 0.0 | 0.0 |
| 8002 | Butter & animal fats | 0.0 | 0.1 | 0.2 |
| 8004 | Margarine | 0.0 | 0.1 | 0.2 |
| 8006 | Cream cheese, sour cream, whipped cream | 0.0 | 0.0 | 0.1 |
| 8008 | Cream & cream substitutes | 0.0 | 0.0 | 0.1 |
| 8010 | Mayonnaise | 0.0 | 0.0 | 0.0 |
| 8012 | Salad dressings & vegetable oils | 0.0 | 0.0 | 0.1 |
| 8402 | Tomato-based condiments | 0.0 | 0.0 | 0.0 |
| 8404 | Soy-based condiments | 0.0 | 0.0 | 0.0 |
| 8406 | Mustard & other condiments | 0.0 | 0.0 | 0.0 |
| 8408 | Olives, pickles, pickled vegetables | 0.0 | 0.0 | 0.0 |
| 8410 | Pasta sauces, tomato-based | 0.0 | 0.0 | 0.0 |
| 8412 | Dips, gravies, other sauces | 0.0 | 0.0 | 0.1 |
| 8802 | Sugars & honey | 0.0 | 0.0 | 0.1 |
| 8804 | Sugar substitutes | 0.0 | 0.0 | 0.0 |
| 8806 | Jams, syrups, toppings | 0.0 | 0.0 | 0.4 |
| **9002** | **Baby food: cereals** | **2.6** | **6.3** | **0.8** |
| **9004** | **Baby food: fruit** | **0.5** | **3.9** | **0.4** |
| **9006** | **Baby food: vegetable** | **0.4** | **2.1** | **0.2** |
| **9008** | **Baby food: meat & dinners** | **0.2** | **2.4** | **0.7** |
| 9010 | Baby food: yogurt | 0.0 | 0.6 | 0.1 |
| **9012** | **Baby food: snacks & sweets** | **0.0** | **1.2** | **0.4** |
| **9202** | **Baby juice** | **0.3** | **1.5** | **0.4** |
| 9204 | Baby water | 0.0 | 0.0 | 0.0 |
| **9402** | **Formula, ready-to-feed** | **2.2** | **2.6** | **0.6** |
| **9404** | **Formula, prepared from powder** | **57.9** | **41.1** | **1.4** |
| **9406** | **Formula, prepared from concentrate** | **5.2** | **3.4** | **0.1** |
| **9602** | **Human milk** | **30.1** | **9.9** | **1.3** |
| 9999 | Not included in a food category | 0.0 | 0.0 | 0.1 |
|  | Total | 100 | 100 | 100 |

^a^ Contributions from all minor WWEIA food categories are reported. Those in bold contribute ≥1% to daily intake in at least one age group; ^b^ On the day of the 24-hr recall no subjects consumed any food items that fell within the minor food categories of 7104 Diet sport and energy drinks; 7502 Beer; 7504 Wine; 7506 Liquor. Therefore these minor food categories are omitted from all supplementary tables.

**Table S2.** Food sources of protein among U.S. infants and toddlers aged 0–23.9 months by age group: NHANES 2005-12 (*n* = 2740) ^a^.

| **WWEIA Minor Food category code** | **Food category** | **% of daily intake** | | |
| --- | --- | --- | --- | --- |
|  |  | **0–5.9 mo (*n* = 765)** | **6–11.9 mo (*n* = 854)** | **12–23.9 mo  (*n* = 1121)** |
| **1002** | **Milk, whole** | **0.0** | **5.3** | **24.9** |
| **1004** | **Milk, reduced fat** | **0.0** | **1.1** | **6.5** |
| 1006 | Milk, low-fat | 0.0 | 0.6 | 0.6 |
| 1008 | Milk, non-fat | 0.0 | 0.0 | 0.7 |
| 1202 | Flavored milk, whole | 0.0 | 0.1 | 0.8 |
| 1204 | Flavored milk, reduced fat | 0.0 | 0.0 | 0.3 |
| 1206 | Flavored milk, low-fat | 0.0 | 0.0 | 0.2 |
| 1208 | Flavored milk, non-fat | 0.0 | 0.0 | 0.0 |
| 1402 | Milk shakes & other dairy drinks | 0.0 | 0.0 | 0.0 |
| **1404** | **Milk substitutes** | **0.0** | **0.3** | **1.0** |
| **1602** | **Cheese** | **0.0** | **1.9** | **4.5** |
| 1604 | Cottage/ricotta cheese | 0.0 | 0.2 | 0.4 |
| 1802 | Yogurt, whole & reduced fat | 0.0 | 0.8 | 0.8 |
| **1804** | **Yogurt, low-fat & non-fat** | **0.0** | **0.8** | **1.2** |
| 2002 | Beef, excludes ground | 0.0 | 0.3 | 0.8 |
| 2004 | Ground beef | 0.0 | 0.6 | 0.9 |
| 2006 | Pork | 0.0 | 0.0 | 0.5 |
| 2008 | Lamb, goat, game | 0.0 | 0.0 | 0.0 |
| 2010 | Liver & organ meats | 0.0 | 0.0 | 0.0 |
| **2202** | **Chicken, whole pieces** | **0.0** | **1.5** | **5.3** |
| **2204** | **Chicken patties, nuggets & tenders** | **0.0** | **0.4** | **2.9** |
| 2206 | Turkey, duck, other poultry | 0.0 | 0.5 | 0.3 |
| 2402 | Fish | 0.0 | 0.1 | 0.5 |
| 2404 | Shellfish | 0.0 | 0.0 | 0.0 |
| **2502** | **Eggs & omelets** | **0.0** | **1.7** | **3.9** |
| **2602** | **Cold cuts & cured meats** | **0.0** | **0.6** | **1.4** |
| 2604 | Bacon | 0.0 | 0.1 | 0.2 |
| **2606** | **Frankfurters** | **0.0** | **0.5** | **1.4** |
| 2608 | Sausages | 0.0 | 0.5 | 0.9 |
| **2802** | **Beans, peas, legumes** | **0.0** | **1.2** | **0.9** |
| 2804 | Nuts & seeds | 0.0 | 0.1 | 0.9 |
| 2806 | Processed soy products | 0.0 | 0.1 | 0.2 |
| **3002** | **Meat mixed dishes** | **0.0** | **1.2** | **2.0** |
| **3004** | **Poultry mixed dishes** | **0.0** | **0.7** | **1.8** |
| 3006 | Seafood mixed dishes | 0.0 | 0.1 | 0.4 |
| 3202 | Rice mixed dishes | 0.0 | 0.3 | 0.4 |
| **3204** | **Pasta mixed dishes, excludes macaroni & cheese** | **0.0** | **1.9** | **2.8** |
| **3206** | **Macaroni & cheese** | **0.0** | **1.3** | **2.3** |
| 3208 | Turnovers & other grain-based items | 0.0 | 0.1 | 0.1 |
| 3402 | Fried rice & lo/chow mein | 0.0 | 0.0 | 0.4 |
| 3404 | Stir-fry & soy-based sauce mixtures | 0.0 | 0.3 | 0.2 |
| 3406 | Egg rolls, dumplings, sushi | 0.0 | 0.0 | 0.0 |
| 3502 | Burritos & tacos | 0.0 | 0.0 | 0.4 |
| 3504 | Nachos | 0.0 | 0.0 | 0.0 |
| 3506 | Other Mexican mixed dishes | 0.0 | 0.1 | 0.5 |
| **3602** | **Pizza** | **0.0** | **0.2** | **1.7** |
| 3702 | Burgers (single code) | 0.0 | 0.3 | 0.4 |
| 3704 | Chicken/turkey sandwiches (single code) | 0.0 | 0.0 | 0.2 |
| 3706 | Egg/breakfast sandwiches (single code) | 0.0 | 0.0 | 0.0 |
| 3708 | Other sandwiches (single code) | 0.0 | 0.0 | 0.4 |
| **3802** | **Soups** | **0.0** | **2.1** | **2.1** |
| 4002 | Rice | 0.0 | 0.3 | 0.3 |
| 4004 | Pasta, noodles, cooked grains | 0.0 | 0.1 | 0.2 |
| **4202** | **Yeast breads** | **0.0** | **1.0** | **2.4** |
| 4204 | Rolls & buns | 0.0 | 0.1 | 0.5 |
| 4206 | Bagels & English muffins | 0.0 | 0.0 | 0.2 |
| 4208 | Tortillas | 0.0 | 0.3 | 0.3 |
| 4402 | Biscuits, muffins, quick breads | 0.0 | 0.1 | 0.3 |
| 4404 | Pancakes, waffles, French toast | 0.0 | 0.2 | 0.7 |
| 4602 | Ready-to-eat cereal, higher sugar (>21.2g/100g) | 0.0 | 0.1 | 0.5 |
| 4604 | Ready-to-eat cereal, lower sugar (≤21.2g/100g) | 0.0 | 0.6 | 0.8 |
| **4802** | **Oatmeal** | **0.0** | **0.3** | **1.0** |
| 4804 | Grits & other cooked cereals | 0.0 | 0.2 | 0.2 |
| 5002 | Potato chips | 0.0 | 0.0 | 0.2 |
| 5004 | Tortilla, corn, other chips | 0.0 | 0.2 | 0.4 |
| 5006 | Popcorn | 0.0 | 0.0 | 0.0 |
| 5008 | Pretzels/snack mix | 0.0 | 0.1 | 0.3 |
| **5202** | **Crackers, excludes saltines** | **0.0** | **0.4** | **1.0** |
| 5204 | Saltine crackers | 0.0 | 0.1 | 0.2 |
| 5402 | Cereal bars | 0.0 | 0.0 | 0.2 |
| 5404 | Nutrition bars | 0.0 | 0.0 | 0.0 |
| 5502 | Cakes & pies | 0.0 | 0.1 | 0.2 |
| **5504** | **Cookies & brownies** | **0.0** | **0.9** | **1.1** |
| 5506 | Doughnuts, sweet rolls, pastries | 0.0 | 0.1 | 0.3 |
| 5702 | Candy containing chocolate | 0.0 | 0.0 | 0.1 |
| 5704 | Candy not containing chocolate | 0.0 | 0.0 | 0.1 |
| 5802 | Ice cream & frozen dairy desserts | 0.0 | 0.1 | 0.4 |
| 5804 | Pudding | 0.0 | 0.1 | 0.1 |
| 5806 | Gelatins, ices, sorbets | 0.0 | 0.0 | 0.1 |
| 6002 | Apples | 0.0 | 0.1 | 0.1 |
| 6004 | Bananas | 0.0 | 0.5 | 0.5 |
| 6006 | Grapes | 0.0 | 0.0 | 0.1 |
| 6008 | Peaches & nectarines | 0.0 | 0.1 | 0.1 |
| 6010 | Berries | 0.0 | 0.0 | 0.1 |
| 6012 | Citrus fruits | 0.0 | 0.1 | 0.2 |
| 6014 | Melons | 0.0 | 0.0 | 0.1 |
| 6016 | Dried fruits | 0.0 | 0.0 | 0.1 |
| 6018 | Other fruits & fruit salads | 0.0 | 0.1 | 0.1 |
| 6402 | Tomatoes | 0.0 | 0.0 | 0.0 |
| 6404 | Carrots | 0.0 | 0.0 | 0.0 |
| 6406 | Other red & orange vegetables | 0.0 | 0.1 | 0.0 |
| 6408 | Dark green vegetables, excludes lettuce | 0.0 | 0.1 | 0.2 |
| 6410 | Lettuce & lettuce salads | 0.0 | 0.0 | 0.0 |
| 6412 | String beans | 0.0 | 0.1 | 0.2 |
| 6414 | Onions | 0.0 | 0.0 | 0.0 |
| 6416 | Corn | 0.0 | 0.1 | 0.2 |
| 6418 | Other starchy vegetables | 0.0 | 0.3 | 0.2 |
| 6420 | Other vegetables & combinations | 0.0 | 0.2 | 0.2 |
| 6422 | Vegetable mixed dishes | 0.0 | 0.0 | 0.2 |
| 6802 | White potatoes, baked or boiled | 0.0 | 0.0 | 0.1 |
| 6804 | French fries & other fried white potatoes | 0.0 | 0.1 | 0.4 |
| 6806 | Mashed potatoes & white potato mixtures | 0.0 | 0.3 | 0.3 |
| 7002 | Citrus juice | 0.0 | 0.1 | 0.3 |
| 7004 | Apple juice | 0.0 | 0.1 | 0.1 |
| 7006 | Other fruit juice | 0.0 | 0.2 | 0.3 |
| 7008 | Vegetable juice | 0.0 | 0.0 | 0.0 |
| 7102 | Diet soft drinks | 0.0 | 0.0 | 0.0 |
| 7106 | Other diet drinks | 0.0 | 0.0 | 0.0 |
| 7202 | Soft drinks | 0.0 | 0.0 | 0.0 |
| 7204 | Fruit drinks | 0.0 | 0.0 | 0.2 |
| 7206 | Sport & energy drinks | 0.0 | 0.0 | 0.0 |
| 7208 | Nutritional beverages | 0.0 | 0.0 | 0.2 |
| 7302 | Coffee | 0.0 | 0.0 | 0.0 |
| 7304 | Tea | 0.0 | 0.0 | 0.0 |
| 7702 | Tap water | 0.0 | 0.0 | 0.0 |
| 7704 | Bottled water | 0.0 | 0.0 | 0.0 |
| 7802 | Flavored or carbonated water | 0.0 | 0.0 | 0.0 |
| 7804 | Enhanced or fortified water | 0.0 | 0.0 | 0.0 |
| 8002 | Butter & animal fats | 0.0 | 0.0 | 0.0 |
| 8004 | Margarine | 0.0 | 0.0 | 0.0 |
| 8006 | Cream cheese, sour cream, whipped cream | 0.0 | 0.0 | 0.0 |
| 8008 | Cream & cream substitutes | 0.0 | 0.0 | 0.0 |
| 8010 | Mayonnaise | 0.0 | 0.0 | 0.0 |
| 8012 | Salad dressings & vegetable oils | 0.0 | 0.0 | 0.0 |
| 8402 | Tomato-based condiments | 0.0 | 0.0 | 0.0 |
| 8404 | Soy-based condiments | 0.0 | 0.0 | 0.0 |
| 8406 | Mustard & other condiments | 0.0 | 0.0 | 0.0 |
| 8408 | Olives, pickles, pickled vegetables | 0.0 | 0.0 | 0.0 |
| 8410 | Pasta sauces, tomato-based | 0.0 | 0.0 | 0.1 |
| 8412 | Dips, gravies, other sauces | 0.0 | 0.0 | 0.1 |
| 8802 | Sugars & honey | 0.0 | 0.0 | 0.0 |
| 8804 | Sugar substitutes | 0.0 | 0.0 | 0.0 |
| 8806 | Jams, syrups, toppings | 0.0 | 0.0 | 0.0 |
| **9002** | **Baby food: cereals** | **2.9** | **5.8** | **0.6** |
| **9004** | **Baby food: fruit** | **0.2** | **1.3** | **0.1** |
| **9006** | **Baby food: vegetable** | **0.7** | **2.9** | **0.1** |
| **9008** | **Baby food: meat & dinners** | **0.5** | **4.9** | **0.9** |
| 9010 | Baby food: yogurt | 0.0 | 0.5 | 0.1 |
| 9012 | Baby food: snacks & sweets | 0.0 | 0.8 | 0.2 |
| 9202 | Baby juice | 0.0 | 0.1 | 0.0 |
| 9204 | Baby water | 0.0 | 0.0 | 0.0 |
| **9402** | **Formula, ready-to-feed** | **2.6** | **2.4** | **0.4** |
| **9404** | **Formula, prepared from powder** | **64.5** | **36.3** | **0.9** |
| **9406** | **Formula, prepared from concentrate** | **5.8** | **3.0** | **0.0** |
| **9602** | **Human milk** | **22.4** | **5.8** | **0.5** |
| 9999 | Not included in a food category | 0.0 | 0.0 | 0.1 |
|  | Total | 100 | 100 | 100 |

^a^ Contributions from all minor WWEIA food categories are reported. Those in bold contribute ≥1% to daily intake in at least one age group.

**Table S3.** Food sources of total fat among U.S. infants and toddlers aged 0–23.9 months by age group: NHANES 2005-12 (*n* = 2740) ^a^.

| **WWEIA Minor Food category code** | **Food category** | **% of daily intake** | | |
| --- | --- | --- | --- | --- |
|  |  | **0–5.9 mo (*n* = 765)** | **6–11.9 mo (*n* = 854)** | **12–23.9 mo  (*n* = 1121)** |
| **1002** | **Milk, whole** | **0.0** | **3.3** | **25.9** |
| **1004** | **Milk, reduced fat** | **0.0** | **0.4** | **4.0** |
| 1006 | Milk, low-fat | 0.0 | 0.1 | 0.2 |
| 1008 | Milk, non-fat | 0.0 | 0.0 | 0.0 |
| 1202 | Flavored milk, whole | 0.0 | 0.0 | 0.8 |
| 1204 | Flavored milk, reduced fat | 0.0 | 0.0 | 0.2 |
| 1206 | Flavored milk, low-fat | 0.0 | 0.0 | 0.1 |
| 1208 | Flavored milk, non-fat | 0.0 | 0.0 | 0.0 |
| 1402 | Milk shakes & other dairy drinks | 0.0 | 0.0 | 0.0 |
| 1404 | Milk substitutes | 0.0 | 0.1 | 0.7 |
| **1602** | **Cheese** | **0.0** | **1.3** | **4.9** |
| 1604 | Cottage/ricotta cheese | 0.0 | 0.0 | 0.1 |
| 1802 | Yogurt, whole & reduced fat | 0.0 | 0.4 | 0.6 |
| 1804 | Yogurt, low-fat & non-fat | 0.0 | 0.1 | 0.3 |
| 2002 | Beef, excludes ground | 0.0 | 0.1 | 0.3 |
| 2004 | Ground beef | 0.0 | 0.2 | 0.6 |
| 2006 | Pork | 0.0 | 0.0 | 0.2 |
| 2008 | Lamb, goat, game | 0.0 | 0.0 | 0.0 |
| 2010 | Liver & organ meats | 0.0 | 0.0 | 0.0 |
| 2202 | Chicken, whole pieces | 0.0 | 0.2 | 1.8 |
| **2204** | **Chicken patties, nuggets & tenders** | **0.0** | **0.3** | **3.3** |
| 2206 | Turkey, duck, other poultry | 0.0 | 0.1 | 0.1 |
| 2402 | Fish | 0.0 | 0.0 | 0.3 |
| 2404 | Shellfish | 0.0 | 0.0 | 0.0 |
| **2502** | **Eggs & omelets** | **0.0** | **1.1** | **4.3** |
| 2602 | Cold cuts & cured meats | 0.0 | 0.2 | 0.6 |
| 2604 | Bacon | 0.0 | 0.1 | 0.3 |
| **2606** | **Frankfurters** | **0.0** | **0.7** | **2.9** |
| **2608** | **Sausages** | **0.0** | **0.7** | **1.4** |
| 2802 | Beans, peas, legumes | 0.0 | 0.2 | 0.5 |
| **2804** | **Nuts & seeds** | **0.0** | **0.1** | **1.8** |
| 2806 | Processed soy products | 0.0 | 0.1 | 0.1 |
| **3002** | **Meat mixed dishes** | **0.0** | **0.4** | **1.0** |
| **3004** | **Poultry mixed dishes** | **0.0** | **0.2** | **1.0** |
| 3006 | Seafood mixed dishes | 0.0 | 0.0 | 0.2 |
| 3202 | Rice mixed dishes | 0.0 | 0.2 | 0.3 |
| **3204** | **Pasta mixed dishes, excludes macaroni & cheese** | **0.0** | **0.9** | **2.2** |
| **3206** | **Macaroni & cheese** | **0.0** | **0.9** | **2.4** |
| 3208 | Turnovers & other grain-based items | 0.0 | 0.0 | 0.2 |
| 3402 | Fried rice & lo/chow mein | 0.0 | 0.0 | 0.2 |
| 3404 | Stir-fry & soy-based sauce mixtures | 0.0 | 0.1 | 0.1 |
| 3406 | Egg rolls, dumplings, sushi | 0.0 | 0.0 | 0.0 |
| 3502 | Burritos & tacos | 0.0 | 0.0 | 0.3 |
| 3504 | Nachos | 0.0 | 0.0 | 0.0 |
| 3506 | Other Mexican mixed dishes | 0.0 | 0.1 | 0.6 |
| **3602** | **Pizza** | **0.0** | **0.1** | **1.9** |
| 3702 | Burgers (single code) | 0.0 | 0.1 | 0.4 |
| 3704 | Chicken/turkey sandwiches (single code) | 0.0 | 0.0 | 0.1 |
| 3706 | Egg/breakfast sandwiches (single code) | 0.0 | 0.0 | 0.1 |
| 3708 | Other sandwiches (single code) | 0.0 | 0.0 | 0.7 |
| **3802** | **Soups** | **0.0** | **0.8** | **1.5** |
| 4002 | Rice | 0.0 | 0.0 | 0.2 |
| 4004 | Pasta, noodles, cooked grains | 0.0 | 0.0 | 0.1 |
| **4202** | **Yeast breads** | **0.0** | **0.3** | **1.0** |
| 4204 | Rolls & buns | 0.0 | 0.0 | 0.5 |
| 4206 | Bagels & English muffins | 0.0 | 0.0 | 0.0 |
| 4208 | Tortillas | 0.0 | 0.1 | 0.2 |
| 4402 | Biscuits, muffins, quick breads | 0.0 | 0.2 | 0.7 |
| 4404 | Pancakes, waffles, French toast | 0.0 | 0.1 | 0.8 |
| 4602 | Ready-to-eat cereal, higher sugar (>21.2g/100g) | 0.0 | 0.0 | 0.3 |
| 4604 | Ready-to-eat cereal, lower sugar (≤21.2g/100g) | 0.0 | 0.2 | 0.4 |
| 4802 | Oatmeal | 0.0 | 0.1 | 0.6 |
| 4804 | Grits & other cooked cereals | 0.0 | 0.1 | 0.2 |
| **5002** | **Potato chips** | **0.0** | **0.0** | **1.0** |
| **5004** | **Tortilla, corn, other chips** | **0.0** | **0.4** | **1.8** |
| 5006 | Popcorn | 0.0 | 0.0 | 0.3 |
| 5008 | Pretzels/snack mix | 0.0 | 0.0 | 0.1 |
| **5202** | **Crackers, excludes saltines** | **0.0** | **0.6** | **2.7** |
| 5204 | Saltine crackers | 0.0 | 0.1 | 0.2 |
| 5402 | Cereal bars | 0.0 | 0.0 | 0.4 |
| 5404 | Nutrition bars | 0.0 | 0.0 | 0.0 |
| 5502 | Cakes & pies | 0.0 | 0.2 | 0.8 |
| **5504** | **Cookies & brownies** | **0.0** | **1.2** | **2.9** |
| 5506 | Doughnuts, sweet rolls, pastries | 0.0 | 0.2 | 0.9 |
| 5702 | Candy containing chocolate | 0.0 | 0.0 | 0.4 |
| 5704 | Candy not containing chocolate | 0.0 | 0.0 | 0.2 |
| 5802 | Ice cream & frozen dairy desserts | 0.0 | 0.2 | 0.9 |
| 5804 | Pudding | 0.0 | 0.1 | 0.2 |
| 5806 | Gelatins, ices, sorbets | 0.0 | 0.0 | 0.0 |
| 6002 | Apples | 0.0 | 0.0 | 0.1 |
| 6004 | Bananas | 0.0 | 0.1 | 0.2 |
| 6006 | Grapes | 0.0 | 0.0 | 0.0 |
| 6008 | Peaches & nectarines | 0.0 | 0.0 | 0.0 |
| 6010 | Berries | 0.0 | 0.0 | 0.0 |
| 6012 | Citrus fruits | 0.0 | 0.0 | 0.0 |
| 6014 | Melons | 0.0 | 0.0 | 0.0 |
| 6016 | Dried fruits | 0.0 | 0.0 | 0.0 |
| 6018 | Other fruits & fruit salads | 0.0 | 0.0 | 0.0 |
| 6402 | Tomatoes | 0.0 | 0.0 | 0.0 |
| 6404 | Carrots | 0.0 | 0.0 | 0.0 |
| 6406 | Other red & orange vegetables | 0.0 | 0.0 | 0.0 |
| 6408 | Dark green vegetables, excludes lettuce | 0.0 | 0.0 | 0.0 |
| 6410 | Lettuce & lettuce salads | 0.0 | 0.0 | 0.0 |
| 6412 | String beans | 0.0 | 0.0 | 0.1 |
| 6414 | Onions | 0.0 | 0.0 | 0.0 |
| 6416 | Corn | 0.0 | 0.0 | 0.1 |
| 6418 | Other starchy vegetables | 0.0 | 0.0 | 0.1 |
| 6420 | Other vegetables & combinations | 0.0 | 0.1 | 0.2 |
| 6422 | Vegetable mixed dishes | 0.0 | 0.0 | 0.3 |
| 6802 | White potatoes, baked or boiled | 0.0 | 0.0 | 0.1 |
| **6804** | **French fries & other fried white potatoes** | **0.0** | **0.4** | **1.9** |
| 6806 | Mashed potatoes & white potato mixtures | 0.0 | 0.4 | 0.4 |
| 7002 | Citrus juice | 0.0 | 0.0 | 0.1 |
| 7004 | Apple juice | 0.0 | 0.0 | 0.2 |
| 7006 | Other fruit juice | 0.0 | 0.0 | 0.1 |
| 7008 | Vegetable juice | 0.0 | 0.0 | 0.0 |
| 7102 | Diet soft drinks | 0.0 | 0.0 | 0.0 |
| 7106 | Other diet drinks | 0.0 | 0.0 | 0.0 |
| 7202 | Soft drinks | 0.0 | 0.0 | 0.0 |
| 7204 | Fruit drinks | 0.0 | 0.0 | 0.1 |
| 7206 | Sport & energy drinks | 0.0 | 0.0 | 0.0 |
| 7208 | Nutritional beverages | 0.0 | 0.0 | 0.1 |
| 7302 | Coffee | 0.0 | 0.0 | 0.0 |
| 7304 | Tea | 0.0 | 0.0 | 0.0 |
| 7702 | Tap water | 0.0 | 0.0 | 0.0 |
| 7704 | Bottled water | 0.0 | 0.0 | 0.0 |
| 7802 | Flavored or carbonated water | 0.0 | 0.0 | 0.0 |
| 7804 | Enhanced or fortified water | 0.0 | 0.0 | 0.0 |
| 8002 | Butter & animal fats | 0.0 | 0.2 | 0.7 |
| 8004 | Margarine | 0.0 | 0.1 | 0.5 |
| 8006 | Cream cheese, sour cream, whipped cream | 0.0 | 0.0 | 0.2 |
| 8008 | Cream & cream substitutes | 0.0 | 0.0 | 0.1 |
| 8010 | Mayonnaise | 0.0 | 0.1 | 0.1 |
| 8012 | Salad dressings & vegetable oils | 0.0 | 0.0 | 0.4 |
| 8402 | Tomato-based condiments | 0.0 | 0.0 | 0.0 |
| 8404 | Soy-based condiments | 0.0 | 0.0 | 0.0 |
| 8406 | Mustard & other condiments | 0.0 | 0.0 | 0.0 |
| 8408 | Olives, pickles, pickled vegetables | 0.0 | 0.0 | 0.0 |
| 8410 | Pasta sauces, tomato-based | 0.0 | 0.0 | 0.0 |
| 8412 | Dips, gravies, other sauces | 0.0 | 0.0 | 0.1 |
| 8802 | Sugars & honey | 0.0 | 0.0 | 0.0 |
| 8804 | Sugar substitutes | 0.0 | 0.0 | 0.0 |
| 8806 | Jams, syrups, toppings | 0.0 | 0.0 | 0.0 |
| **9002** | **Baby food: cereals** | **0.7** | **2.0** | **0.3** |
| 9004 | Baby food: fruit | 0.0 | 0.3 | 0.0 |
| 9006 | Baby food: vegetable | 0.0 | 0.3 | 0.0 |
| **9008** | **Baby food: meat & dinners** | **0.1** | **1.7** | **0.6** |
| 9010 | Baby food: yogurt | 0.0 | 0.2 | 0.1 |
| 9012 | Baby food: snacks & sweets | 0.0 | 0.5 | 0.2 |
| 9202 | Baby juice | 0.0 | 0.1 | 0.0 |
| 9204 | Baby water | 0.0 | 0.0 | 0.0 |
| **9402** | **Formula, ready-to-feed** | **2.2** | **3.4** | **0.8** |
| **9404** | **Formula, prepared from powder** | **57.0** | **52.6** | **2.0** |
| **9406** | **Formula, prepared from concentrate** | **5.2** | **4.4** | **0.1** |
| **9602** | **Human milk** | **34.7** | **14.8** | **2.2** |
| 9999 | Not included in a food category | 0.0 | 0.0 | 0.0 |
|  | Total | 100 | 100 | 100 |

^a^ Contributions from all minor WWEIA food categories are reported. Those in bold contribute ≥1% to daily intake in at least one age group.

**Table S4.** Food sources of saturated fat among U.S. infants and toddlers aged 0–23.9 months by age group: NHANES 2005-12 (*n* = 2740) ^a^.

| **WWEIA Minor Food category code** | **Food category** | **% of daily intake** | | |
| --- | --- | --- | --- | --- |
|  |  | **0–5.9 mo (*n* = 765)** | **6–11.9 mo (*n* = 854)** | **12–23.9 mo  (*n* = 1121)** |
| **1002** | **Milk, whole** | **0.0** | **4.6** | **36.4** |
| **1004** | **Milk, reduced fat** | **0.0** | **0.6** | **6.1** |
| 1006 | Milk, low-fat | 0.0 | 0.2 | 0.3 |
| 1008 | Milk, non-fat | 0.0 | 0.0 | 0.0 |
| **1202** | **Flavored milk, whole** | **0.0** | **0.0** | **1.1** |
| 1204 | Flavored milk, reduced fat | 0.0 | 0.0 | 0.3 |
| 1206 | Flavored milk, low-fat | 0.0 | 0.0 | 0.1 |
| 1208 | Flavored milk, non-fat | 0.0 | 0.0 | 0.0 |
| 1402 | Milk shakes & other dairy drinks | 0.0 | 0.0 | 0.0 |
| 1404 | Milk substitutes | 0.0 | 0.0 | 0.4 |
| **1602** | **Cheese** | **0.0** | **1.9** | **7.3** |
| 1604 | Cottage/ricotta cheese | 0.0 | 0.0 | 0.1 |
| 1802 | Yogurt, whole & reduced fat | 0.0 | 0.6 | 0.9 |
| 1804 | Yogurt, low-fat & non-fat | 0.0 | 0.2 | 0.5 |
| 2002 | Beef, excludes ground | 0.0 | 0.1 | 0.3 |
| 2004 | Ground beef | 0.0 | 0.2 | 0.6 |
| 2006 | Pork | 0.0 | 0.0 | 0.1 |
| 2008 | Lamb, goat, game | 0.0 | 0.0 | 0.0 |
| 2010 | Liver & organ meats | 0.0 | 0.0 | 0.0 |
| **2202** | **Chicken, whole pieces** | **0.0** | **0.1** | **1.0** |
| **2204** | **Chicken patties, nuggets & tenders** | **0.0** | **0.1** | **1.6** |
| 2206 | Turkey, duck, other poultry | 0.0 | 0.1 | 0.1 |
| 2402 | Fish | 0.0 | 0.0 | 0.1 |
| 2404 | Shellfish | 0.0 | 0.0 | 0.0 |
| **2502** | **Eggs & omelets** | **0.0** | **1.0** | **3.5** |
| 2602 | Cold cuts & cured meats | 0.0 | 0.1 | 0.5 |
| 2604 | Bacon | 0.0 | 0.1 | 0.2 |
| **2606** | **Frankfurters** | **0.0** | **0.7** | **2.6** |
| **2608** | **Sausages** | **0.0** | **0.5** | **1.2** |
| 2802 | Beans, peas, legumes | 0.0 | 0.2 | 0.2 |
| 2804 | Nuts & seeds | 0.0 | 0.0 | 0.8 |
| 2806 | Processed soy products | 0.0 | 0.1 | 0.0 |
| 3002 | Meat mixed dishes | 0.0 | 0.3 | 0.8 |
| 3004 | Poultry mixed dishes | 0.0 | 0.1 | 0.8 |
| 3006 | Seafood mixed dishes | 0.0 | 0.0 | 0.1 |
| 3202 | Rice mixed dishes | 0.0 | 0.1 | 0.1 |
| **3204** | **Pasta mixed dishes, excludes macaroni & cheese** | **0.0** | **0.7** | **1.7** |
| **3206** | **Macaroni & cheese** | **0.0** | **0.9** | **2.5** |
| 3208 | Turnovers & other grain-based items | 0.0 | 0.0 | 0.1 |
| 3402 | Fried rice & lo/chow mein | 0.0 | 0.0 | 0.1 |
| 3404 | Stir-fry & soy-based sauce mixtures | 0.0 | 0.0 | 0.1 |
| 3406 | Egg rolls, dumplings, sushi | 0.0 | 0.0 | 0.0 |
| 3502 | Burritos & tacos | 0.0 | 0.0 | 0.3 |
| 3504 | Nachos | 0.0 | 0.0 | 0.0 |
| 3506 | Other Mexican mixed dishes | 0.0 | 0.1 | 0.6 |
| **3602** | **Pizza** | **0.0** | **0.1** | **1.8** |
| 3702 | Burgers (single code) | 0.0 | 0.2 | 0.4 |
| 3704 | Chicken/turkey sandwiches (single code) | 0.0 | 0.0 | 0.1 |
| 3706 | Egg/breakfast sandwiches (single code) | 0.0 | 0.0 | 0.0 |
| 3708 | Other sandwiches (single code) | 0.0 | 0.0 | 0.5 |
| **3802** | **Soups** | **0.0** | **0.6** | **1.1** |
| 4002 | Rice | 0.0 | 0.0 | 0.1 |
| 4004 | Pasta, noodles, cooked grains | 0.0 | 0.0 | 0.0 |
| 4202 | Yeast breads | 0.0 | 0.1 | 0.6 |
| 4204 | Rolls & buns | 0.0 | 0.0 | 0.2 |
| 4206 | Bagels & English muffins | 0.0 | 0.0 | 0.0 |
| 4208 | Tortillas | 0.0 | 0.1 | 0.1 |
| 4402 | Biscuits, muffins, quick breads | 0.0 | 0.1 | 0.3 |
| 4404 | Pancakes, waffles, French toast | 0.0 | 0.1 | 0.4 |
| 4602 | Ready-to-eat cereal, higher sugar (>21.2g/100g) | 0.0 | 0.0 | 0.2 |
| 4604 | Ready-to-eat cereal, lower sugar (≤21.2g/100g) | 0.0 | 0.1 | 0.2 |
| 4802 | Oatmeal | 0.0 | 0.1 | 0.5 |
| 4804 | Grits & other cooked cereals | 0.0 | 0.1 | 0.2 |
| 5002 | Potato chips | 0.0 | 0.0 | 0.4 |
| 5004 | Tortilla, corn, other chips | 0.0 | 0.2 | 0.6 |
| 5006 | Popcorn | 0.0 | 0.0 | 0.1 |
| 5008 | Pretzels/snack mix | 0.0 | 0.0 | 0.1 |
| **5202** | **Crackers, excludes saltines** | **0.0** | **0.3** | **1.4** |
| 5204 | Saltine crackers | 0.0 | 0.0 | 0.1 |
| 5402 | Cereal bars | 0.0 | 0.0 | 0.4 |
| 5404 | Nutrition bars | 0.0 | 0.0 | 0.0 |
| 5502 | Cakes & pies | 0.0 | 0.1 | 0.5 |
| **5504** | **Cookies & brownies** | **0.0** | **0.7** | **2.0** |
| 5506 | Doughnuts, sweet rolls, pastries | 0.0 | 0.2 | 0.6 |
| 5702 | Candy containing chocolate | 0.0 | 0.0 | 0.6 |
| 5704 | Candy not containing chocolate | 0.0 | 0.1 | 0.2 |
| **5802** | **Ice cream & frozen dairy desserts** | **0.0** | **0.2** | **1.3** |
| 5804 | Pudding | 0.0 | 0.1 | 0.1 |
| 5806 | Gelatins, ices, sorbets | 0.0 | 0.0 | 0.0 |
| 6002 | Apples | 0.0 | 0.0 | 0.0 |
| 6004 | Bananas | 0.0 | 0.1 | 0.1 |
| 6006 | Grapes | 0.0 | 0.0 | 0.0 |
| 6008 | Peaches & nectarines | 0.0 | 0.0 | 0.0 |
| 6010 | Berries | 0.0 | 0.0 | 0.0 |
| 6012 | Citrus fruits | 0.0 | 0.0 | 0.0 |
| 6014 | Melons | 0.0 | 0.0 | 0.0 |
| 6016 | Dried fruits | 0.0 | 0.0 | 0.0 |
| 6018 | Other fruits & fruit salads | 0.0 | 0.0 | 0.0 |
| 6402 | Tomatoes | 0.0 | 0.0 | 0.0 |
| 6404 | Carrots | 0.0 | 0.0 | 0.0 |
| 6406 | Other red & orange vegetables | 0.0 | 0.0 | 0.0 |
| 6408 | Dark green vegetables, excludes lettuce | 0.0 | 0.0 | 0.0 |
| 6410 | Lettuce & lettuce salads | 0.0 | 0.0 | 0.0 |
| 6412 | String beans | 0.0 | 0.0 | 0.1 |
| 6414 | Onions | 0.0 | 0.0 | 0.0 |
| 6416 | Corn | 0.0 | 0.0 | 0.1 |
| 6418 | Other starchy vegetables | 0.0 | 0.0 | 0.1 |
| 6420 | Other vegetables & combinations | 0.0 | 0.1 | 0.1 |
| 6422 | Vegetable mixed dishes | 0.0 | 0.0 | 0.2 |
| 6802 | White potatoes, baked or boiled | 0.0 | 0.0 | 0.0 |
| **6804** | **French fries & other fried white potatoes** | **0.0** | **0.2** | **1.0** |
| 6806 | Mashed potatoes & white potato mixtures | 0.0 | 0.4 | 0.5 |
| 7002 | Citrus juice | 0.0 | 0.0 | 0.0 |
| 7004 | Apple juice | 0.0 | 0.0 | 0.1 |
| 7006 | Other fruit juice | 0.0 | 0.0 | 0.1 |
| 7008 | Vegetable juice | 0.0 | 0.0 | 0.0 |
| 7102 | Diet soft drinks | 0.0 | 0.0 | 0.0 |
| 7106 | Other diet drinks | 0.0 | 0.0 | 0.0 |
| 7202 | Soft drinks | 0.0 | 0.0 | 0.0 |
| 7204 | Fruit drinks | 0.0 | 0.0 | 0.1 |
| 7206 | Sport & energy drinks | 0.0 | 0.0 | 0.0 |
| 7208 | Nutritional beverages | 0.0 | 0.0 | 0.0 |
| 7302 | Coffee | 0.0 | 0.0 | 0.0 |
| 7304 | Tea | 0.0 | 0.0 | 0.0 |
| 7702 | Tap water | 0.0 | 0.0 | 0.0 |
| 7704 | Bottled water | 0.0 | 0.0 | 0.0 |
| 7802 | Flavored or carbonated water | 0.0 | 0.0 | 0.0 |
| 7804 | Enhanced or fortified water | 0.0 | 0.0 | 0.0 |
| **8002** | **Butter & animal fats** | **0.0** | **0.3** | **1.0** |
| 8004 | Margarine | 0.0 | 0.1 | 0.2 |
| 8006 | Cream cheese, sour cream, whipped cream | 0.0 | 0.0 | 0.2 |
| 8008 | Cream & cream substitutes | 0.0 | 0.0 | 0.2 |
| 8010 | Mayonnaise | 0.0 | 0.0 | 0.0 |
| 8012 | Salad dressings & vegetable oils | 0.0 | 0.0 | 0.1 |
| 8402 | Tomato-based condiments | 0.0 | 0.0 | 0.0 |
| 8404 | Soy-based condiments | 0.0 | 0.0 | 0.0 |
| 8406 | Mustard & other condiments | 0.0 | 0.0 | 0.0 |
| 8408 | Olives, pickles, pickled vegetables | 0.0 | 0.0 | 0.0 |
| 8410 | Pasta sauces, tomato-based | 0.0 | 0.0 | 0.0 |
| 8412 | Dips, gravies, other sauces | 0.0 | 0.0 | 0.1 |
| 8802 | Sugars & honey | 0.0 | 0.0 | 0.0 |
| 8804 | Sugar substitutes | 0.0 | 0.0 | 0.0 |
| 8806 | Jams, syrups, toppings | 0.0 | 0.0 | 0.0 |
| 9002 | Baby food: cereals | 0.3 | 0.9 | 0.1 |
| 9004 | Baby food: fruit | 0.0 | 0.2 | 0.0 |
| 9006 | Baby food: vegetable | 0.0 | 0.1 | 0.0 |
| **9008** | **Baby food: meat & dinners** | **0.1** | **1.3** | **0.5** |
| 9010 | Baby food: yogurt | 0.0 | 0.3 | 0.1 |
| 9012 | Baby food: snacks & sweets | 0.0 | 0.2 | 0.1 |
| 9202 | Baby juice | 0.0 | 0.0 | 0.0 |
| 9204 | Baby water | 0.0 | 0.0 | 0.0 |
| **9402** | **Formula, ready-to-feed** | **2.0** | **3.6** | **0.7** |
| **9404** | **Formula, prepared from powder** | **54.8** | **53.7** | **2.0** |
| **9406** | **Formula, prepared from concentrate** | **5.0** | **4.5** | **0.1** |
| **9602** | **Human milk** | **37.7** | **16.6** | **2.5** |
| 9999 | Not included in a food category | 0.0 | 0.0 | 0.0 |
|  | Total | 100 | 100 | 100 |

^a^ Contributions from all minor WWEIA food categories are reported. Those in bold contribute ≥1% to daily intake in at least one age group.

**Table S5.** Food sources of total carbohydrate among U.S. infants and toddlers aged 0–23.9 months by age group: NHANES 2005-12 (*n* = 2740) ^a^.

| **WWEIA Minor Food category code** | **Food category** | **% of daily intake** | | |
| --- | --- | --- | --- | --- |
|  |  | **0–5.9 mo (*n* = 765)** | **6–11.9 mo (*n* = 854)** | **12–23.9 mo  (*n* = 1121)** |
| **1002** | **Milk, whole** | **0.0** | **1.5** | **10.8** |
| **1004** | **Milk, reduced fat** | **0.0** | **0.3** | **2.7** |
| 1006 | Milk, low-fat | 0.0 | 0.2 | 0.3 |
| 1008 | Milk, non-fat | 0.0 | 0.0 | 0.3 |
| 1202 | Flavored milk, whole | 0.0 | 0.0 | 0.8 |
| 1204 | Flavored milk, reduced fat | 0.0 | 0.0 | 0.4 |
| 1206 | Flavored milk, low-fat | 0.0 | 0.0 | 0.2 |
| 1208 | Flavored milk, non-fat | 0.0 | 0.0 | 0.0 |
| 1402 | Milk shakes & other dairy drinks | 0.0 | 0.0 | 0.0 |
| 1404 | Milk substitutes | 0.0 | 0.2 | 0.9 |
| 1602 | Cheese | 0.0 | 0.1 | 0.3 |
| 1604 | Cottage/ricotta cheese | 0.0 | 0.0 | 0.0 |
| 1802 | Yogurt, whole & reduced fat | 0.0 | 0.5 | 0.9 |
| **1804** | **Yogurt, low-fat & non-fat** | **0.0** | **0.6** | **1.3** |
| 2002 | Beef, excludes ground | 0.0 | 0.0 | 0.0 |
| 2004 | Ground beef | 0.0 | 0.0 | 0.0 |
| 2006 | Pork | 0.0 | 0.0 | 0.0 |
| 2008 | Lamb, goat, game | 0.0 | 0.0 | 0.0 |
| 2010 | Liver & organ meats | 0.0 | 0.0 | 0.0 |
| 2202 | Chicken, whole pieces | 0.0 | 0.0 | 0.2 |
| 2204 | Chicken patties, nuggets & tenders | 0.0 | 0.1 | 0.8 |
| 2206 | Turkey, duck, other poultry | 0.0 | 0.0 | 0.0 |
| 2402 | Fish | 0.0 | 0.0 | 0.1 |
| 2404 | Shellfish | 0.0 | 0.0 | 0.0 |
| 2502 | Eggs & omelets | 0.0 | 0.1 | 0.2 |
| 2602 | Cold cuts & cured meats | 0.0 | 0.0 | 0.1 |
| 2604 | Bacon | 0.0 | 0.0 | 0.0 |
| 2606 | Frankfurters | 0.0 | 0.0 | 0.1 |
| 2608 | Sausages | 0.0 | 0.0 | 0.0 |
| 2802 | Beans, peas, legumes | 0.0 | 0.7 | 0.8 |
| 2804 | Nuts & seeds | 0.0 | 0.0 | 0.2 |
| 2806 | Processed soy products | 0.0 | 0.1 | 0.0 |
| 3002 | Meat mixed dishes | 0.0 | 0.2 | 0.5 |
| 3004 | Poultry mixed dishes | 0.0 | 0.1 | 0.6 |
| 3006 | Seafood mixed dishes | 0.0 | 0.0 | 0.1 |
| 3202 | Rice mixed dishes | 0.0 | 0.3 | 0.7 |
| **3204** | **Pasta mixed dishes, excludes macaroni & cheese** | **0.0** | **1.1** | **2.7** |
| **3206** | **Macaroni & cheese** | **0.0** | **0.8** | **2.1** |
| 3208 | Turnovers & other grain-based items | 0.0 | 0.0 | 0.1 |
| 3402 | Fried rice & lo/chow mein | 0.0 | 0.0 | 0.2 |
| 3404 | Stir-fry & soy-based sauce mixtures | 0.0 | 0.0 | 0.0 |
| 3406 | Egg rolls, dumplings, sushi | 0.0 | 0.0 | 0.0 |
| 3502 | Burritos & tacos | 0.0 | 0.0 | 0.2 |
| 3504 | Nachos | 0.0 | 0.0 | 0.0 |
| 3506 | Other Mexican mixed dishes | 0.0 | 0.0 | 0.3 |
| **3602** | **Pizza** | **0.0** | **0.1** | **1.1** |
| 3702 | Burgers (single code) | 0.0 | 0.1 | 0.2 |
| 3704 | Chicken/turkey sandwiches (single code) | 0.0 | 0.0 | 0.1 |
| 3706 | Egg/breakfast sandwiches (single code) | 0.0 | 0.0 | 0.0 |
| 3708 | Other sandwiches (single code) | 0.0 | 0.0 | 0.3 |
| **3802** | **Soups** | **0.0** | **0.9** | **1.3** |
| **4002** | **Rice** | **0.0** | **0.6** | **1.0** |
| 4004 | Pasta, noodles, cooked grains | 0.0 | 0.1 | 0.3 |
| **4202** | **Yeast breads** | **0.0** | **1.0** | **3.5** |
| 4204 | Rolls & buns | 0.0 | 0.1 | 0.8 |
| 4206 | Bagels & English muffins | 0.0 | 0.0 | 0.3 |
| 4208 | Tortillas | 0.0 | 0.3 | 0.6 |
| 4402 | Biscuits, muffins, quick breads | 0.0 | 0.2 | 0.7 |
| **4404** | **Pancakes, waffles, French toast** | **0.0** | **0.2** | **1.2** |
| **4602** | **Ready-to-eat cereal, higher sugar (>21.2g/100g)** | **0.0** | **0.3** | **1.9** |
| **4604** | **Ready-to-eat cereal, lower sugar (≤21.2g/100g)** | **0.0** | **0.8** | **1.9** |
| **4802** | **Oatmeal** | **0.0** | **0.4** | **1.5** |
| 4804 | Grits & other cooked cereals | 0.0 | 0.2 | 0.4 |
| 5002 | Potato chips | 0.0 | 0.0 | 0.4 |
| **5004** | **Tortilla, corn, other chips** | **0.0** | **0.3** | **1.0** |
| 5006 | Popcorn | 0.0 | 0.0 | 0.1 |
| 5008 | Pretzels/snack mix | 0.0 | 0.1 | 0.8 |
| **5202** | **Crackers, excludes saltines** | **0.0** | **0.6** | **2.1** |
| 5204 | Saltine crackers | 0.0 | 0.2 | 0.4 |
| 5402 | Cereal bars | 0.0 | 0.1 | 0.8 |
| 5404 | Nutrition bars | 0.0 | 0.0 | 0.0 |
| 5502 | Cakes & pies | 0.0 | 0.2 | 0.8 |
| **5504** | **Cookies & brownies** | **0.0** | **1.8** | **3.6** |
| **5506** | **Doughnuts, sweet rolls, pastries** | **0.0** | **0.2** | **1.0** |
| 5702 | Candy containing chocolate | 0.0 | 0.0 | 0.3 |
| **5704** | **Candy not containing chocolate** | **0.0** | **0.2** | **1.8** |
| 5802 | Ice cream & frozen dairy desserts | 0.0 | 0.2 | 0.8 |
| 5804 | Pudding | 0.0 | 0.2 | 0.3 |
| 5806 | Gelatins, ices, sorbets | 0.0 | 0.1 | 0.4 |
| **6002** | **Apples** | **0.0** | **1.0** | **1.8** |
| **6004** | **Bananas** | **0.1** | **2.1** | **3.2** |
| 6006 | Grapes | 0.0 | 0.1 | 0.7 |
| 6008 | Peaches & nectarines | 0.0 | 0.3 | 0.5 |
| 6010 | Berries | 0.0 | 0.1 | 0.3 |
| 6012 | Citrus fruits | 0.0 | 0.1 | 0.6 |
| 6014 | Melons | 0.0 | 0.1 | 0.3 |
| 6016 | Dried fruits | 0.0 | 0.3 | 0.7 |
| **6018** | **Other fruits & fruit salads** | **0.0** | **0.4** | **1.4** |
| 6402 | Tomatoes | 0.0 | 0.0 | 0.1 |
| 6404 | Carrots | 0.0 | 0.1 | 0.1 |
| 6406 | Other red & orange vegetables | 0.0 | 0.2 | 0.1 |
| 6408 | Dark green vegetables, excludes lettuce | 0.0 | 0.0 | 0.1 |
| 6410 | Lettuce & lettuce salads | 0.0 | 0.0 | 0.0 |
| 6412 | String beans | 0.0 | 0.1 | 0.2 |
| 6414 | Onions | 0.0 | 0.0 | 0.0 |
| 6416 | Corn | 0.0 | 0.1 | 0.4 |
| 6418 | Other starchy vegetables | 0.0 | 0.2 | 0.2 |
| 6420 | Other vegetables & combinations | 0.0 | 0.2 | 0.2 |
| 6422 | Vegetable mixed dishes | 0.0 | 0.0 | 0.1 |
| 6802 | White potatoes, baked or boiled | 0.0 | 0.0 | 0.2 |
| **6804** | **French fries & other fried white potatoes** | **0.0** | **0.3** | **1.2** |
| 6806 | Mashed potatoes & white potato mixtures | 0.1 | 0.5 | 0.6 |
| **7002** | **Citrus juice** | **0.0** | **0.2** | **1.4** |
| **7004** | **Apple juice** | **0.1** | **1.3** | **5.2** |
| **7006** | **Other fruit juice** | **0.2** | **1.3** | **4.5** |
| 7008 | Vegetable juice | 0.0 | 0.0 | 0.0 |
| 7102 | Diet soft drinks | 0.0 | 0.0 | 0.0 |
| 7106 | Other diet drinks | 0.0 | 0.0 | 0.0 |
| 7202 | Soft drinks | 0.0 | 0.1 | 0.7 |
| **7204** | **Fruit drinks** | **0.0** | **0.5** | **4.5** |
| 7206 | Sport & energy drinks | 0.1 | 0.1 | 0.4 |
| 7208 | Nutritional beverages | 0.0 | 0.0 | 0.2 |
| 7302 | Coffee | 0.0 | 0.0 | 0.0 |
| 7304 | Tea | 0.0 | 0.1 | 0.3 |
| 7702 | Tap water | 0.0 | 0.0 | 0.0 |
| 7704 | Bottled water | 0.0 | 0.0 | 0.0 |
| 7802 | Flavored or carbonated water | 0.0 | 0.0 | 0.0 |
| 7804 | Enhanced or fortified water | 0.0 | 0.0 | 0.0 |
| 8002 | Butter & animal fats | 0.0 | 0.0 | 0.0 |
| 8004 | Margarine | 0.0 | 0.0 | 0.0 |
| 8006 | Cream cheese, sour cream, whipped cream | 0.0 | 0.0 | 0.0 |
| 8008 | Cream & cream substitutes | 0.0 | 0.0 | 0.0 |
| 8010 | Mayonnaise | 0.0 | 0.0 | 0.0 |
| 8012 | Salad dressings & vegetable oils | 0.0 | 0.0 | 0.0 |
| 8402 | Tomato-based condiments | 0.0 | 0.0 | 0.1 |
| 8404 | Soy-based condiments | 0.0 | 0.0 | 0.0 |
| 8406 | Mustard & other condiments | 0.0 | 0.0 | 0.0 |
| 8408 | Olives, pickles, pickled vegetables | 0.0 | 0.0 | 0.0 |
| 8410 | Pasta sauces, tomato-based | 0.0 | 0.0 | 0.0 |
| 8412 | Dips, gravies, other sauces | 0.0 | 0.0 | 0.0 |
| 8802 | Sugars & honey | 0.0 | 0.0 | 0.2 |
| 8804 | Sugar substitutes | 0.0 | 0.0 | 0.0 |
| 8806 | Jams, syrups, toppings | 0.0 | 0.1 | 0.8 |
| **9002** | **Baby food: cereals** | **4.6** | **9.1** | **1.2** |
| **9004** | **Baby food: fruit** | **1.2** | **7.3** | **0.7** |
| **9006** | **Baby food: vegetable** | **0.9** | **3.4** | **0.3** |
| **9008** | **Baby food: meat & dinners** | **0.1** | **2.4** | **0.8** |
| 9010 | Baby food: yogurt | 0.0 | 0.9 | 0.2 |
| **9012** | **Baby food: snacks & sweets** | **0.1** | **1.9** | **0.7** |
| **9202** | **Baby juice** | **0.7** | **2.8** | **0.8** |
| 9204 | Baby water | 0.0 | 0.0 | 0.0 |
| **9402** | **Formula, ready-to-feed** | **2.2** | **2.1** | **0.5** |
| **9404** | **Formula, prepared from powder** | **57.0** | **33.1** | **1.2** |
| **9406** | **Formula, prepared from concentrate** | **5.2** | **2.8** | **0.0** |
| **9602** | **Human milk** | **27.2** | **7.3** | **1.0** |
| 9999 | Not included in a food category | 0.0 | 0.0 | 0.2 |
|  | Total | 100 | 100 | 100 |

^a^ Contributions from all minor WWEIA food categories are reported. Those in bold contribute ≥1% to daily intake in at least one age group.

**Table S6.** Food sources of total sugars among U.S. infants and toddlers aged 0–23.9 months by age group: NHANES 2005-12 (*n* = 2740) ^a^.

| **WWEIA Minor Food category code** | **Food category** | **% of daily intake** | | |
| --- | --- | --- | --- | --- |
|  |  | **0–5.9 mo (*n* = 765)** | **6–11.9 mo (*n* = 854)** | **12–23.9 mo  (*n* = 1121)** |
| **1002** | **Milk, whole** | **0.0** | **2.4** | **20.3** |
| **1004** | **Milk, reduced fat** | **0.0** | **0.5** | **5.0** |
| 1006 | Milk, low-fat | 0.0 | 0.3 | 0.5 |
| 1008 | Milk, non-fat | 0.0 | 0.0 | 0.5 |
| **1202** | **Flavored milk, whole** | **0.0** | **0.1** | **1.3** |
| 1204 | Flavored milk, reduced fat | 0.0 | 0.0 | 0.6 |
| 1206 | Flavored milk, low-fat | 0.0 | 0.0 | 0.4 |
| 1208 | Flavored milk, non-fat | 0.0 | 0.0 | 0.0 |
| 1402 | Milk shakes & other dairy drinks | 0.0 | 0.0 | 0.0 |
| 1404 | Milk substitutes | 0.0 | 0.3 | 0.7 |
| 1602 | Cheese | 0.0 | 0.1 | 0.3 |
| 1604 | Cottage/ricotta cheese | 0.0 | 0.0 | 0.0 |
| **1802** | **Yogurt, whole & reduced fat** | **0.0** | **0.8** | **1.5** |
| **1804** | **Yogurt, low-fat & non-fat** | **0.0** | **0.8** | **2.0** |
| 2002 | Beef, excludes ground | 0.0 | 0.0 | 0.0 |
| 2004 | Ground beef | 0.0 | 0.0 | 0.0 |
| 2006 | Pork | 0.0 | 0.0 | 0.0 |
| 2008 | Lamb, goat, game | 0.0 | 0.0 | 0.0 |
| 2010 | Liver & organ meats | 0.0 | 0.0 | 0.0 |
| 2202 | Chicken, whole pieces | 0.0 | 0.0 | 0.1 |
| 2204 | Chicken patties, nuggets & tenders | 0.0 | 0.0 | 0.0 |
| 2206 | Turkey, duck, other poultry | 0.0 | 0.0 | 0.0 |
| 2402 | Fish | 0.0 | 0.0 | 0.0 |
| 2404 | Shellfish | 0.0 | 0.0 | 0.0 |
| 2502 | Eggs & omelets | 0.0 | 0.1 | 0.3 |
| 2602 | Cold cuts & cured meats | 0.0 | 0.0 | 0.1 |
| 2604 | Bacon | 0.0 | 0.0 | 0.0 |
| 2606 | Frankfurters | 0.0 | 0.0 | 0.1 |
| 2608 | Sausages | 0.0 | 0.0 | 0.0 |
| 2802 | Beans, peas, legumes | 0.0 | 0.2 | 0.2 |
| 2804 | Nuts & seeds | 0.0 | 0.0 | 0.2 |
| 2806 | Processed soy products | 0.0 | 0.0 | 0.0 |
| 3002 | Meat mixed dishes | 0.0 | 0.0 | 0.2 |
| 3004 | Poultry mixed dishes | 0.0 | 0.0 | 0.2 |
| 3006 | Seafood mixed dishes | 0.0 | 0.0 | 0.0 |
| 3202 | Rice mixed dishes | 0.0 | 0.0 | 0.1 |
| 3204 | Pasta mixed dishes, excludes macaroni & cheese | 0.0 | 0.3 | 0.8 |
| 3206 | Macaroni & cheese | 0.0 | 0.1 | 0.5 |
| 3208 | Turnovers & other grain-based items | 0.0 | 0.0 | 0.0 |
| 3402 | Fried rice & lo/chow mein | 0.0 | 0.0 | 0.0 |
| 3404 | Stir-fry & soy-based sauce mixtures | 0.0 | 0.0 | 0.0 |
| 3406 | Egg rolls, dumplings, sushi | 0.0 | 0.0 | 0.0 |
| 3502 | Burritos & tacos | 0.0 | 0.0 | 0.0 |
| 3504 | Nachos | 0.0 | 0.0 | 0.0 |
| 3506 | Other Mexican mixed dishes | 0.0 | 0.0 | 0.1 |
| 3602 | Pizza | 0.0 | 0.0 | 0.2 |
| 3702 | Burgers (single code) | 0.0 | 0.0 | 0.1 |
| 3704 | Chicken/turkey sandwiches (single code) | 0.0 | 0.0 | 0.0 |
| 3706 | Egg/breakfast sandwiches (single code) | 0.0 | 0.0 | 0.0 |
| 3708 | Other sandwiches (single code) | 0.0 | 0.0 | 0.1 |
| 3802 | Soups | 0.0 | 0.2 | 0.4 |
| 4002 | Rice | 0.0 | 0.0 | 0.0 |
| 4004 | Pasta, noodles, cooked grains | 0.0 | 0.0 | 0.0 |
| 4202 | Yeast breads | 0.0 | 0.1 | 0.6 |
| 4204 | Rolls & buns | 0.0 | 0.0 | 0.1 |
| 4206 | Bagels & English muffins | 0.0 | 0.0 | 0.0 |
| 4208 | Tortillas | 0.0 | 0.0 | 0.0 |
| 4402 | Biscuits, muffins, quick breads | 0.0 | 0.1 | 0.3 |
| 4404 | Pancakes, waffles, French toast | 0.0 | 0.1 | 0.4 |
| **4602** | **Ready-to-eat cereal, higher sugar (>21.2g/100g)** | **0.0** | **0.2** | **1.4** |
| 4604 | Ready-to-eat cereal, lower sugar (≤21.2g/100g) | 0.0 | 0.1 | 0.4 |
| 4802 | Oatmeal | 0.0 | 0.2 | 0.8 |
| 4804 | Grits & other cooked cereals | 0.0 | 0.0 | 0.1 |
| 5002 | Potato chips | 0.0 | 0.0 | 0.0 |
| 5004 | Tortilla, corn, other chips | 0.0 | 0.0 | 0.1 |
| 5006 | Popcorn | 0.0 | 0.0 | 0.0 |
| 5008 | Pretzels/snack mix | 0.0 | 0.0 | 0.1 |
| 5202 | Crackers, excludes saltines | 0.0 | 0.1 | 0.3 |
| 5204 | Saltine crackers | 0.0 | 0.0 | 0.0 |
| 5402 | Cereal bars | 0.0 | 0.1 | 0.6 |
| 5404 | Nutrition bars | 0.0 | 0.0 | 0.0 |
| **5502** | **Cakes & pies** | **0.0** | **0.2** | **1.0** |
| **5504** | **Cookies & brownies** | **0.0** | **1.1** | **2.4** |
| 5506 | Doughnuts, sweet rolls, pastries | 0.0 | 0.1 | 0.8 |
| 5702 | Candy containing chocolate | 0.0 | 0.0 | 0.4 |
| 5704 | Candy not containing chocolate | 0.0 | 0.2 | 2.0 |
| **5802** | **Ice cream & frozen dairy desserts** | **0.0** | **0.2** | **1.1** |
| 5804 | Pudding | 0.0 | 0.2 | 0.4 |
| 5806 | Gelatins, ices, sorbets | 0.0 | 0.2 | 0.6 |
| **6002** | **Apples** | **0.0** | **1.1** | **2.5** |
| **6004** | **Bananas** | **0.0** | **1.6** | **3.0** |
| **6006** | **Grapes** | **0.0** | **0.1** | **1.1** |
| 6008 | Peaches & nectarines | 0.0 | 0.4 | 0.8 |
| 6010 | Berries | 0.0 | 0.1 | 0.4 |
| 6012 | Citrus fruits | 0.0 | 0.2 | 0.8 |
| 6014 | Melons | 0.0 | 0.1 | 0.4 |
| 6016 | Dried fruits | 0.0 | 0.3 | 0.9 |
| **6018** | **Other fruits & fruit salads** | **0.0** | **0.5** | **1.9** |
| 6402 | Tomatoes | 0.0 | 0.0 | 0.1 |
| 6404 | Carrots | 0.0 | 0.1 | 0.1 |
| 6406 | Other red & orange vegetables | 0.0 | 0.1 | 0.1 |
| 6408 | Dark green vegetables, excludes lettuce | 0.0 | 0.0 | 0.0 |
| 6410 | Lettuce & lettuce salads | 0.0 | 0.0 | 0.0 |
| 6412 | String beans | 0.0 | 0.0 | 0.1 |
| 6414 | Onions | 0.0 | 0.0 | 0.0 |
| 6416 | Corn | 0.0 | 0.0 | 0.1 |
| 6418 | Other starchy vegetables | 0.0 | 0.1 | 0.1 |
| 6420 | Other vegetables & combinations | 0.0 | 0.1 | 0.1 |
| 6422 | Vegetable mixed dishes | 0.0 | 0.0 | 0.1 |
| 6802 | White potatoes, baked or boiled | 0.0 | 0.0 | 0.0 |
| 6804 | French fries & other fried white potatoes | 0.0 | 0.0 | 0.0 |
| 6806 | Mashed potatoes & white potato mixtures | 0.0 | 0.1 | 0.1 |
| **7002** | **Citrus juice** | **0.0** | **0.2** | **1.9** |
| **7004** | **Apple juice** | **0.1** | **1.6** | **7.8** |
| **7006** | **Other fruit juice** | **0.2** | **1.8** | **6.9** |
| 7008 | Vegetable juice | 0.0 | 0.0 | 0.0 |
| 7102 | Diet soft drinks | 0.0 | 0.0 | 0.0 |
| 7106 | Other diet drinks | 0.0 | 0.0 | 0.0 |
| **7202** | **Soft drinks** | **0.0** | **0.1** | **1.1** |
| **7204** | **Fruit drinks** | **0.0** | **0.7** | **7.1** |
| 7206 | Sport & energy drinks | 0.1 | 0.2 | 0.6 |
| 7208 | Nutritional beverages | 0.0 | 0.0 | 0.2 |
| 7302 | Coffee | 0.0 | 0.0 | 0.0 |
| 7304 | Tea | 0.0 | 0.1 | 0.5 |
| 7702 | Tap water | 0.0 | 0.0 | 0.0 |
| 7704 | Bottled water | 0.0 | 0.0 | 0.0 |
| 7802 | Flavored or carbonated water | 0.0 | 0.0 | 0.1 |
| 7804 | Enhanced or fortified water | 0.0 | 0.0 | 0.1 |
| 8002 | Butter & animal fats | 0.0 | 0.0 | 0.0 |
| 8004 | Margarine | 0.0 | 0.0 | 0.0 |
| 8006 | Cream cheese, sour cream, whipped cream | 0.0 | 0.0 | 0.0 |
| 8008 | Cream & cream substitutes | 0.0 | 0.0 | 0.0 |
| 8010 | Mayonnaise | 0.0 | 0.0 | 0.0 |
| 8012 | Salad dressings & vegetable oils | 0.0 | 0.0 | 0.0 |
| 8402 | Tomato-based condiments | 0.0 | 0.0 | 0.1 |
| 8404 | Soy-based condiments | 0.0 | 0.0 | 0.0 |
| 8406 | Mustard & other condiments | 0.0 | 0.0 | 0.0 |
| 8408 | Olives, pickles, pickled vegetables | 0.0 | 0.0 | 0.0 |
| 8410 | Pasta sauces, tomato-based | 0.0 | 0.0 | 0.0 |
| 8412 | Dips, gravies, other sauces | 0.0 | 0.0 | 0.0 |
| 8802 | Sugars & honey | 0.0 | 0.0 | 0.3 |
| 8804 | Sugar substitutes | 0.0 | 0.0 | 0.0 |
| 8806 | Jams, syrups, toppings | 0.0 | 0.1 | 1.0 |
| **9002** | **Baby food: cereals** | **0.2** | **1.4** | **0.2** |
| **9004** | **Baby food: fruit** | **0.9** | **7.0** | **0.8** |
| **9006** | **Baby food: vegetable** | **0.4** | **1.8** | **0.2** |
| 9008 | Baby food: meat & dinners | 0.0 | 0.8 | 0.2 |
| 9010 | Baby food: yogurt | 0.0 | 0.9 | 0.2 |
| **9012** | **Baby food: snacks & sweets** | **0.0** | **1.3** | **0.6** |
| **9202** | **Baby juice** | **0.7** | **3.6** | **1.2** |
| 9204 | Baby water | 0.0 | 0.0 | 0.0 |
| 9402 | Formula, ready-to-feed | 2.2 | 2.8 | 0.8 |
| **9404** | **Formula, prepared from powder** | **60.0** | **46.4** | **2.0** |
| **9406** | **Formula, prepared from concentrate** | **5.5** | **3.9** | **0.1** |
| **9602** | **Human milk** | **29.6** | **10.6** | **1.7** |
| 9999 | Not included in a food category | 0.0 | 0.0 | 0.3 |
|  | Total | 100 | 100 | 100 |

^a^ Contributions from all minor WWEIA food categories are reported. Those in bold contribute ≥1% to daily intake in at least one age group.

**Table S7.** Food sources of fiber among U.S. infants and toddlers aged 0–23.9 months by age group: NHANES 2005-12 (*n* = 2740) ^a^.

| **WWEIA Minor Food category code** | **Food category** | **% of daily intake** | | |
| --- | --- | --- | --- | --- |
|  |  | **0–5.9 mo (*n* = 765)** | **6–11.9 mo (*n* = 854)** | **12–23.9 mo  (*n* = 1121)** |
| 1002 | Milk, whole | 0.0 | 0.0 | 0.0 |
| 1004 | Milk, reduced fat | 0.0 | 0.0 | 0.0 |
| 1006 | Milk, low-fat | 0.0 | 0.0 | 0.0 |
| 1008 | Milk, non-fat | 0.0 | 0.0 | 0.0 |
| 1202 | Flavored milk, whole | 0.0 | 0.0 | 0.6 |
| 1204 | Flavored milk, reduced fat | 0.0 | 0.0 | 0.3 |
| 1206 | Flavored milk, low-fat | 0.0 | 0.0 | 0.2 |
| 1208 | Flavored milk, non-fat | 0.0 | 0.0 | 0.0 |
| 1402 | Milk shakes & other dairy drinks | 0.0 | 0.0 | 0.0 |
| **1404** | **Milk substitutes** | **0.0** | **0.3** | **1.0** |
| 1602 | Cheese | 0.0 | 0.0 | 0.0 |
| 1604 | Cottage/ricotta cheese | 0.0 | 0.0 | 0.0 |
| 1802 | Yogurt, whole & reduced fat | 0.0 | 0.0 | 0.0 |
| 1804 | Yogurt, low-fat & non-fat | 0.0 | 0.0 | 0.0 |
| 2002 | Beef, excludes ground | 0.0 | 0.0 | 0.0 |
| 2004 | Ground beef | 0.0 | 0.0 | 0.0 |
| 2006 | Pork | 0.0 | 0.0 | 0.0 |
| 2008 | Lamb, goat, game | 0.0 | 0.0 | 0.0 |
| 2010 | Liver & organ meats | 0.0 | 0.0 | 0.0 |
| 2202 | Chicken, whole pieces | 0.0 | 0.0 | 0.1 |
| **2204** | **Chicken patties, nuggets & tenders** | **0.0** | **0.2** | **1.3** |
| 2206 | Turkey, duck, other poultry | 0.0 | 0.0 | 0.0 |
| 2402 | Fish | 0.0 | 0.0 | 0.1 |
| 2404 | Shellfish | 0.0 | 0.0 | 0.0 |
| 2502 | Eggs & omelets | 0.0 | 0.0 | 0.1 |
| 2602 | Cold cuts & cured meats | 0.0 | 0.0 | 0.0 |
| 2604 | Bacon | 0.0 | 0.0 | 0.0 |
| 2606 | Frankfurters | 0.0 | 0.0 | 0.0 |
| 2608 | Sausages | 0.0 | 0.0 | 0.0 |
| **2802** | **Beans, peas, legumes** | **0.3** | **4.2** | **3.9** |
| **2804** | **Nuts & seeds** | **0.0** | **0.3** | **1.4** |
| 2806 | Processed soy products | 0.0 | 0.2 | 0.1 |
| **3002** | **Meat mixed dishes** | **0.0** | **0.5** | **1.2** |
| **3004** | **Poultry mixed dishes** | **0.0** | **0.4** | **1.0** |
| 3006 | Seafood mixed dishes | 0.0 | 0.0 | 0.2 |
| **3202** | **Rice mixed dishes** | **0.0** | **0.7** | **1.1** |
| **3204** | **Pasta mixed dishes, excludes macaroni & cheese** | **0.0** | **2.5** | **5.2** |
| **3206** | **Macaroni & cheese** | **0.0** | **1.0** | **2.0** |
| 3208 | Turnovers & other grain-based items | 0.0 | 0.1 | 0.2 |
| 3402 | Fried rice & lo/chow mein | 0.0 | 0.0 | 0.3 |
| 3404 | Stir-fry & soy-based sauce mixtures | 0.0 | 0.1 | 0.1 |
| 3406 | Egg rolls, dumplings, sushi | 0.0 | 0.0 | 0.0 |
| 3502 | Burritos & tacos | 0.0 | 0.0 | 0.4 |
| 3504 | Nachos | 0.0 | 0.0 | 0.0 |
| 3506 | Other Mexican mixed dishes | 0.0 | 0.1 | 0.6 |
| **3602** | **Pizza** | **0.0** | **0.1** | **1.5** |
| 3702 | Burgers (single code) | 0.0 | 0.1 | 0.2 |
| 3704 | Chicken/turkey sandwiches (single code) | 0.0 | 0.0 | 0.1 |
| 3706 | Egg/breakfast sandwiches (single code) | 0.0 | 0.0 | 0.0 |
| 3708 | Other sandwiches (single code) | 0.0 | 0.0 | 0.2 |
| **3802** | **Soups** | **0.4** | **1.7** | **2.1** |
| 4002 | Rice | 0.0 | 0.5 | 0.4 |
| 4004 | Pasta, noodles, cooked grains | 0.0 | 0.1 | 0.4 |
| **4202** | **Yeast breads** | **0.0** | **1.7** | **4.9** |
| 4204 | Rolls & buns | 0.0 | 0.1 | 0.7 |
| 4206 | Bagels & English muffins | 0.5 | 0.0 | 0.4 |
| **4208** | **Tortillas** | **0.0** | **0.7** | **1.3** |
| 4402 | Biscuits, muffins, quick breads | 0.0 | 0.2 | 0.6 |
| 4404 | Pancakes, waffles, French toast | 0.0 | 0.3 | 1.2 |
| **4602** | **Ready-to-eat cereal, higher sugar (>21.2g/100g)** | **0.0** | **0.5** | **2.4** |
| **4604** | **Ready-to-eat cereal, lower sugar (≤21.2g/100g)** | **0.0** | **2.1** | **3.8** |
| **4802** | **Oatmeal** | **0.0** | **0.9** | **3.0** |
| 4804 | Grits & other cooked cereals | 0.0 | 0.2 | 0.3 |
| 5002 | Potato chips | 0.0 | 0.0 | 0.7 |
| **5004** | **Tortilla, corn, other chips** | **0.0** | **0.6** | **1.4** |
| 5006 | Popcorn | 0.0 | 0.0 | 0.3 |
| 5008 | Pretzels/snack mix | 0.0 | 0.1 | 0.6 |
| **5202** | **Crackers, excludes saltines** | **0.0** | **0.5** | **1.6** |
| 5204 | Saltine crackers | 0.0 | 0.2 | 0.3 |
| 5402 | Cereal bars | 0.0 | 0.1 | 0.8 |
| 5404 | Nutrition bars | 0.0 | 0.0 | 0.0 |
| 5502 | Cakes & pies | 0.0 | 0.2 | 0.3 |
| **5504** | **Cookies & brownies** | **0.3** | **1.1** | **2.0** |
| 5506 | Doughnuts, sweet rolls, pastries | 0.0 | 0.1 | 0.5 |
| 5702 | Candy containing chocolate | 0.0 | 0.0 | 0.3 |
| 5704 | Candy not containing chocolate | 0.0 | 0.0 | 0.0 |
| 5802 | Ice cream & frozen dairy desserts | 0.0 | 0.2 | 0.4 |
| 5804 | Pudding | 0.0 | 0.0 | 0.0 |
| 5806 | Gelatins, ices, sorbets | 0.0 | 0.0 | 0.1 |
| **6002** | **Apples** | **0.8** | **2.6** | **4.6** |
| **6004** | **Bananas** | **1.5** | **5.3** | **7.1** |
| 6006 | Grapes | 0.0 | 0.1 | 0.7 |
| **6008** | **Peaches & nectarines** | **0.0** | **0.6** | **1.1** |
| **6010** | **Berries** | **0.0** | **0.4** | **1.7** |
| **6012** | **Citrus fruits** | **0.2** | **0.5** | **1.9** |
| 6014 | Melons | 0.0 | 0.1 | 0.4 |
| 6016 | Dried fruits | 0.0 | 0.8 | 0.9 |
| **6018** | **Other fruits & fruit salads** | **0.1** | **1.3** | **3.3** |
| 6402 | Tomatoes | 0.0 | 0.0 | 0.3 |
| 6404 | Carrots | 0.1 | 0.6 | 0.7 |
| **6406** | **Other red & orange vegetables** | **0.0** | **1.1** | **0.6** |
| **6408** | **Dark green vegetables, excludes lettuce** | **0.0** | **0.4** | **1.0** |
| 6410 | Lettuce & lettuce salads | 0.0 | 0.0 | 0.1 |
| **6412** | **String beans** | **0.0** | **0.6** | **1.7** |
| 6414 | Onions | 0.0 | 0.0 | 0.0 |
| 6416 | Corn | 0.0 | 0.2 | 0.9 |
| **6418** | **Other starchy vegetables** | **1.4** | **1.3** | **1.0** |
| **6420** | **Other vegetables & combinations** | **0.2** | **1.4** | **1.5** |
| 6422 | Vegetable mixed dishes | 0.0 | 0.0 | 0.5 |
| 6802 | White potatoes, baked or boiled | 0.0 | 0.1 | 0.4 |
| **6804** | **French fries & other fried white potatoes** | **0.0** | **0.6** | **2.2** |
| **6806** | **Mashed potatoes & white potato mixtures** | **0.8** | **0.9** | **1.0** |
| 7002 | Citrus juice | 0.0 | 0.1 | 0.7 |
| **7004** | **Apple juice** | **0.3** | **0.4** | **1.5** |
| **7006** | **Other fruit juice** | **0.5** | **0.5** | **1.4** |
| 7008 | Vegetable juice | 0.0 | 0.0 | 0.1 |
| 7102 | Diet soft drinks | 0.0 | 0.0 | 0.0 |
| 7106 | Other diet drinks | 0.0 | 0.0 | 0.0 |
| 7202 | Soft drinks | 0.0 | 0.0 | 0.0 |
| 7204 | Fruit drinks | 0.0 | 0.1 | 0.7 |
| 7206 | Sport & energy drinks | 0.0 | 0.0 | 0.0 |
| 7208 | Nutritional beverages | 0.0 | 0.0 | 0.0 |
| 7302 | Coffee | 0.0 | 0.0 | 0.0 |
| 7304 | Tea | 0.0 | 0.0 | 0.0 |
| 7702 | Tap water | 0.0 | 0.0 | 0.0 |
| 7704 | Bottled water | 0.0 | 0.0 | 0.0 |
| 7802 | Flavored or carbonated water | 0.0 | 0.0 | 0.0 |
| 7804 | Enhanced or fortified water | 0.0 | 0.0 | 0.0 |
| 8002 | Butter & animal fats | 0.0 | 0.0 | 0.0 |
| 8004 | Margarine | 0.0 | 0.0 | 0.0 |
| 8006 | Cream cheese, sour cream, whipped cream | 0.0 | 0.0 | 0.0 |
| 8008 | Cream & cream substitutes | 0.0 | 0.0 | 0.0 |
| 8010 | Mayonnaise | 0.0 | 0.0 | 0.0 |
| 8012 | Salad dressings & vegetable oils | 0.0 | 0.0 | 0.0 |
| 8402 | Tomato-based condiments | 0.0 | 0.0 | 0.0 |
| 8404 | Soy-based condiments | 0.0 | 0.0 | 0.0 |
| 8406 | Mustard & other condiments | 0.0 | 0.0 | 0.0 |
| 8408 | Olives, pickles, pickled vegetables | 0.0 | 0.0 | 0.2 |
| 8410 | Pasta sauces, tomato-based | 0.0 | 0.0 | 0.2 |
| 8412 | Dips, gravies, other sauces | 0.0 | 0.0 | 0.1 |
| 8802 | Sugars & honey | 0.0 | 0.0 | 0.0 |
| 8804 | Sugar substitutes | 0.0 | 0.0 | 0.0 |
| 8806 | Jams, syrups, toppings | 0.0 | 0.0 | 0.2 |
| **9002** | **Baby food: cereals** | **30.8** | **11.5** | **2.0** |
| **9004** | **Baby food: fruit** | **26.6** | **19.9** | **1.4** |
| **9006** | **Baby food: vegetable** | **27.1** | **14.7** | **0.9** |
| **9008** | **Baby food: meat & dinners** | **2.5** | **7.7** | **1.6** |
| 9010 | Baby food: yogurt | 0.1 | 0.5 | 0.1 |
| **9012** | **Baby food: snacks & sweets** | **0.4** | **1.3** | **0.4** |
| **9202** | **Baby juice** | **1.6** | **0.8** | **0.2** |
| 9204 | Baby water | 0.0 | 0.0 | 0.0 |
| **9402** | **Formula, ready-to-feed** | **3.5** | **0.0** | **0.0** |
| 9404 | Formula, prepared from powder | 0.0 | 0.0 | 0.0 |
| 9406 | Formula, prepared from concentrate | 0.0 | 0.0 | 0.0 |
| 9602 | Human milk | 0.0 | 0.0 | 0.0 |
| 9999 | Not included in a food category | 0.0 | 0.0 | 0.1 |
|  | Total | 100 | 100 | 100 |

^a^ Contributions from all minor WWEIA food categories are reported. Those in bold contribute ≥1% to daily intake in at least one age group.

**Table S8.** Food sources of Vitamin A among U.S. infants and toddlers aged 0–23.9 months by age group: NHANES 2005-12 (*n* = 2740) ^a^.

| **WWEIA Minor Food category code** | **Food category** | **% of daily intake** | | |
| --- | --- | --- | --- | --- |
|  |  | **0–5.9 mo (*n* = 765)** | **6–11.9 mo (*n* = 854)** | **12–23.9 mo  (*n* = 1121)** |
| **1002** | **Milk, whole** | **0.0** | **2.1** | **26.5** |
| **1004** | **Milk, reduced fat** | **0.0** | **0.6** | **8.9** |
| 1006 | Milk, low-fat | 0.0 | 0.3 | 0.9 |
| 1008 | Milk, non-fat | 0.0 | 0.0 | 1.1 |
| 1202 | Flavored milk, whole | 0.0 | 0.0 | 0.7 |
| 1204 | Flavored milk, reduced fat | 0.0 | 0.0 | 0.8 |
| 1206 | Flavored milk, low-fat | 0.0 | 0.0 | 0.3 |
| 1208 | Flavored milk, non-fat | 0.0 | 0.0 | 0.0 |
| 1402 | Milk shakes & other dairy drinks | 0.0 | 0.0 | 0.0 |
| **1404** | **Milk substitutes** | **0.0** | **0.3** | **1.5** |
| **1602** | **Cheese** | **0.0** | **0.6** | **3.3** |
| 1604 | Cottage/ricotta cheese | 0.0 | 0.0 | 0.1 |
| 1802 | Yogurt, whole & reduced fat | 0.0 | 0.2 | 0.4 |
| 1804 | Yogurt, low-fat & non-fat | 0.0 | 0.1 | 0.5 |
| 2002 | Beef, excludes ground | 0.0 | 0.0 | 0.0 |
| 2004 | Ground beef | 0.0 | 0.0 | 0.0 |
| 2006 | Pork | 0.0 | 0.0 | 0.0 |
| 2008 | Lamb, goat, game | 0.0 | 0.0 | 0.0 |
| 2010 | Liver & organ meats | 0.0 | 0.0 | 0.8 |
| 2202 | Chicken, whole pieces | 0.0 | 0.0 | 0.3 |
| 2204 | Chicken patties, nuggets & tenders | 0.0 | 0.0 | 0.1 |
| 2206 | Turkey, duck, other poultry | 0.0 | 0.0 | 0.0 |
| 2402 | Fish | 0.0 | 0.0 | 0.1 |
| 2404 | Shellfish | 0.0 | 0.0 | 0.0 |
| **2502** | **Eggs & omelets** | **0.0** | **0.7** | **4.0** |
| 2602 | Cold cuts & cured meats | 0.0 | 0.0 | 0.0 |
| 2604 | Bacon | 0.0 | 0.0 | 0.0 |
| 2606 | Frankfurters | 0.0 | 0.0 | 0.0 |
| 2608 | Sausages | 0.0 | 0.0 | 0.1 |
| 2802 | Beans, peas, legumes | 0.0 | 0.0 | 0.0 |
| 2804 | Nuts & seeds | 0.0 | 0.0 | 0.1 |
| 2806 | Processed soy products | 0.0 | 0.1 | 0.0 |
| 3002 | Meat mixed dishes | 0.0 | 0.1 | 0.5 |
| 3004 | Poultry mixed dishes | 0.0 | 0.1 | 0.9 |
| 3006 | Seafood mixed dishes | 0.0 | 0.0 | 0.1 |
| 3202 | Rice mixed dishes | 0.0 | 0.1 | 0.2 |
| **3204** | **Pasta mixed dishes, excludes macaroni & cheese** | **0.0** | **0.4** | **1.1** |
| **3206** | **Macaroni & cheese** | **0.0** | **0.4** | **1.8** |
| 3208 | Turnovers & other grain-based items | 0.0 | 0.0 | 0.1 |
| 3402 | Fried rice & lo/chow mein | 0.0 | 0.0 | 0.0 |
| 3404 | Stir-fry & soy-based sauce mixtures | 0.0 | 0.0 | 0.1 |
| 3406 | Egg rolls, dumplings, sushi | 0.0 | 0.0 | 0.1 |
| 3502 | Burritos & tacos | 0.0 | 0.0 | 0.1 |
| 3504 | Nachos | 0.0 | 0.0 | 0.0 |
| 3506 | Other Mexican mixed dishes | 0.0 | 0.0 | 0.2 |
| 3602 | Pizza | 0.0 | 0.0 | 0.7 |
| 3702 | Burgers (single code) | 0.0 | 0.0 | 0.1 |
| 3704 | Chicken/turkey sandwiches (single code) | 0.0 | 0.0 | 0.0 |
| 3706 | Egg/breakfast sandwiches (single code) | 0.0 | 0.0 | 0.0 |
| 3708 | Other sandwiches (single code) | 0.0 | 0.0 | 0.1 |
| **3802** | **Soups** | **0.0** | **0.6** | **1.2** |
| 4002 | Rice | 0.0 | 0.0 | 0.0 |
| 4004 | Pasta, noodles, cooked grains | 0.0 | 0.0 | 0.0 |
| 4202 | Yeast breads | 0.0 | 0.0 | 0.1 |
| 4204 | Rolls & buns | 0.0 | 0.0 | 0.0 |
| 4206 | Bagels & English muffins | 0.0 | 0.0 | 0.0 |
| 4208 | Tortillas | 0.0 | 0.0 | 0.0 |
| 4402 | Biscuits, muffins, quick breads | 0.0 | 0.0 | 0.1 |
| **4404** | **Pancakes, waffles, French toast** | **0.0** | **0.1** | **1.2** |
| **4602** | **Ready-to-eat cereal, higher sugar (>21.2g/100g)** | **0.0** | **0.3** | **3.6** |
| **4604** | **Ready-to-eat cereal, lower sugar (≤21.2g/100g)** | **0.0** | **1.5** | **4.5** |
| **4802** | **Oatmeal** | **0.0** | **0.6** | **3.4** |
| 4804 | Grits & other cooked cereals | 0.0 | 0.1 | 0.3 |
| 5002 | Potato chips | 0.0 | 0.0 | 0.0 |
| 5004 | Tortilla, corn, other chips | 0.0 | 0.0 | 0.1 |
| 5006 | Popcorn | 0.0 | 0.0 | 0.0 |
| 5008 | Pretzels/snack mix | 0.0 | 0.0 | 0.0 |
| 5202 | Crackers, excludes saltines | 0.0 | 0.0 | 0.0 |
| 5204 | Saltine crackers | 0.0 | 0.0 | 0.0 |
| **5402** | **Cereal bars** | **0.0** | **0.1** | **1.0** |
| 5404 | Nutrition bars | 0.0 | 0.0 | 0.0 |
| 5502 | Cakes & pies | 0.0 | 0.0 | 0.3 |
| 5504 | Cookies & brownies | 0.0 | 0.1 | 0.2 |
| 5506 | Doughnuts, sweet rolls, pastries | 0.0 | 0.1 | 0.5 |
| 5702 | Candy containing chocolate | 0.0 | 0.0 | 0.1 |
| 5704 | Candy not containing chocolate | 0.0 | 0.0 | 0.0 |
| 5802 | Ice cream & frozen dairy desserts | 0.0 | 0.1 | 0.8 |
| 5804 | Pudding | 0.0 | 0.0 | 0.0 |
| 5806 | Gelatins, ices, sorbets | 0.0 | 0.0 | 0.0 |
| 6002 | Apples | 0.0 | 0.0 | 0.1 |
| 6004 | Bananas | 0.0 | 0.1 | 0.1 |
| 6006 | Grapes | 0.0 | 0.0 | 0.0 |
| 6008 | Peaches & nectarines | 0.0 | 0.1 | 0.2 |
| 6010 | Berries | 0.0 | 0.0 | 0.0 |
| 6012 | Citrus fruits | 0.0 | 0.1 | 0.5 |
| 6014 | Melons | 0.0 | 0.1 | 0.7 |
| 6016 | Dried fruits | 0.0 | 0.0 | 0.0 |
| 6018 | Other fruits & fruit salads | 0.0 | 0.1 | 0.3 |
| 6402 | Tomatoes | 0.0 | 0.0 | 0.2 |
| **6404** | **Carrots** | **0.0** | **1.3** | **2.8** |
| **6406** | **Other red & orange vegetables** | **0.0** | **1.3** | **1.6** |
| 6408 | Dark green vegetables, excludes lettuce | 0.0 | 0.1 | 0.7 |
| 6410 | Lettuce & lettuce salads | 0.0 | 0.0 | 0.0 |
| 6412 | String beans | 0.0 | 0.0 | 0.3 |
| 6414 | Onions | 0.0 | 0.0 | 0.0 |
| 6416 | Corn | 0.0 | 0.0 | 0.1 |
| 6418 | Other starchy vegetables | 0.0 | 0.1 | 0.2 |
| **6420** | **Other vegetables & combinations** | **0.0** | **0.8** | **1.3** |
| 6422 | Vegetable mixed dishes | 0.0 | 0.0 | 0.4 |
| 6802 | White potatoes, baked or boiled | 0.0 | 0.0 | 0.0 |
| 6804 | French fries & other fried white potatoes | 0.0 | 0.0 | 0.0 |
| 6806 | Mashed potatoes & white potato mixtures | 0.0 | 0.2 | 0.4 |
| 7002 | Citrus juice | 0.0 | 0.0 | 0.1 |
| 7004 | Apple juice | 0.0 | 0.0 | 0.0 |
| 7006 | Other fruit juice | 0.0 | 0.0 | 0.2 |
| **7008** | **Vegetable juice** | **0.0** | **0.1** | **1.0** |
| 7102 | Diet soft drinks | 0.0 | 0.0 | 0.0 |
| 7106 | Other diet drinks | 0.0 | 0.0 | 0.0 |
| 7202 | Soft drinks | 0.0 | 0.0 | 0.0 |
| 7204 | Fruit drinks | 0.0 | 0.0 | 0.6 |
| 7206 | Sport & energy drinks | 0.0 | 0.0 | 0.0 |
| 7208 | Nutritional beverages | 0.0 | 0.0 | 0.5 |
| 7302 | Coffee | 0.0 | 0.0 | 0.0 |
| 7304 | Tea | 0.0 | 0.0 | 0.0 |
| 7702 | Tap water | 0.0 | 0.0 | 0.0 |
| 7704 | Bottled water | 0.0 | 0.0 | 0.0 |
| 7802 | Flavored or carbonated water | 0.0 | 0.0 | 0.0 |
| 7804 | Enhanced or fortified water | 0.0 | 0.0 | 0.0 |
| 8002 | Butter & animal fats | 0.0 | 0.1 | 0.4 |
| 8004 | Margarine | 0.0 | 0.1 | 0.6 |
| 8006 | Cream cheese, sour cream, whipped cream | 0.0 | 0.0 | 0.1 |
| 8008 | Cream & cream substitutes | 0.0 | 0.0 | 0.1 |
| 8010 | Mayonnaise | 0.0 | 0.0 | 0.0 |
| 8012 | Salad dressings & vegetable oils | 0.0 | 0.0 | 0.0 |
| 8402 | Tomato-based condiments | 0.0 | 0.0 | 0.0 |
| 8404 | Soy-based condiments | 0.0 | 0.0 | 0.0 |
| 8406 | Mustard & other condiments | 0.0 | 0.0 | 0.0 |
| 8408 | Olives, pickles, pickled vegetables | 0.0 | 0.0 | 0.0 |
| 8410 | Pasta sauces, tomato-based | 0.0 | 0.0 | 0.0 |
| 8412 | Dips, gravies, other sauces | 0.0 | 0.0 | 0.0 |
| 8802 | Sugars & honey | 0.0 | 0.0 | 0.0 |
| 8804 | Sugar substitutes | 0.0 | 0.0 | 0.0 |
| 8806 | Jams, syrups, toppings | 0.0 | 0.0 | 0.0 |
| 9002 | Baby food: cereals | 0.0 | 0.0 | 0.0 |
| 9004 | Baby food: fruit | 0.0 | 0.6 | 0.1 |
| **9006** | **Baby food: vegetable** | **3.1** | **15.9** | **2.2** |
| **9008** | **Baby food: meat & dinners** | **0.1** | **4.5** | **2.4** |
| 9010 | Baby food: yogurt | 0.0 | 0.1 | 0.1 |
| 9012 | Baby food: snacks & sweets | 0.0 | 0.2 | 0.1 |
| 9202 | Baby juice | 0.0 | 0.2 | 0.0 |
| 9204 | Baby water | 0.0 | 0.0 | 0.0 |
| **9402** | **Formula, ready-to-feed** | **2.2** | **2.9** | **1.1** |
| **9404** | **Formula, prepared from powder** | **59.1** | **46.3** | **2.9** |
| **9406** | **Formula, prepared from concentrate** | **5.3** | **3.8** | **0.1** |
| **9602** | **Human milk** | **29.9** | **10.8** | **2.5** |
| 9999 | Not included in a food category | 0.0 | 0.0 | 0.7 |
|  | Total | 100 | 100 | 100 |

^a^ Contributions from all minor WWEIA food categories are reported. Those in bold contribute ≥1% to daily intake in at least one age group.

**Table S9.** Food sources of folate among U.S. infants and toddlers aged 0–23.9 months by age group: NHANES 2005-12 (*n* = 2740) ^a^.

| **WWEIA Minor Food category code** | **Food category** | **% of daily intake** | | |
| --- | --- | --- | --- | --- |
|  |  | **0–5.9 mo (*n* = 765)** | **6–11.9 mo (*n* = 854)** | **12–23.9 mo  (*n* = 1121)** |
| **1002** | **Milk, whole** | **0.0** | **1.3** | **8.2** |
| **1004** | **Milk, reduced fat** | **0.0** | **0.3** | **2.1** |
| 1006 | Milk, lowfat | 0.0 | 0.1 | 0.2 |
| 1008 | Milk, nonfat | 0.0 | 0.0 | 0.2 |
| 1202 | Flavored milk, whole | 0.0 | 0.0 | 0.2 |
| 1204 | Flavored milk, reduced fat | 0.0 | 0.0 | 0.1 |
| 1206 | Flavored milk, lowfat | 0.0 | 0.0 | 0.1 |
| 1208 | Flavored milk, nonfat | 0.0 | 0.0 | 0.0 |
| 1402 | Milk shakes and other dairy drinks | 0.0 | 0.0 | 0.0 |
| 1404 | Milk substitutes | 0.0 | 0.2 | 0.7 |
| 1602 | Cheese | 0.0 | 0.2 | 0.5 |
| 1604 | Cottage/ricotta cheese | 0.0 | 0.0 | 0.1 |
| 1802 | Yogurt, whole and reduced fat | 0.0 | 0.3 | 0.4 |
| 1804 | Yogurt, lowfat and nonfat | 0.0 | 0.3 | 0.5 |
| 2002 | Beef, excludes ground | 0.0 | 0.0 | 0.1 |
| 2004 | Ground beef | 0.0 | 0.0 | 0.1 |
| 2006 | Pork | 0.0 | 0.0 | 0.0 |
| 2008 | Lamb, goat, game | 0.0 | 0.0 | 0.0 |
| 2010 | Liver and organ meats | 0.0 | 0.0 | 0.2 |
| 2202 | Chicken, whole pieces | 0.0 | 0.1 | 0.4 |
| 2204 | Chicken patties, nuggets and tenders | 0.0 | 0.1 | 0.6 |
| 2206 | Turkey, duck, other poultry | 0.0 | 0.0 | 0.0 |
| 2402 | Fish | 0.0 | 0.0 | 0.1 |
| 2404 | Shellfish | 0.0 | 0.0 | 0.0 |
| **2502** | **Eggs and omelets** | **0.0** | **0.8** | **2.1** |
| 2602 | Cold cuts and cured meats | 0.0 | 0.0 | 0.1 |
| 2604 | Bacon | 0.0 | 0.0 | 0.0 |
| 2606 | Frankfurters | 0.0 | 0.0 | 0.1 |
| 2608 | Sausages | 0.0 | 0.0 | 0.0 |
| **2802** | **Beans, peas, legumes** | **0.0** | **2.2** | **1.8** |
| 2804 | Nuts and seeds | 0.0 | 0.1 | 0.7 |
| 2806 | Processed soy products | 0.0 | 0.1 | 0.2 |
| 3002 | Meat mixed dishes | 0.0 | 0.3 | 0.7 |
| **3004** | **Poultry mixed dishes** | **0.0** | **0.2** | **1.1** |
| 3006 | Seafood mixed dishes | 0.0 | 0.0 | 0.2 |
| **3202** | **Rice mixed dishes** | **0.0** | **0.7** | **1.1** |
| **3204** | **Pasta mixed dishes, excludes macaroni and cheese** | **0.0** | **1.8** | **3.8** |
| **3206** | **Macaroni and cheese** | **0.0** | **1.4** | **3.0** |
| 3208 | Turnovers and other grain-based items | 0.0 | 0.1 | 0.2 |
| 3402 | Fried rice and lo/chow mein | 0.0 | 0.0 | 0.4 |
| 3404 | Stir-fry and soy-based sauce mixtures | 0.0 | 0.1 | 0.1 |
| 3406 | Egg rolls, dumplings, sushi | 0.0 | 0.0 | 0.1 |
| 3502 | Burritos and tacos | 0.0 | 0.0 | 0.3 |
| 3504 | Nachos | 0.0 | 0.0 | 0.0 |
| 3506 | Other Mexican mixed dishes | 0.0 | 0.1 | 0.4 |
| **3602** | **Pizza** | **0.0** | **0.2** | **2.0** |
| 3702 | Burgers (single code) | 0.0 | 0.1 | 0.2 |
| 3704 | Chicken/turkey sandwiches (single code) | 0.0 | 0.0 | 0.1 |
| 3706 | Egg/breakfast sandwiches (single code) | 0.0 | 0.0 | 0.0 |
| 3708 | Other sandwiches (single code) | 0.0 | 0.0 | 0.4 |
| **3802** | **Soups** | **0.1** | **1.4** | **1.8** |
| **4002** | **Rice** | **0.0** | **0.8** | **1.4** |
| 4004 | Pasta, noodles, cooked grains | 0.0 | 0.1 | 0.6 |
| **4202** | **Yeast breads** | **0.0** | **1.6** | **5.0** |
| **4204** | **Rolls and buns** | **0.0** | **0.2** | **1.4** |
| 4206 | Bagels and English muffins | 0.0 | 0.0 | 0.5 |
| 4208 | Tortillas | 0.0 | 0.4 | 0.4 |
| 4402 | Biscuits, muffins, quick breads | 0.0 | 0.2 | 0.6 |
| **4404** | **Pancakes, waffles, French toast** | **0.0** | **0.3** | **1.4** |
| **4602** | **Ready-to-eat cereal, higher sugar (>21.2g/100g)** | **0.0** | **1.7** | **8.8** |
| **4604** | **Ready-to-eat cereal, lower sugar (≤21.2g/100g)** | **0.0** | **7.7** | **12.8** |
| **4802** | **Oatmeal** | **0.0** | **0.8** | **2.1** |
| 4804 | Grits and other cooked cereals | 0.0 | 0.3 | 0.4 |
| 5002 | Potato chips | 0.0 | 0.0 | 0.4 |
| 5004 | Tortilla, corn, other chips | 0.0 | 0.3 | 0.5 |
| 5006 | Popcorn | 0.0 | 0.0 | 0.0 |
| **5008** | **Pretzels/snack mix** | **0.0** | **0.1** | **1.1** |
| **5202** | **Crackers, excludes saltines** | **0.0** | **0.7** | **2.8** |
| 5204 | Saltine crackers | 0.0 | 0.3 | 0.5 |
| 5402 | Cereal bars | 0.0 | 0.1 | 0.8 |
| 5404 | Nutrition bars | 0.0 | 0.0 | 0.0 |
| 5502 | Cakes and pies | 0.0 | 0.1 | 0.3 |
| **5504** | **Cookies and brownies** | **0.0** | **1.5** | **2.6** |
| 5506 | Doughnuts, sweet rolls, pastries | 0.0 | 0.2 | 0.9 |
| 5702 | Candy containing chocolate | 0.0 | 0.0 | 0.1 |
| 5704 | Candy not containing chocolate | 0.0 | 0.0 | 0.0 |
| 5802 | Ice cream and frozen dairy desserts | 0.0 | 0.0 | 0.2 |
| 5804 | Pudding | 0.0 | 0.0 | 0.0 |
| 5806 | Gelatins, ices, sorbets | 0.0 | 0.0 | 0.0 |
| 6002 | Apples | 0.0 | 0.1 | 0.2 |
| **6004** | **Bananas** | **0.1** | **1.5** | **2.1** |
| 6006 | Grapes | 0.0 | 0.0 | 0.1 |
| 6008 | Peaches and nectarines | 0.0 | 0.1 | 0.1 |
| 6010 | Berries | 0.0 | 0.1 | 0.4 |
| 6012 | Citrus fruits | 0.0 | 0.2 | 0.8 |
| 6014 | Melons | 0.0 | 0.1 | 0.2 |
| 6016 | Dried fruits | 0.0 | 0.0 | 0.0 |
| 6018 | Other fruits and fruit salads | 0.0 | 0.3 | 0.5 |
| 6402 | Tomatoes | 0.0 | 0.0 | 0.2 |
| 6404 | Carrots | 0.0 | 0.1 | 0.1 |
| 6406 | Other red and orange vegetables | 0.0 | 0.2 | 0.1 |
| 6408 | Dark green vegetables, excludes lettuce | 0.0 | 0.5 | 1.1 |
| 6410 | Lettuce and lettuce salads | 0.0 | 0.0 | 0.1 |
| 6412 | String beans | 0.0 | 0.2 | 0.7 |
| 6414 | Onions | 0.0 | 0.0 | 0.0 |
| 6416 | Corn | 0.0 | 0.1 | 0.5 |
| 6418 | Other starchy vegetables | 0.0 | 0.3 | 0.3 |
| 6420 | Other vegetables and combinations | 0.0 | 0.5 | 0.7 |
| 6422 | Vegetable mixed dishes | 0.0 | 0.0 | 0.3 |
| 6802 | White potatoes, baked or boiled | 0.0 | 0.0 | 0.1 |
| 6804 | French fries and other fried white potatoes | 0.0 | 0.2 | 0.7 |
| 6806 | Mashed potatoes and white potato mixtures | 0.0 | 0.2 | 0.2 |
| 7002 | Citrus juice | 0.0 | 0.3 | 1.9 |
| 7004 | Apple juice | 0.0 | 0.0 | 0.0 |
| 7006 | Other fruit juice | 0.0 | 0.2 | 0.6 |
| 7008 | Vegetable juice | 0.0 | 0.0 | 0.1 |
| 7102 | Diet soft drinks | 0.0 | 0.0 | 0.0 |
| 7106 | Other diet drinks | 0.0 | 0.0 | 0.0 |
| 7202 | Soft drinks | 0.0 | 0.0 | 0.0 |
| 7204 | Fruit drinks | 0.0 | 0.1 | 0.6 |
| 7206 | Sport and energy drinks | 0.0 | 0.0 | 0.0 |
| 7208 | Nutritional beverages | 0.0 | 0.0 | 0.4 |
| 7302 | Coffee | 0.0 | 0.0 | 0.0 |
| 7304 | Tea | 0.0 | 0.1 | 0.3 |
| 7702 | Tap water | 0.0 | 0.0 | 0.0 |
| 7704 | Bottled water | 0.0 | 0.0 | 0.0 |
| 7802 | Flavored or carbonated water | 0.0 | 0.0 | 0.0 |
| 7804 | Enhanced or fortified water | 0.0 | 0.0 | 0.0 |
| 8002 | Butter and animal fats | 0.0 | 0.0 | 0.0 |
| 8004 | Margarine | 0.0 | 0.0 | 0.0 |
| 8006 | Cream cheese, sour cream, whipped cream | 0.0 | 0.0 | 0.0 |
| 8008 | Cream and cream substitutes | 0.0 | 0.0 | 0.0 |
| 8010 | Mayonnaise | 0.0 | 0.0 | 0.0 |
| 8012 | Salad dressings and vegetable oils | 0.0 | 0.0 | 0.0 |
| 8402 | Tomato-based condiments | 0.0 | 0.0 | 0.0 |
| 8404 | Soy-based condiments | 0.0 | 0.0 | 0.0 |
| 8406 | Mustard and other condiments | 0.0 | 0.0 | 0.0 |
| 8408 | Olives, pickles, pickled vegetables | 0.0 | 0.0 | 0.0 |
| 8410 | Pasta sauces, tomato-based | 0.0 | 0.0 | 0.0 |
| 8412 | Dips, gravies, other sauces | 0.0 | 0.0 | 0.0 |
| 8802 | Sugars and honey | 0.0 | 0.0 | 0.0 |
| 8804 | Sugar substitutes | 0.0 | 0.0 | 0.0 |
| 8806 | Jams, syrups, toppings | 0.0 | 0.0 | 0.0 |
| **9002** | **Baby food: cereals** | **1.4** | **2.7** | **0.3** |
| **9004** | **Baby food: fruit** | **0.5** | **3.1** | **0.3** |
| **9006** | **Baby food: vegetable** | **1.3** | **5.7** | **0.3** |
| **9008** | **Baby food: meat and dinners** | **0.1** | **2.1** | **0.7** |
| 9010 | Baby food: yogurt | 0.0 | 0.2 | 0.1 |
| 9012 | Baby food: snacks and sweets | 0.0 | 1.1 | 0.4 |
| 9202 | Baby juice | 0.3 | 0.7 | 0.1 |
| 9204 | Baby water | 0.0 | 0.0 | 0.0 |
| **9402** | **Formula, ready-to-feed** | **2.7** | **2.6** | **0.7** |
| **9404** | **Formula, prepared from powder** | **70.3** | **38.7** | **1.2** |
| **9406** | **Formula, prepared from concentrate** | **6.3** | **3.2** | **0.0** |
| **9602** | **Human milk** | **16.8** | **4.4** | **0.5** |
| 9999 | Not included in a food category | 0.0 | 0.0 | 0.3 |
|  | Total | 100 | 100 | 100 |

^a^ Contributions from all minor WWEIA food categories are reported. Those in bold contribute ≥1% to daily intake in at least one age group.

**Table S10.** Food sources of vitamin C among U.S. infants and toddlers aged 0–23.9 months by age group: NHANES 2005-12 (*n* = 2740) ^a^.

| **WWEIA Minor Food category code** | **Food category** | **% of daily intake** | | |
| --- | --- | --- | --- | --- |
|  |  | **0–5.9 mo (*n* = 765)** | **6–11.9 mo (*n* = 854)** | **12–23.9 mo  (*n* = 1121)** |
| 1002 | Milk, whole | 0.0 | 0.0 | 0.0 |
| 1004 | Milk, reduced fat | 0.0 | 0.0 | 0.2 |
| 1006 | Milk, low-fat | 0.0 | 0.0 | 0.0 |
| 1008 | Milk, non-fat | 0.0 | 0.0 | 0.0 |
| 1202 | Flavored milk, whole | 0.0 | 0.0 | 0.1 |
| 1204 | Flavored milk, reduced fat | 0.0 | 0.0 | 0.1 |
| 1206 | Flavored milk, low-fat | 0.0 | 0.0 | 0.0 |
| 1208 | Flavored milk, non-fat | 0.0 | 0.0 | 0.0 |
| 1402 | Milk shakes & other dairy drinks | 0.0 | 0.0 | 0.0 |
| 1404 | Milk substitutes | 0.0 | 0.0 | 0.0 |
| 1602 | Cheese | 0.0 | 0.0 | 0.0 |
| 1604 | Cottage/ricotta cheese | 0.0 | 0.0 | 0.0 |
| 1802 | Yogurt, whole & reduced fat | 0.0 | 0.0 | 0.1 |
| 1804 | Yogurt, low-fat & non-fat | 0.0 | 0.0 | 0.1 |
| 2002 | Beef, excludes ground | 0.0 | 0.0 | 0.0 |
| 2004 | Ground beef | 0.0 | 0.0 | 0.0 |
| 2006 | Pork | 0.0 | 0.0 | 0.0 |
| 2008 | Lamb, goat, game | 0.0 | 0.0 | 0.0 |
| 2010 | Liver & organ meats | 0.0 | 0.0 | 0.0 |
| 2202 | Chicken, whole pieces | 0.0 | 0.0 | 0.0 |
| 2204 | Chicken patties, nuggets & tenders | 0.0 | 0.0 | 0.0 |
| 2206 | Turkey, duck, other poultry | 0.0 | 0.0 | 0.0 |
| 2402 | Fish | 0.0 | 0.0 | 0.0 |
| 2404 | Shellfish | 0.0 | 0.0 | 0.0 |
| 2502 | Eggs & omelets | 0.0 | 0.0 | 0.1 |
| 2602 | Cold cuts & cured meats | 0.0 | 0.0 | 0.0 |
| 2604 | Bacon | 0.0 | 0.0 | 0.0 |
| 2606 | Frankfurters | 0.0 | 0.0 | 0.0 |
| 2608 | Sausages | 0.0 | 0.0 | 0.0 |
| 2802 | Beans, peas, legumes | 0.0 | 0.1 | 0.1 |
| 2804 | Nuts & seeds | 0.0 | 0.0 | 0.0 |
| 2806 | Processed soy products | 0.0 | 0.0 | 0.0 |
| 3002 | Meat mixed dishes | 0.0 | 0.1 | 0.5 |
| 3004 | Poultry mixed dishes | 0.0 | 0.1 | 0.4 |
| 3006 | Seafood mixed dishes | 0.0 | 0.0 | 0.1 |
| 3202 | Rice mixed dishes | 0.0 | 0.1 | 0.4 |
| 3204 | Pasta mixed dishes, excludes macaroni & cheese | 0.0 | 0.2 | 0.8 |
| 3206 | Macaroni & cheese | 0.0 | 0.0 | 0.0 |
| 3208 | Turnovers & other grain-based items | 0.0 | 0.0 | 0.0 |
| 3402 | Fried rice & lo/chow mein | 0.0 | 0.0 | 0.1 |
| 3404 | Stir-fry & soy-based sauce mixtures | 0.0 | 0.0 | 0.1 |
| 3406 | Egg rolls, dumplings, sushi | 0.0 | 0.0 | 0.0 |
| 3502 | Burritos & tacos | 0.0 | 0.0 | 0.0 |
| 3504 | Nachos | 0.0 | 0.0 | 0.0 |
| 3506 | Other Mexican mixed dishes | 0.0 | 0.0 | 0.1 |
| 3602 | Pizza | 0.0 | 0.0 | 0.2 |
| 3702 | Burgers (single code) | 0.0 | 0.0 | 0.0 |
| 3704 | Chicken/turkey sandwiches (single code) | 0.0 | 0.0 | 0.0 |
| 3706 | Egg/breakfast sandwiches (single code) | 0.0 | 0.0 | 0.0 |
| 3708 | Other sandwiches (single code) | 0.0 | 0.0 | 0.0 |
| 3802 | Soups | 0.0 | 0.4 | 0.9 |
| 4002 | Rice | 0.0 | 0.0 | 0.0 |
| 4004 | Pasta, noodles, cooked grains | 0.0 | 0.0 | 0.0 |
| 4202 | Yeast breads | 0.0 | 0.0 | 0.0 |
| 4204 | Rolls & buns | 0.0 | 0.0 | 0.0 |
| 4206 | Bagels & English muffins | 0.0 | 0.0 | 0.0 |
| 4208 | Tortillas | 0.0 | 0.0 | 0.0 |
| 4402 | Biscuits, muffins, quick breads | 0.0 | 0.0 | 0.0 |
| 4404 | Pancakes, waffles, French toast | 0.0 | 0.0 | 0.0 |
| **4602** | **Ready-to-eat cereal, higher sugar (>21.2g/100g)** | **0.0** | **0.1** | **1.2** |
| 4604 | Ready-to-eat cereal, lower sugar (≤21.2g/100g) | 0.0 | 0.3 | 0.9 |
| 4802 | Oatmeal | 0.0 | 0.0 | 0.0 |
| 4804 | Grits & other cooked cereals | 0.0 | 0.0 | 0.0 |
| 5002 | Potato chips | 0.0 | 0.0 | 0.3 |
| 5004 | Tortilla, corn, other chips | 0.0 | 0.0 | 0.0 |
| 5006 | Popcorn | 0.0 | 0.0 | 0.0 |
| 5008 | Pretzels/snack mix | 0.0 | 0.0 | 0.0 |
| 5202 | Crackers, excludes saltines | 0.0 | 0.0 | 0.0 |
| 5204 | Saltine crackers | 0.0 | 0.0 | 0.0 |
| 5402 | Cereal bars | 0.0 | 0.0 | 0.0 |
| 5404 | Nutrition bars | 0.0 | 0.0 | 0.0 |
| 5502 | Cakes & pies | 0.0 | 0.1 | 0.0 |
| 5504 | Cookies & brownies | 0.0 | 0.0 | 0.0 |
| 5506 | Doughnuts, sweet rolls, pastries | 0.0 | 0.0 | 0.0 |
| 5702 | Candy containing chocolate | 0.0 | 0.0 | 0.0 |
| **5704** | **Candy not containing chocolate** | **0.0** | **0.1** | **2.0** |
| 5802 | Ice cream & frozen dairy desserts | 0.0 | 0.0 | 0.0 |
| 5804 | Pudding | 0.0 | 0.0 | 0.0 |
| 5806 | Gelatins, ices, sorbets | 0.0 | 0.0 | 0.1 |
| 6002 | Apples | 0.0 | 0.2 | 0.8 |
| **6004** | **Bananas** | **0.0** | **1.0** | **2.4** |
| 6006 | Grapes | 0.0 | 0.0 | 0.6 |
| 6008 | Peaches & nectarines | 0.0 | 0.1 | 0.3 |
| **6010** | **Berries** | **0.0** | **0.4** | **2.6** |
| **6012** | **Citrus fruits** | **0.0** | **0.6** | **4.6** |
| **6014** | **Melons** | **0.0** | **0.2** | **1.2** |
| 6016 | Dried fruits | 0.0 | 0.0 | 0.1 |
| **6018** | **Other fruits & fruit salads** | **0.0** | **0.5** | **1.6** |
| 6402 | Tomatoes | 0.0 | 0.0 | 0.4 |
| 6404 | Carrots | 0.0 | 0.0 | 0.1 |
| 6406 | Other red & orange vegetables | 0.0 | 0.2 | 0.3 |
| **6408** | **Dark green vegetables, excludes lettuce** | **0.0** | **0.4** | **1.6** |
| 6410 | Lettuce & lettuce salads | 0.0 | 0.0 | 0.0 |
| 6412 | String beans | 0.0 | 0.1 | 0.3 |
| 6414 | Onions | 0.0 | 0.0 | 0.0 |
| 6416 | Corn | 0.0 | 0.0 | 0.2 |
| 6418 | Other starchy vegetables | 0.0 | 0.2 | 0.3 |
| 6420 | Other vegetables & combinations | 0.0 | 0.2 | 0.7 |
| 6422 | Vegetable mixed dishes | 0.0 | 0.0 | 0.4 |
| 6802 | White potatoes, baked or boiled | 0.0 | 0.0 | 0.3 |
| 6804 | French fries & other fried white potatoes | 0.0 | 0.1 | 0.5 |
| 6806 | Mashed potatoes & white potato mixtures | 0.0 | 0.2 | 0.5 |
| 7002 | Citrus juice | 0.0 | 0.8 | 8.7 |
| **7004** | **Apple juice** | **0.1** | **1.4** | **9.6** |
| **7006** | **Other fruit juice** | **0.5** | **3.6** | **22.7** |
| 7008 | Vegetable juice | 0.0 | 0.0 | 0.2 |
| 7102 | Diet soft drinks | 0.0 | 0.0 | 0.0 |
| 7106 | Other diet drinks | 0.0 | 0.0 | 0.2 |
| 7202 | Soft drinks | 0.0 | 0.0 | 0.0 |
| **7204** | **Fruit drinks** | **0.0** | **1.0** | **11.9** |
| 7206 | Sport & energy drinks | 0.0 | 0.0 | 0.0 |
| 7208 | Nutritional beverages | 0.0 | 0.0 | 0.3 |
| 7302 | Coffee | 0.0 | 0.0 | 0.0 |
| 7304 | Tea | 0.0 | 0.0 | 0.0 |
| 7702 | Tap water | 0.0 | 0.0 | 0.0 |
| 7704 | Bottled water | 0.0 | 0.0 | 0.0 |
| 7802 | Flavored or carbonated water | 0.0 | 0.0 | 0.0 |
| 7804 | Enhanced or fortified water | 0.0 | 0.1 | 0.3 |
| 8002 | Butter & animal fats | 0.0 | 0.0 | 0.0 |
| 8004 | Margarine | 0.0 | 0.0 | 0.0 |
| 8006 | Cream cheese, sour cream, whipped cream | 0.0 | 0.0 | 0.0 |
| 8008 | Cream & cream substitutes | 0.0 | 0.0 | 0.0 |
| 8010 | Mayonnaise | 0.0 | 0.0 | 0.0 |
| 8012 | Salad dressings & vegetable oils | 0.0 | 0.0 | 0.0 |
| 8402 | Tomato-based condiments | 0.0 | 0.0 | 0.1 |
| 8404 | Soy-based condiments | 0.0 | 0.0 | 0.0 |
| 8406 | Mustard & other condiments | 0.0 | 0.0 | 0.0 |
| 8408 | Olives, pickles, pickled vegetables | 0.0 | 0.0 | 0.0 |
| 8410 | Pasta sauces, tomato-based | 0.0 | 0.0 | 0.0 |
| 8412 | Dips, gravies, other sauces | 0.0 | 0.0 | 0.0 |
| 8802 | Sugars & honey | 0.0 | 0.0 | 0.0 |
| 8804 | Sugar substitutes | 0.0 | 0.0 | 0.0 |
| 8806 | Jams, syrups, toppings | 0.0 | 0.0 | 0.1 |
| **9002** | **Baby food: cereals** | **0.5** | **2.7** | **0.6** |
| **9004** | **Baby food: fruit** | **2.6** | **15.8** | **2.5** |
| **9006** | **Baby food: vegetable** | **0.5** | **2.0** | **0.4** |
| 9008 | Baby food: meat & dinners | 0.0 | 0.3 | 0.2 |
| 9010 | Baby food: yogurt | 0.0 | 0.9 | 0.3 |
| 9012 | Baby food: snacks & sweets | 0.1 | 0.8 | 0.5 |
| **9202** | **Baby juice** | **3.5** | **13.6** | **7.1** |
| 9204 | Baby water | 0.0 | 0.0 | 0.0 |
| **9402** | **Formula, ready-to-feed** | **2.3** | **2.4** | **0.9** |
| **9404** | **Formula, prepared from powder** | **61.9** | **38.5** | **2.3** |
| **9406** | **Formula, prepared from concentrate** | **5.9** | **3.5** | **0.1** |
| **9602** | **Human milk** | **21.9** | **6.3** | **1.4** |
| 9999 | Not included in a food category | 0.0 | 0.0 | 0.3 |
|  | Total | 100 | 100 | 100 |

^a^ Contributions from all minor WWEIA food categories are reported. Those in bold contribute ≥1% to daily intake in at least one age group.

**Table S11.** Food sources of vitamin D among U.S. infants and toddlers aged 0–23.9 months by age group: NHANES 2005-12 (*n* = 2740) ^a^.

| **WWEIA Minor Food category code** | **Food category** | **% of daily intake** | | |
| --- | --- | --- | --- | --- |
|  |  | **0–5.9 mo (*n* = 765)** | **6–11.9 mo (*n* = 854)** | **12–23.9 mo  (*n* = 1121)** |
| **1002** | **Milk, whole** | **0.0** | **6.5** | **58.0** |
| **1004** | **Milk, reduced fat** | **0.0** | **1.2** | **13.4** |
| **1006** | **Milk, low-fat** | **0.0** | **0.7** | **1.3** |
| **1008** | **Milk, non-fat** | **0.0** | **0.0** | **1.3** |
| **1202** | **Flavored milk, whole** | **0.0** | **0.1** | **1.7** |
| 1204 | Flavored milk, reduced fat | 0.0 | 0.0 | 0.8 |
| 1206 | Flavored milk, low-fat | 0.0 | 0.0 | 0.4 |
| 1208 | Flavored milk, non-fat | 0.0 | 0.0 | 0.0 |
| 1402 | Milk shakes & other dairy drinks | 0.0 | 0.0 | 0.0 |
| **1404** | **Milk substitutes** | **0.0** | **0.5** | **2.0** |
| **1602** | **Cheese** | **0.0** | **0.3** | **1.3** |
| 1604 | Cottage/ricotta cheese | 0.0 | 0.0 | 0.0 |
| 1802 | Yogurt, whole & reduced fat | 0.0 | 0.3 | 0.6 |
| 1804 | Yogurt, low-fat & non-fat | 0.0 | 0.3 | 0.9 |
| 2002 | Beef, excludes ground | 0.0 | 0.0 | 0.0 |
| 2004 | Ground beef | 0.0 | 0.0 | 0.0 |
| 2006 | Pork | 0.0 | 0.0 | 0.1 |
| 2008 | Lamb, goat, game | 0.0 | 0.0 | 0.0 |
| 2010 | Liver & organ meats | 0.0 | 0.0 | 0.0 |
| 2202 | Chicken, whole pieces | 0.0 | 0.0 | 0.1 |
| 2204 | Chicken patties, nuggets & tenders | 0.0 | 0.0 | 0.2 |
| 2206 | Turkey, duck, other poultry | 0.0 | 0.0 | 0.0 |
| 2402 | Fish | 0.0 | 0.0 | 0.5 |
| 2404 | Shellfish | 0.0 | 0.0 | 0.0 |
| **2502** | **Eggs & omelets** | **0.0** | **0.7** | **3.0** |
| 2602 | Cold cuts & cured meats | 0.0 | 0.0 | 0.2 |
| 2604 | Bacon | 0.0 | 0.0 | 0.0 |
| 2606 | Frankfurters | 0.0 | 0.1 | 0.5 |
| 2608 | Sausages | 0.0 | 0.1 | 0.3 |
| 2802 | Beans, peas, legumes | 0.0 | 0.0 | 0.0 |
| 2804 | Nuts & seeds | 0.0 | 0.0 | 0.0 |
| 2806 | Processed soy products | 0.0 | 0.0 | 0.0 |
| 3002 | Meat mixed dishes | 0.0 | 0.0 | 0.2 |
| 3004 | Poultry mixed dishes | 0.0 | 0.0 | 0.2 |
| 3006 | Seafood mixed dishes | 0.0 | 0.0 | 0.3 |
| 3202 | Rice mixed dishes | 0.0 | 0.0 | 0.0 |
| 3204 | Pasta mixed dishes, excludes macaroni & cheese | 0.0 | 0.1 | 0.3 |
| **3206** | **Macaroni & cheese** | **0.0** | **0.2** | **1.0** |
| 3208 | Turnovers & other grain-based items | 0.0 | 0.0 | 0.0 |
| 3402 | Fried rice & lo/chow mein | 0.0 | 0.0 | 0.0 |
| 3404 | Stir-fry & soy-based sauce mixtures | 0.0 | 0.0 | 0.0 |
| 3406 | Egg rolls, dumplings, sushi | 0.0 | 0.0 | 0.0 |
| 3502 | Burritos & tacos | 0.0 | 0.0 | 0.0 |
| 3504 | Nachos | 0.0 | 0.0 | 0.0 |
| 3506 | Other Mexican mixed dishes | 0.0 | 0.0 | 0.0 |
| 3602 | Pizza | 0.0 | 0.0 | 0.0 |
| 3702 | Burgers (single code) | 0.0 | 0.0 | 0.0 |
| 3704 | Chicken/turkey sandwiches (single code) | 0.0 | 0.0 | 0.0 |
| 3706 | Egg/breakfast sandwiches (single code) | 0.0 | 0.0 | 0.0 |
| 3708 | Other sandwiches (single code) | 0.0 | 0.0 | 0.1 |
| 3802 | Soups | 0.0 | 0.0 | 0.1 |
| 4002 | Rice | 0.0 | 0.0 | 0.0 |
| 4004 | Pasta, noodles, cooked grains | 0.0 | 0.0 | 0.0 |
| 4202 | Yeast breads | 0.0 | 0.0 | 0.0 |
| 4204 | Rolls & buns | 0.0 | 0.0 | 0.0 |
| 4206 | Bagels & English muffins | 0.0 | 0.0 | 0.0 |
| 4208 | Tortillas | 0.0 | 0.0 | 0.0 |
| 4402 | Biscuits, muffins, quick breads | 0.0 | 0.0 | 0.1 |
| 4404 | Pancakes, waffles, French toast | 0.0 | 0.0 | 0.1 |
| **4602** | **Ready-to-eat cereal, higher sugar (>21.2g/100g)** | **0.0** | **0.2** | **1.5** |
| **4604** | **Ready-to-eat cereal, lower sugar (≤21.2g/100g)** | **0.0** | **0.6** | **1.3** |
| 4802 | Oatmeal | 0.0 | 0.0 | 0.5 |
| 4804 | Grits & other cooked cereals | 0.0 | 0.1 | 0.3 |
| 5002 | Potato chips | 0.0 | 0.0 | 0.0 |
| 5004 | Tortilla, corn, other chips | 0.0 | 0.0 | 0.0 |
| 5006 | Popcorn | 0.0 | 0.0 | 0.0 |
| 5008 | Pretzels/snack mix | 0.0 | 0.0 | 0.0 |
| 5202 | Crackers, excludes saltines | 0.0 | 0.0 | 0.0 |
| 5204 | Saltine crackers | 0.0 | 0.0 | 0.0 |
| 5402 | Cereal bars | 0.0 | 0.0 | 0.1 |
| 5404 | Nutrition bars | 0.0 | 0.0 | 0.0 |
| 5502 | Cakes & pies | 0.0 | 0.0 | 0.0 |
| 5504 | Cookies & brownies | 0.0 | 0.0 | 0.0 |
| 5506 | Doughnuts, sweet rolls, pastries | 0.0 | 0.0 | 0.0 |
| 5702 | Candy containing chocolate | 0.0 | 0.0 | 0.0 |
| 5704 | Candy not containing chocolate | 0.0 | 0.0 | 0.0 |
| 5802 | Ice cream & frozen dairy desserts | 0.0 | 0.0 | 0.1 |
| 5804 | Pudding | 0.0 | 0.0 | 0.0 |
| 5806 | Gelatins, ices, sorbets | 0.0 | 0.0 | 0.0 |
| 6002 | Apples | 0.0 | 0.0 | 0.0 |
| 6004 | Bananas | 0.0 | 0.0 | 0.0 |
| 6006 | Grapes | 0.0 | 0.0 | 0.0 |
| 6008 | Peaches & nectarines | 0.0 | 0.0 | 0.0 |
| 6010 | Berries | 0.0 | 0.0 | 0.0 |
| 6012 | Citrus fruits | 0.0 | 0.0 | 0.0 |
| 6014 | Melons | 0.0 | 0.0 | 0.0 |
| 6016 | Dried fruits | 0.0 | 0.0 | 0.0 |
| 6018 | Other fruits & fruit salads | 0.0 | 0.0 | 0.0 |
| 6402 | Tomatoes | 0.0 | 0.0 | 0.0 |
| 6404 | Carrots | 0.0 | 0.0 | 0.0 |
| 6406 | Other red & orange vegetables | 0.0 | 0.0 | 0.0 |
| 6408 | Dark green vegetables, excludes lettuce | 0.0 | 0.0 | 0.0 |
| 6410 | Lettuce & lettuce salads | 0.0 | 0.0 | 0.0 |
| 6412 | String beans | 0.0 | 0.0 | 0.0 |
| 6414 | Onions | 0.0 | 0.0 | 0.0 |
| 6416 | Corn | 0.0 | 0.0 | 0.0 |
| 6418 | Other starchy vegetables | 0.0 | 0.0 | 0.0 |
| 6420 | Other vegetables & combinations | 0.0 | 0.0 | 0.0 |
| 6422 | Vegetable mixed dishes | 0.0 | 0.0 | 0.1 |
| 6802 | White potatoes, baked or boiled | 0.0 | 0.0 | 0.0 |
| 6804 | French fries & other fried white potatoes | 0.0 | 0.0 | 0.1 |
| 6806 | Mashed potatoes & white potato mixtures | 0.0 | 0.1 | 0.2 |
| **7002** | **Citrus juice** | **0.0** | **0.1** | **1.0** |
| 7004 | Apple juice | 0.0 | 0.0 | 0.0 |
| 7006 | Other fruit juice | 0.0 | 0.0 | 0.0 |
| 7008 | Vegetable juice | 0.0 | 0.0 | 0.0 |
| 7102 | Diet soft drinks | 0.0 | 0.0 | 0.0 |
| 7106 | Other diet drinks | 0.0 | 0.0 | 0.0 |
| 7202 | Soft drinks | 0.0 | 0.0 | 0.0 |
| 7204 | Fruit drinks | 0.0 | 0.0 | 0.2 |
| 7206 | Sport & energy drinks | 0.0 | 0.0 | 0.0 |
| 7208 | Nutritional beverages | 0.0 | 0.0 | 0.2 |
| 7302 | Coffee | 0.0 | 0.0 | 0.0 |
| 7304 | Tea | 0.0 | 0.0 | 0.0 |
| 7702 | Tap water | 0.0 | 0.0 | 0.0 |
| 7704 | Bottled water | 0.0 | 0.0 | 0.0 |
| 7802 | Flavored or carbonated water | 0.0 | 0.0 | 0.0 |
| 7804 | Enhanced or fortified water | 0.0 | 0.0 | 0.0 |
| 8002 | Butter & animal fats | 0.0 | 0.0 | 0.1 |
| 8004 | Margarine | 0.0 | 0.0 | 0.1 |
| 8006 | Cream cheese, sour cream, whipped cream | 0.0 | 0.0 | 0.0 |
| 8008 | Cream & cream substitutes | 0.0 | 0.0 | 0.0 |
| 8010 | Mayonnaise | 0.0 | 0.0 | 0.0 |
| 8012 | Salad dressings & vegetable oils | 0.0 | 0.0 | 0.0 |
| 8402 | Tomato-based condiments | 0.0 | 0.0 | 0.0 |
| 8404 | Soy-based condiments | 0.0 | 0.0 | 0.0 |
| 8406 | Mustard & other condiments | 0.0 | 0.0 | 0.0 |
| 8408 | Olives, pickles, pickled vegetables | 0.0 | 0.0 | 0.0 |
| 8410 | Pasta sauces, tomato-based | 0.0 | 0.0 | 0.0 |
| 8412 | Dips, gravies, other sauces | 0.0 | 0.0 | 0.0 |
| 8802 | Sugars & honey | 0.0 | 0.0 | 0.0 |
| 8804 | Sugar substitutes | 0.0 | 0.0 | 0.0 |
| 8806 | Jams, syrups, toppings | 0.0 | 0.0 | 0.0 |
| 9002 | Baby food: cereals | 0.0 | 0.0 | 0.0 |
| 9004 | Baby food: fruit | 0.0 | 0.0 | 0.0 |
| 9006 | Baby food: vegetable | 0.0 | 0.0 | 0.0 |
| 9008 | Baby food: meat & dinners | 0.0 | 0.3 | 0.0 |
| 9010 | Baby food: yogurt | 0.0 | 0.0 | 0.0 |
| 9012 | Baby food: snacks & sweets | 0.0 | 0.1 | 0.0 |
| 9202 | Baby juice | 0.0 | 0.0 | 0.0 |
| 9204 | Baby water | 0.0 | 0.0 | 0.0 |
| **9402** | **Formula, ready-to-feed** | **3.2** | **4.4** | **1.2** |
| **9404** | **Formula, prepared from powder** | **85.3** | **74.7** | **3.2** |
| **9406** | **Formula, prepared from concentrate** | **7.4** | **5.9** | **0.1** |
| **9602** | **Human milk** | **4.1** | **1.7** | **0.3** |
| 9999 | Not included in a food category | 0.0 | 0.0 | 0.1 |
|  | Total | 100 | 100 | 100 |

^a^ Contributions from all minor WWEIA food categories are reported. Those in bold contribute ≥1% to daily intake in at least one age group.

**Table S12.** Food sources of Vitamin E among U.S. infants and toddlers aged 0–23.9 months by age group: NHANES 2005-12 (*n* = 2740) ^a^.

| **WWEIA Minor Food category code** | **Food category** | **% of daily intake** | | |
| --- | --- | --- | --- | --- |
|  |  | **0–5.9 mo (*n* = 765)** | **6–11.9 mo (*n* = 854)** | **12–23.9 mo  (*n* = 1121)** |
| **1002** | **Milk, whole** | **0.0** | **0.3** | **6.5** |
| 1004 | Milk, reduced fat | 0.0 | 0.0 | 0.7 |
| 1006 | Milk, low-fat | 0.0 | 0.0 | 0.0 |
| 1008 | Milk, non-fat | 0.0 | 0.0 | 0.0 |
| 1202 | Flavored milk, whole | 0.0 | 0.0 | 0.2 |
| 1204 | Flavored milk, reduced fat | 0.0 | 0.0 | 0.1 |
| 1206 | Flavored milk, low-fat | 0.0 | 0.0 | 0.0 |
| 1208 | Flavored milk, non-fat | 0.0 | 0.0 | 0.0 |
| 1402 | Milk shakes & other dairy drinks | 0.0 | 0.0 | 0.0 |
| 1404 | Milk substitutes | 0.0 | 0.8 | 0.8 |
| 1602 | Cheese | 0.0 | 0.1 | 0.9 |
| 1604 | Cottage/ricotta cheese | 0.0 | 0.0 | 0.0 |
| 1802 | Yogurt, whole & reduced fat | 0.0 | 0.0 | 0.2 |
| 1804 | Yogurt, low-fat & non-fat | 0.0 | 0.0 | 0.1 |
| 2002 | Beef, excludes ground | 0.0 | 0.0 | 0.1 |
| 2004 | Ground beef | 0.0 | 0.0 | 0.2 |
| 2006 | Pork | 0.0 | 0.0 | 0.1 |
| 2008 | Lamb, goat, game | 0.0 | 0.0 | 0.0 |
| 2010 | Liver & organ meats | 0.0 | 0.0 | 0.0 |
| **2202** | **Chicken, whole pieces** | **0.0** | **0.1** | **1.4** |
| **2204** | **Chicken patties, nuggets & tenders** | **0.0** | **0.1** | **2.5** |
| 2206 | Turkey, duck, other poultry | 0.0 | 0.0 | 0.0 |
| 2402 | Fish | 0.0 | 0.0 | 0.5 |
| 2404 | Shellfish | 0.0 | 0.0 | 0.1 |
| **2502** | **Eggs & omelets** | **0.0** | **0.5** | **4.5** |
| 2602 | Cold cuts & cured meats | 0.0 | 0.0 | 0.2 |
| 2604 | Bacon | 0.0 | 0.0 | 0.1 |
| 2606 | Frankfurters | 0.0 | 0.0 | 0.3 |
| 2608 | Sausages | 0.0 | 0.0 | 0.3 |
| 2802 | Beans, peas, legumes | 0.0 | 0.1 | 0.8 |
| **2804** | **Nuts & seeds** | **0.0** | **0.1** | **5.0** |
| 2806 | Processed soy products | 0.0 | 0.1 | 0.1 |
| 3002 | Meat mixed dishes | 0.0 | 0.1 | 1.1 |
| **3004** | **Poultry mixed dishes** | **0.0** | **0.1** | **1.2** |
| 3006 | Seafood mixed dishes | 0.0 | 0.0 | 0.4 |
| 3202 | Rice mixed dishes | 0.0 | 0.1 | 0.8 |
| **3204** | **Pasta mixed dishes, excludes macaroni & cheese** | **0.0** | **1.0** | **6.2** |
| **3206** | **Macaroni & cheese** | **0.0** | **0.2** | **1.5** |
| 3208 | Turnovers & other grain-based items | 0.0 | 0.0 | 0.1 |
| 3402 | Fried rice & lo/chow mein | 0.0 | 0.0 | 0.3 |
| 3404 | Stir-fry & soy-based sauce mixtures | 0.0 | 0.0 | 0.2 |
| 3406 | Egg rolls, dumplings, sushi | 0.0 | 0.0 | 0.0 |
| 3502 | Burritos & tacos | 0.0 | 0.0 | 0.2 |
| 3504 | Nachos | 0.0 | 0.0 | 0.0 |
| 3506 | Other Mexican mixed dishes | 0.0 | 0.0 | 0.3 |
| **3602** | **Pizza** | **0.0** | **0.1** | **1.6** |
| 3702 | Burgers (single code) | 0.0 | 0.0 | 0.0 |
| 3704 | Chicken/turkey sandwiches (single code) | 0.0 | 0.0 | 0.1 |
| 3706 | Egg/breakfast sandwiches (single code) | 0.0 | 0.0 | 0.0 |
| 3708 | Other sandwiches (single code) | 0.0 | 0.0 | 0.2 |
| **3802** | **Soups** | **0.0** | **0.6** | **2.1** |
| 4002 | Rice | 0.0 | 0.0 | 0.2 |
| 4004 | Pasta, noodles, cooked grains | 0.0 | 0.0 | 0.1 |
| **4202** | **Yeast breads** | **0.0** | **0.1** | **1.4** |
| 4204 | Rolls & buns | 0.0 | 0.0 | 0.5 |
| 4206 | Bagels & English muffins | 0.0 | 0.0 | 0.1 |
| 4208 | Tortillas | 0.0 | 0.0 | 0.2 |
| 4402 | Biscuits, muffins, quick breads | 0.0 | 0.1 | 0.7 |
| 4404 | Pancakes, waffles, French toast | 0.0 | 0.1 | 0.6 |
| 4602 | Ready-to-eat cereal, higher sugar (>21.2g/100g) | 0.0 | 0.0 | 0.9 |
| **4604** | **Ready-to-eat cereal, lower sugar (≤21.2g/100g)** | **0.0** | **0.5** | **2.0** |
| 4802 | Oatmeal | 0.0 | 0.0 | 0.5 |
| 4804 | Grits & other cooked cereals | 0.0 | 0.0 | 0.3 |
| **5002** | **Potato chips** | **0.0** | **0.0** | **2.0** |
| **5004** | **Tortilla, corn, other chips** | **0.0** | **0.4** | **2.9** |
| 5006 | Popcorn | 0.0 | 0.0 | 0.2 |
| 5008 | Pretzels/snack mix | 0.0 | 0.0 | 0.2 |
| **5202** | **Crackers, excludes saltines** | **0.0** | **0.4** | **3.3** |
| 5204 | Saltine crackers | 0.0 | 0.0 | 0.2 |
| 5402 | Cereal bars | 0.0 | 0.0 | 0.4 |
| 5404 | Nutrition bars | 0.0 | 0.0 | 0.0 |
| 5502 | Cakes & pies | 0.0 | 0.0 | 0.6 |
| **5504** | **Cookies & brownies** | **0.0** | **0.3** | **2.0** |
| 5506 | Doughnuts, sweet rolls, pastries | 0.0 | 0.0 | 0.7 |
| 5702 | Candy containing chocolate | 0.0 | 0.0 | 0.1 |
| 5704 | Candy not containing chocolate | 0.0 | 0.0 | 0.3 |
| 5802 | Ice cream & frozen dairy desserts | 0.0 | 0.0 | 0.3 |
| 5804 | Pudding | 0.0 | 0.0 | 0.1 |
| 5806 | Gelatins, ices, sorbets | 0.0 | 0.0 | 0.0 |
| **6002** | **Apples** | **0.0** | **0.2** | **1.0** |
| 6004 | Bananas | 0.0 | 0.2 | 0.6 |
| 6006 | Grapes | 0.0 | 0.0 | 0.3 |
| **6008** | **Peaches & nectarines** | **0.0** | **0.3** | **1.1** |
| 6010 | Berries | 0.0 | 0.1 | 0.6 |
| 6012 | Citrus fruits | 0.0 | 0.0 | 0.4 |
| 6014 | Melons | 0.0 | 0.0 | 0.1 |
| 6016 | Dried fruits | 0.0 | 0.1 | 0.2 |
| **6018** | **Other fruits & fruit salads** | **0.0** | **0.2** | **1.2** |
| 6402 | Tomatoes | 0.0 | 0.0 | 0.3 |
| 6404 | Carrots | 0.0 | 0.1 | 0.5 |
| 6406 | Other red & orange vegetables | 0.0 | 0.1 | 0.2 |
| **6408** | **Dark green vegetables, excludes lettuce** | **0.0** | **0.1** | **1.1** |
| 6410 | Lettuce & lettuce salads | 0.0 | 0.0 | 0.0 |
| 6412 | String beans | 0.0 | 0.0 | 0.3 |
| 6414 | Onions | 0.0 | 0.0 | 0.0 |
| 6416 | Corn | 0.0 | 0.0 | 0.1 |
| 6418 | Other starchy vegetables | 0.0 | 0.1 | 0.2 |
| 6420 | Other vegetables & combinations | 0.0 | 0.2 | 0.7 |
| 6422 | Vegetable mixed dishes | 0.0 | 0.0 | 0.4 |
| 6802 | White potatoes, baked or boiled | 0.0 | 0.0 | 0.1 |
| **6804** | **French fries & other fried white potatoes** | **0.0** | **0.1** | **1.2** |
| 6806 | Mashed potatoes & white potato mixtures | 0.0 | 0.2 | 0.6 |
| **7002** | **Citrus juice** | **0.0** | **0.0** | **1.1** |
| 7004 | Apple juice | 0.0 | 0.0 | 0.2 |
| 7006 | Other fruit juice | 0.0 | 0.0 | 0.8 |
| 7008 | Vegetable juice | 0.0 | 0.0 | 0.2 |
| 7102 | Diet soft drinks | 0.0 | 0.0 | 0.0 |
| 7106 | Other diet drinks | 0.0 | 0.0 | 0.0 |
| 7202 | Soft drinks | 0.0 | 0.0 | 0.0 |
| **7204** | **Fruit drinks** | **0.0** | **0.1** | **1.9** |
| 7206 | Sport & energy drinks | 0.0 | 0.0 | 0.0 |
| 7208 | Nutritional beverages | 0.0 | 0.0 | 0.8 |
| 7302 | Coffee | 0.0 | 0.0 | 0.0 |
| 7304 | Tea | 0.0 | 0.0 | 0.0 |
| 7702 | Tap water | 0.0 | 0.0 | 0.0 |
| 7704 | Bottled water | 0.0 | 0.0 | 0.0 |
| 7802 | Flavored or carbonated water | 0.0 | 0.0 | 0.0 |
| **7804** | **Enhanced or fortified water** | **0.0** | **0.1** | **1.2** |
| 8002 | Butter & animal fats | 0.0 | 0.0 | 0.2 |
| **8004** | **Margarine** | **0.0** | **0.2** | **1.3** |
| 8006 | Cream cheese, sour cream, whipped cream | 0.0 | 0.0 | 0.0 |
| 8008 | Cream & cream substitutes | 0.0 | 0.0 | 0.0 |
| 8010 | Mayonnaise | 0.0 | 0.0 | 0.1 |
| 8012 | Salad dressings & vegetable oils | 0.0 | 0.0 | 0.4 |
| 8402 | Tomato-based condiments | 0.0 | 0.0 | 0.2 |
| 8404 | Soy-based condiments | 0.0 | 0.0 | 0.0 |
| 8406 | Mustard & other condiments | 0.0 | 0.0 | 0.0 |
| 8408 | Olives, pickles, pickled vegetables | 0.0 | 0.0 | 0.1 |
| 8410 | Pasta sauces, tomato-based | 0.0 | 0.0 | 0.4 |
| 8412 | Dips, gravies, other sauces | 0.0 | 0.0 | 0.0 |
| 8802 | Sugars & honey | 0.0 | 0.0 | 0.0 |
| 8804 | Sugar substitutes | 0.0 | 0.0 | 0.0 |
| 8806 | Jams, syrups, toppings | 0.0 | 0.0 | 0.0 |
| **9002** | **Baby food: cereals** | **3.6** | **7.9** | **2.4** |
| **9004** | **Baby food: fruit** | **0.4** | **3.8** | **0.9** |
| **9006** | **Baby food: vegetable** | **0.6** | **2.6** | **0.5** |
| **9008** | **Baby food: meat & dinners** | **0.0** | **1.3** | **0.9** |
| 9010 | Baby food: yogurt | 0.0 | 0.3 | 0.2 |
| 9012 | Baby food: snacks & sweets | 0.0 | 0.8 | 0.8 |
| **9202** | **Baby juice** | **0.2** | **1.1** | **0.9** |
| 9204 | Baby water | 0.0 | 0.0 | 0.0 |
| **9402** | **Formula, ready-to-feed** | **4.1** | **5.0** | **2.8** |
| **9404** | **Formula, prepared from powder** | **80.5** | **61.4** | **5.4** |
| **9406** | **Formula, prepared from concentrate** | **6.4** | **4.7** | **0.2** |
| **9602** | **Human milk** | **4.0** | **1.4** | **0.5** |
| 9999 | Not included in a food category | 0.0 | 0.0 | 0.6 |
|  | Total | 100 | 100 | 100 |

^a^ Contributions from all minor WWEIA food categories are reported. Those in bold contribute ≥1% to daily intake in at least one age group.

**Table S13.** Food sources of potassium among U.S. infants and toddlers aged 0–23.9 months by age group: NHANES 2005-12 (*n* = 2740) ^a^.

| **WWEIA Minor Food category code** | **Food category** | **% of daily intake** | | |
| --- | --- | --- | --- | --- |
|  |  | **0–5.9 mo (*n* = 765)** | **6–11.9 mo (*n* = 854)** | **12–23.9 mo  (*n* = 1121)** |
| **1002** | **Milk, whole** | **0.0** | **4.3** | **26.3** |
| **1004** | **Milk, reduced fat** | **0.0** | **0.9** | **7.0** |
| 1006 | Milk, lowfat | 0.0 | 0.5 | 0.7 |
| 1008 | Milk, nonfat | 0.0 | 0.0 | 0.8 |
| 1202 | Flavored milk, whole | 0.0 | 0.0 | 0.9 |
| 1204 | Flavored milk, reduced fat | 0.0 | 0.0 | 0.4 |
| 1206 | Flavored milk, lowfat | 0.0 | 0.0 | 0.3 |
| 1208 | Flavored milk, nonfat | 0.0 | 0.0 | 0.0 |
| 1402 | Milk shakes and other dairy drinks | 0.0 | 0.0 | 0.0 |
| **1404** | **Milk substitutes** | **0.0** | **0.3** | **1.1** |
| 1602 | Cheese | 0.0 | 0.2 | 0.8 |
| 1604 | Cottage/ricotta cheese | 0.0 | 0.0 | 0.1 |
| 1802 | Yogurt, whole and reduced fat | 0.0 | 0.7 | 0.9 |
| 1804 | Yogurt, lowfat and nonfat | 0.0 | 0.7 | 1.3 |
| 2002 | Beef, excludes ground | 0.0 | 0.1 | 0.2 |
| 2004 | Ground beef | 0.0 | 0.2 | 0.3 |
| 2006 | Pork | 0.0 | 0.0 | 0.2 |
| 2008 | Lamb, goat, game | 0.0 | 0.0 | 0.0 |
| 2010 | Liver and organ meats | 0.0 | 0.0 | 0.0 |
| **2202** | **Chicken, whole pieces** | **0.0** | **0.3** | **1.4** |
| **2204** | **Chicken patties, nuggets and tenders** | **0.0** | **0.1** | **1.2** |
| 2206 | Turkey, duck, other poultry | 0.0 | 0.1 | 0.1 |
| 2402 | Fish | 0.0 | 0.0 | 0.2 |
| 2404 | Shellfish | 0.0 | 0.0 | 0.0 |
| **2502** | **Eggs and omelets** | **0.0** | **0.4** | **1.3** |
| 2602 | Cold cuts and cured meats | 0.0 | 0.2 | 0.8 |
| 2604 | Bacon | 0.0 | 0.0 | 0.1 |
| 2606 | Frankfurters | 0.0 | 0.2 | 0.6 |
| 2608 | Sausages | 0.0 | 0.2 | 0.4 |
| **2802** | **Beans, peas, legumes** | **0.0** | **1.1** | **1.1** |
| 2804 | Nuts and seeds | 0.0 | 0.1 | 0.6 |
| 2806 | Processed soy products | 0.0 | 0.0 | 0.1 |
| **3002** | **Meat mixed dishes** | **0.0** | **0.5** | **1.2** |
| 3004 | Poultry mixed dishes | 0.0 | 0.3 | 0.7 |
| 3006 | Seafood mixed dishes | 0.0 | 0.0 | 0.2 |
| 3202 | Rice mixed dishes | 0.0 | 0.2 | 0.5 |
| **3204** | **Pasta mixed dishes, excludes macaroni and cheese** | **0.0** | **1.2** | **2.2** |
| 3206 | Macaroni and cheese | 0.0 | 0.3 | 0.9 |
| 3208 | Turnovers and other grain-based items | 0.0 | 0.0 | 0.1 |
| 3402 | Fried rice and lo/chow mein | 0.0 | 0.0 | 0.2 |
| 3404 | Stir-fry and soy-based sauce mixtures | 0.0 | 0.1 | 0.1 |
| 3406 | Egg rolls, dumplings, sushi | 0.0 | 0.0 | 0.0 |
| 3502 | Burritos and tacos | 0.0 | 0.0 | 0.2 |
| 3504 | Nachos | 0.0 | 0.0 | 0.0 |
| 3506 | Other Mexican mixed dishes | 0.0 | 0.0 | 0.2 |
| 3602 | Pizza | 0.0 | 0.1 | 0.6 |
| 3702 | Burgers (single code) | 0.0 | 0.1 | 0.1 |
| 3704 | Chicken/turkey sandwiches (single code) | 0.0 | 0.0 | 0.1 |
| 3706 | Egg/breakfast sandwiches (single code) | 0.0 | 0.0 | 0.0 |
| 3708 | Other sandwiches (single code) | 0.0 | 0.0 | 0.2 |
| **3802** | **Soups** | **0.0** | **1.7** | **1.9** |
| 4002 | Rice | 0.0 | 0.1 | 0.1 |
| 4004 | Pasta, noodles, cooked grains | 0.0 | 0.0 | 0.0 |
| 4202 | Yeast breads | 0.0 | 0.3 | 0.9 |
| 4204 | Rolls and buns | 0.0 | 0.0 | 0.2 |
| 4206 | Bagels and English muffins | 0.0 | 0.0 | 0.1 |
| 4208 | Tortillas | 0.0 | 0.1 | 0.2 |
| 4402 | Biscuits, muffins, quick breads | 0.0 | 0.1 | 0.2 |
| 4404 | Pancakes, waffles, French toast | 0.0 | 0.1 | 0.3 |
| 4602 | Ready-to-eat cereal, higher sugar (>21.2g/100g) | 0.0 | 0.1 | 0.5 |
| 4604 | Ready-to-eat cereal, lower sugar (≤21.2g/100g) | 0.0 | 0.5 | 0.8 |
| 4802 | Oatmeal | 0.0 | 0.2 | 0.9 |
| 4804 | Grits and other cooked cereals | 0.0 | 0.1 | 0.2 |
| 5002 | Potato chips | 0.0 | 0.1 | 1.0 |
| 5004 | Tortilla, corn, other chips | 0.0 | 0.1 | 0.3 |
| 5006 | Popcorn | 0.0 | 0.0 | 0.0 |
| 5008 | Pretzels/snack mix | 0.0 | 0.0 | 0.1 |
| 5202 | Crackers, excludes saltines | 0.0 | 0.1 | 0.4 |
| 5204 | Saltine crackers | 0.0 | 0.1 | 0.1 |
| 5402 | Cereal bars | 0.0 | 0.0 | 0.2 |
| 5404 | Nutrition bars | 0.0 | 0.0 | 0.0 |
| 5502 | Cakes and pies | 0.0 | 0.1 | 0.1 |
| 5504 | Cookies and brownies | 0.0 | 0.5 | 0.6 |
| 5506 | Doughnuts, sweet rolls, pastries | 0.0 | 0.0 | 0.1 |
| 5702 | Candy containing chocolate | 0.0 | 0.0 | 0.1 |
| 5704 | Candy not containing chocolate | 0.0 | 0.1 | 0.2 |
| 5802 | Ice cream and frozen dairy desserts | 0.0 | 0.1 | 0.5 |
| 5804 | Pudding | 0.0 | 0.1 | 0.2 |
| 5806 | Gelatins, ices, sorbets | 0.0 | 0.0 | 0.1 |
| **6002** | **Apples** | **0.0** | **0.6** | **1.0** |
| **6004** | **Bananas** | **0.1** | **3.3** | **4.3** |
| 6006 | Grapes | 0.0 | 0.1 | 0.7 |
| 6008 | Peaches and nectarines | 0.0 | 0.2 | 0.4 |
| 6010 | Berries | 0.0 | 0.1 | 0.4 |
| 6012 | Citrus fruits | 0.0 | 0.2 | 0.7 |
| 6014 | Melons | 0.0 | 0.2 | 0.5 |
| 6016 | Dried fruits | 0.0 | 0.2 | 0.5 |
| 6018 | Other fruits and fruit salads | 0.0 | 0.3 | 0.8 |
| 6402 | Tomatoes | 0.0 | 0.0 | 0.3 |
| 6404 | Carrots | 0.0 | 0.2 | 0.3 |
| 6406 | Other red and orange vegetables | 0.0 | 0.5 | 0.3 |
| 6408 | Dark green vegetables, excludes lettuce | 0.0 | 0.2 | 0.4 |
| 6410 | Lettuce and lettuce salads | 0.0 | 0.0 | 0.1 |
| 6412 | String beans | 0.0 | 0.1 | 0.3 |
| 6414 | Onions | 0.0 | 0.0 | 0.0 |
| 6416 | Corn | 0.0 | 0.1 | 0.3 |
| 6418 | Other starchy vegetables | 0.0 | 0.4 | 0.3 |
| 6420 | Other vegetables and combinations | 0.0 | 0.6 | 0.5 |
| 6422 | Vegetable mixed dishes | 0.0 | 0.0 | 0.2 |
| 6802 | White potatoes, baked or boiled | 0.0 | 0.1 | 0.4 |
| **6804** | **French fries and other fried white potatoes** | **0.0** | **0.4** | **1.6** |
| 6806 | Mashed potatoes and white potato mixtures | 0.1 | 0.8 | 0.9 |
| **7002** | **Citrus juice** | **0.0** | **0.3** | **1.9** |
| **7004** | **Apple juice** | **0.1** | **1.2** | **4.1** |
| **7006** | **Other fruit juice** | **0.2** | **1.2** | **3.3** |
| 7008 | Vegetable juice | 0.0 | 0.0 | 0.1 |
| 7102 | Diet soft drinks | 0.0 | 0.0 | 0.0 |
| 7106 | Other diet drinks | 0.0 | 0.0 | 0.0 |
| 7202 | Soft drinks | 0.0 | 0.0 | 0.0 |
| **7204** | **Fruit drinks** | **0.0** | **0.2** | **1.5** |
| 7206 | Sport and energy drinks | 0.3 | 0.2 | 0.4 |
| 7208 | Nutritional beverages | 0.0 | 0.0 | 0.2 |
| 7302 | Coffee | 0.0 | 0.0 | 0.0 |
| 7304 | Tea | 0.0 | 0.1 | 0.3 |
| 7702 | Tap water | 0.0 | 0.0 | 0.0 |
| 7704 | Bottled water | 0.0 | 0.0 | 0.0 |
| 7802 | Flavored or carbonated water | 0.0 | 0.0 | 0.0 |
| 7804 | Enhanced or fortified water | 0.0 | 0.0 | 0.0 |
| 8002 | Butter and animal fats | 0.0 | 0.0 | 0.0 |
| 8004 | Margarine | 0.0 | 0.0 | 0.0 |
| 8006 | Cream cheese, sour cream, whipped cream | 0.0 | 0.0 | 0.0 |
| 8008 | Cream and cream substitutes | 0.0 | 0.0 | 0.0 |
| 8010 | Mayonnaise | 0.0 | 0.0 | 0.0 |
| 8012 | Salad dressings and vegetable oils | 0.0 | 0.0 | 0.0 |
| 8402 | Tomato-based condiments | 0.0 | 0.0 | 0.1 |
| 8404 | Soy-based condiments | 0.0 | 0.0 | 0.0 |
| 8406 | Mustard and other condiments | 0.0 | 0.0 | 0.0 |
| 8408 | Olives, pickles, pickled vegetables | 0.0 | 0.0 | 0.0 |
| 8410 | Pasta sauces, tomato-based | 0.0 | 0.0 | 0.1 |
| 8412 | Dips, gravies, other sauces | 0.0 | 0.0 | 0.0 |
| 8802 | Sugars and honey | 0.0 | 0.0 | 0.0 |
| 8804 | Sugar substitutes | 0.0 | 0.0 | 0.0 |
| 8806 | Jams, syrups, toppings | 0.0 | 0.0 | 0.1 |
| **9002** | **Baby food: cereals** | **2.6** | **4.6** | **0.5** |
| **9004** | **Baby food: fruit** | **1.3** | **7.4** | **0.6** |
| **9006** | **Baby food: vegetable** | **2.2** | **7.5** | **0.5** |
| **9008** | **Baby food: meat and dinners** | **0.2** | **3.0** | **0.7** |
| 9010 | Baby food: yogurt | 0.0 | 0.6 | 0.1 |
| 9012 | Baby food: snacks and sweets | 0.0 | 0.7 | 0.2 |
| **9202** | **Baby juice** | **0.7** | **2.3** | **0.5** |
| 9204 | Baby water | 0.0 | 0.0 | 0.0 |
| **9402** | **Formula, ready-to-feed** | **2.4** | **2.1** | **0.4** |
| **9404** | **Formula, prepared from powder** | **62.2** | **33.2** | **1.0** |
| **9406** | **Formula, prepared from concentrate** | **5.6** | **2.8** | **0.0** |
| **9602** | **Human milk** | **21.8** | **5.4** | **0.6** |
| 9999 | Not included in a food category | 0.0 | 0.0 | 0.2 |
|  | Total | 100 | 100 | 100 |

^a^ Contributions from all minor WWEIA food categories are reported. Those in bold contribute ≥1% to daily intake in at least one age group

**Table S14.** Food sources of calcium among U.S. infants and toddlers aged 0–23.9 months by age group: NHANES 2005-12 (*n* = 2740) ^a^.

| **WWEIA Minor Food category code** | **Food category** | **% of daily intake** | | |
| --- | --- | --- | --- | --- |
|  |  | **0–5.9 mo (*n* = 765)** | **6–11.9 mo (*n* = 854)** | **12–23.9 mo  (*n* = 1121)** |
| **1002** | **Milk, whole** | **0.0** | **6.2** | **40.6** |
| **1004** | **Milk, reduced fat** | **0.0** | **1.3** | **10.8** |
| **1006** | **Milk, low-fat** | **0.0** | **0.7** | **1.1** |
| **1008** | **Milk, non-fat** | **0.0** | **0.0** | **1.2** |
| **1202** | **Flavored milk, whole** | **0.0** | **0.1** | **1.3** |
| 1204 | Flavored milk, reduced fat | 0.0 | 0.0 | 0.6 |
| 1206 | Flavored milk, low-fat | 0.0 | 0.0 | 0.4 |
| 1208 | Flavored milk, non-fat | 0.0 | 0.0 | 0.0 |
| 1402 | Milk shakes & other dairy drinks | 0.0 | 0.0 | 0.0 |
| **1404** | **Milk substitutes** | **0.0** | **0.9** | **1.9** |
| **1602** | **Cheese** | **0.0** | **2.1** | **6.8** |
| **1604** | **Cottage/ricotta cheese** | **0.0** | **0.0** | **0.1** |
| **1802** | **Yogurt, whole & reduced fat** | **0.0** | **1.0** | **1.2** |
| **1804** | **Yogurt, low-fat & non-fat** | **0.0** | **0.9** | **1.8** |
| 2002 | Beef, excludes ground | 0.0 | 0.0 | 0.0 |
| 2004 | Ground beef | 0.0 | 0.0 | 0.0 |
| 2006 | Pork | 0.0 | 0.0 | 0.0 |
| 2008 | Lamb, goat, game | 0.0 | 0.0 | 0.0 |
| 2010 | Liver & organ meats | 0.0 | 0.0 | 0.0 |
| 2202 | Chicken, whole pieces | 0.0 | 0.0 | 0.1 |
| 2204 | Chicken patties, nuggets & tenders | 0.0 | 0.0 | 0.2 |
| 2206 | Turkey, duck, other poultry | 0.0 | 0.0 | 0.0 |
| 2402 | Fish | 0.0 | 0.0 | 0.0 |
| 2404 | Shellfish | 0.0 | 0.0 | 0.0 |
| **2502** | **Eggs & omelets** | **0.0** | **0.4** | **1.3** |
| 2602 | Cold cuts & cured meats | 0.0 | 0.0 | 0.1 |
| 2604 | Bacon | 0.0 | 0.0 | 0.0 |
| 2606 | Frankfurters | 0.0 | 0.0 | 0.2 |
| 2608 | Sausages | 0.0 | 0.0 | 0.1 |
| 2802 | Beans, peas, legumes | 0.0 | 0.3 | 0.2 |
| 2804 | Nuts & seeds | 0.0 | 0.0 | 0.1 |
| 2806 | Processed soy products | 0.0 | 0.0 | 0.1 |
| 3002 | Meat mixed dishes | 0.0 | 0.1 | 0.3 |
| 3004 | Poultry mixed dishes | 0.0 | 0.0 | 0.3 |
| 3006 | Seafood mixed dishes | 0.0 | 0.0 | 0.1 |
| 3202 | Rice mixed dishes | 0.0 | 0.1 | 0.2 |
| 3204 | Pasta mixed dishes, excludes macaroni & cheese | 0.0 | 0.4 | 0.8 |
| **3206** | **Macaroni & cheese** | **0.0** | **0.6** | **1.8** |
| 3208 | Turnovers & other grain-based items | 0.0 | 0.0 | 0.1 |
| 3402 | Fried rice & lo/chow mein | 0.0 | 0.0 | 0.0 |
| 3404 | Stir-fry & soy-based sauce mixtures | 0.0 | 0.0 | 0.0 |
| 3406 | Egg rolls, dumplings, sushi | 0.0 | 0.0 | 0.0 |
| 3502 | Burritos & tacos | 0.0 | 0.0 | 0.1 |
| 3504 | Nachos | 0.0 | 0.0 | 0.0 |
| 3506 | Other Mexican mixed dishes | 0.0 | 0.1 | 0.4 |
| **3602** | **Pizza** | **0.0** | **0.1** | **1.2** |
| 3702 | Burgers (single code) | 0.0 | 0.1 | 0.2 |
| 3704 | Chicken/turkey sandwiches (single code) | 0.0 | 0.0 | 0.1 |
| 3706 | Egg/breakfast sandwiches (single code) | 0.0 | 0.0 | 0.0 |
| 3708 | Other sandwiches (single code) | 0.0 | 0.0 | 0.2 |
| 3802 | Soups | 0.0 | 0.3 | 0.4 |
| 4002 | Rice | 0.0 | 0.0 | 0.1 |
| 4004 | Pasta, noodles, cooked grains | 0.0 | 0.0 | 0.0 |
| **4202** | **Yeast breads** | **0.0** | **0.5** | **1.5** |
| 4204 | Rolls & buns | 0.0 | 0.0 | 0.3 |
| 4206 | Bagels & English muffins | 0.0 | 0.0 | 0.1 |
| 4208 | Tortillas | 0.0 | 0.1 | 0.2 |
| 4402 | Biscuits, muffins, quick breads | 0.0 | 0.1 | 0.2 |
| 4404 | Pancakes, waffles, French toast | 0.0 | 0.2 | 0.7 |
| 4602 | Ready-to-eat cereal, higher sugar (>21.2g/100g) | 0.0 | 0.1 | 0.7 |
| **4604** | **Ready-to-eat cereal, lower sugar (≤21.2g/100g)** | **0.0** | **0.6** | **1.1** |
| **4802** | **Oatmeal** | **0.0** | **0.3** | **1.2** |
| 4804 | Grits & other cooked cereals | 0.0 | 0.2 | 0.4 |
| 5002 | Potato chips | 0.0 | 0.0 | 0.0 |
| 5004 | Tortilla, corn, other chips | 0.0 | 0.1 | 0.3 |
| 5006 | Popcorn | 0.0 | 0.0 | 0.0 |
| 5008 | Pretzels/snack mix | 0.0 | 0.0 | 0.1 |
| 5202 | Crackers, excludes saltines | 0.0 | 0.2 | 0.6 |
| 5204 | Saltine crackers | 0.0 | 0.0 | 0.0 |
| 5402 | Cereal bars | 0.0 | 0.1 | 0.3 |
| 5404 | Nutrition bars | 0.0 | 0.0 | 0.0 |
| 5502 | Cakes & pies | 0.0 | 0.0 | 0.2 |
| 5504 | Cookies & brownies | 0.0 | 0.2 | 0.3 |
| 5506 | Doughnuts, sweet rolls, pastries | 0.0 | 0.0 | 0.1 |
| 5702 | Candy containing chocolate | 0.0 | 0.0 | 0.1 |
| 5704 | Candy not containing chocolate | 0.0 | 0.0 | 0.1 |
| 5802 | Ice cream & frozen dairy desserts | 0.0 | 0.1 | 0.6 |
| 5804 | Pudding | 0.0 | 0.1 | 0.1 |
| 5806 | Gelatins, ices, sorbets | 0.0 | 0.0 | 0.0 |
| 6002 | Apples | 0.0 | 0.1 | 0.1 |
| 6004 | Bananas | 0.0 | 0.1 | 0.1 |
| 6006 | Grapes | 0.0 | 0.0 | 0.1 |
| 6008 | Peaches & nectarines | 0.0 | 0.0 | 0.0 |
| 6010 | Berries | 0.0 | 0.0 | 0.1 |
| 6012 | Citrus fruits | 0.0 | 0.1 | 0.2 |
| 6014 | Melons | 0.0 | 0.0 | 0.0 |
| 6016 | Dried fruits | 0.0 | 0.0 | 0.1 |
| 6018 | Other fruits & fruit salads | 0.0 | 0.0 | 0.1 |
| 6402 | Tomatoes | 0.0 | 0.0 | 0.0 |
| 6404 | Carrots | 0.0 | 0.0 | 0.1 |
| 6406 | Other red & orange vegetables | 0.0 | 0.1 | 0.0 |
| 6408 | Dark green vegetables, excludes lettuce | 0.0 | 0.0 | 0.1 |
| 6410 | Lettuce & lettuce salads | 0.0 | 0.0 | 0.0 |
| 6412 | String beans | 0.0 | 0.1 | 0.2 |
| 6414 | Onions | 0.0 | 0.0 | 0.0 |
| 6416 | Corn | 0.0 | 0.0 | 0.0 |
| 6418 | Other starchy vegetables | 0.0 | 0.0 | 0.0 |
| 6420 | Other vegetables & combinations | 0.0 | 0.1 | 0.1 |
| 6422 | Vegetable mixed dishes | 0.0 | 0.0 | 0.1 |
| 6802 | White potatoes, baked or boiled | 0.0 | 0.0 | 0.0 |
| 6804 | French fries & other fried white potatoes | 0.0 | 0.0 | 0.2 |
| 6806 | Mashed potatoes & white potato mixtures | 0.0 | 0.1 | 0.2 |
| **7002** | **Citrus juice** | **0.0** | **0.2** | **1.9** |
| 7004 | Apple juice | 0.0 | 0.1 | 0.6 |
| **7006** | **Other fruit juice** | **0.0** | **0.3** | **1.2** |
| 7008 | Vegetable juice | 0.0 | 0.0 | 0.0 |
| 7102 | Diet soft drinks | 0.0 | 0.0 | 0.0 |
| 7106 | Other diet drinks | 0.0 | 0.0 | 0.1 |
| 7202 | Soft drinks | 0.0 | 0.0 | 0.0 |
| 7204 | Fruit drinks | 0.0 | 0.1 | 0.7 |
| 7206 | Sport & energy drinks | 0.0 | 0.1 | 0.1 |
| 7208 | Nutritional beverages | 0.0 | 0.0 | 0.2 |
| 7302 | Coffee | 0.0 | 0.0 | 0.0 |
| 7304 | Tea | 0.0 | 0.0 | 0.0 |
| 7702 | Tap water | 0.0 | 0.3 | 0.4 |
| 7704 | Bottled water | 0.1 | 0.4 | 0.8 |
| 7802 | Flavored or carbonated water | 0.0 | 0.0 | 0.0 |
| 7804 | Enhanced or fortified water | 0.0 | 0.0 | 0.0 |
| 8002 | Butter & animal fats | 0.0 | 0.0 | 0.0 |
| 8004 | Margarine | 0.0 | 0.0 | 0.0 |
| 8006 | Cream cheese, sour cream, whipped cream | 0.0 | 0.0 | 0.0 |
| 8008 | Cream & cream substitutes | 0.0 | 0.0 | 0.0 |
| 8010 | Mayonnaise | 0.0 | 0.0 | 0.0 |
| 8012 | Salad dressings & vegetable oils | 0.0 | 0.0 | 0.0 |
| 8402 | Tomato-based condiments | 0.0 | 0.0 | 0.0 |
| 8404 | Soy-based condiments | 0.0 | 0.0 | 0.0 |
| 8406 | Mustard & other condiments | 0.0 | 0.0 | 0.0 |
| 8408 | Olives, pickles, pickled vegetables | 0.0 | 0.0 | 0.0 |
| 8410 | Pasta sauces, tomato-based | 0.0 | 0.0 | 0.0 |
| 8412 | Dips, gravies, other sauces | 0.0 | 0.0 | 0.0 |
| 8802 | Sugars & honey | 0.0 | 0.0 | 0.0 |
| 8804 | Sugar substitutes | 0.0 | 0.0 | 0.0 |
| 8806 | Jams, syrups, toppings | 0.0 | 0.0 | 0.0 |
| **9002** | **Baby food: cereals** | **6.7** | **13.8** | **1.5** |
| 9004 | Baby food: fruit | 0.1 | 0.5 | 0.0 |
| **9006** | **Baby food: vegetable** | **0.3** | **1.6** | **0.1** |
| **9008** | **Baby food: meat & dinners** | **0.1** | **1.2** | **0.4** |
| 9010 | Baby food: yogurt | 0.0 | 0.5 | 0.2 |
| 9012 | Baby food: snacks & sweets | 0.0 | 0.7 | 0.5 |
| 9202 | Baby juice | 0.1 | 0.8 | 0.2 |
| 9204 | Baby water | 0.1 | 0.1 | 0.0 |
| **9402** | **Formula, ready-to-feed** | **2.6** | **2.9** | **0.6** |
| **9404** | **Formula, prepared from powder** | **65.9** | **46.8** | **1.8** |
| **9406** | **Formula, prepared from concentrate** | **5.8** | **3.7** | **0.1** |
| **9602** | **Human milk** | **18.0** | **5.9** | **0.7** |
| 9999 | Not included in a food category | 0.0 | 0.0 | 0.1 |
|  | Total | 100 | 100 | 100 |

^a^ Contributions from all minor WWEIA food categories are reported. Those in bold contribute ≥1% to daily intake in at least one age group.

**Table S15.** Food sources of magnesium among U.S. infants and toddlers aged 0–23.9 months by age group: NHANES 2005-12 (*n* = 2740) ^a^.

| **WWEIA Minor Food category code** | **Food category** | **% of daily intake** | | |
| --- | --- | --- | --- | --- |
|  |  | **0–5.9 mo (*n* = 765)** | **6–11.9 mo (*n* = 854)** | **12–23.9 mo  (*n* = 1121)** |
| **1002** | **Milk, whole** | **0.0** | **3.3** | **21.0** |
| **1004** | **Milk, reduced fat** | **0.0** | **0.7** | **5.8** |
| 1006 | Milk, low-fat | 0.0 | 0.4 | 0.6 |
| 1008 | Milk, non-fat | 0.0 | 0.0 | 0.6 |
| 1202 | Flavored milk, whole | 0.0 | 0.0 | 0.9 |
| 1204 | Flavored milk, reduced fat | 0.0 | 0.0 | 0.4 |
| 1206 | Flavored milk, low-fat | 0.0 | 0.0 | 0.2 |
| 1208 | Flavored milk, non-fat | 0.0 | 0.0 | 0.0 |
| 1402 | Milk shakes & other dairy drinks | 0.0 | 0.0 | 0.0 |
| **1404** | **Milk substitutes** | **0.0** | **0.4** | **2.1** |
| **1602** | **Cheese** | **0.0** | **0.5** | **1.5** |
| 1604 | Cottage/ricotta cheese | 0.0 | 0.0 | 0.0 |
| 1802 | Yogurt, whole & reduced fat | 0.0 | 0.6 | 0.7 |
| **1804** | **Yogurt, low-fat & non-fat** | **0.0** | **0.5** | **1.1** |
| 2002 | Beef, excludes ground | 0.0 | 0.1 | 0.2 |
| 2004 | Ground beef | 0.0 | 0.1 | 0.2 |
| 2006 | Pork | 0.0 | 0.0 | 0.1 |
| 2008 | Lamb, goat, game | 0.0 | 0.0 | 0.0 |
| 2010 | Liver & organ meats | 0.0 | 0.0 | 0.0 |
| **2202** | **Chicken, whole pieces** | **0.0** | **0.3** | **1.3** |
| **2204** | **Chicken patties, nuggets & tenders** | **0.0** | **0.1** | **1.2** |
| 2206 | Turkey, duck, other poultry | 0.0 | 0.1 | 0.1 |
| 2402 | Fish | 0.0 | 0.1 | 0.2 |
| 2404 | Shellfish | 0.0 | 0.0 | 0.0 |
| **2502** | **Eggs & omelets** | **0.0** | **0.4** | **1.2** |
| 2602 | Cold cuts & cured meats | 0.0 | 0.1 | 0.4 |
| 2604 | Bacon | 0.0 | 0.0 | 0.1 |
| 2606 | Frankfurters | 0.0 | 0.1 | 0.4 |
| 2608 | Sausages | 0.0 | 0.1 | 0.2 |
| **2802** | **Beans, peas, legumes** | **0.0** | **1.3** | **1.4** |
| **2804** | **Nuts & seeds** | **0.0** | **0.2** | **1.6** |
| 2806 | Processed soy products | 0.0 | 0.1 | 0.2 |
| 3002 | Meat mixed dishes | 0.0 | 0.4 | 0.9 |
| 3004 | Poultry mixed dishes | 0.0 | 0.2 | 0.8 |
| 3006 | Seafood mixed dishes | 0.0 | 0.0 | 0.2 |
| 3202 | Rice mixed dishes | 0.0 | 0.3 | 0.6 |
| **3204** | **Pasta mixed dishes, excludes macaroni & cheese** | **0.0** | **1.2** | **2.5** |
| **3206** | **Macaroni & cheese** | **0.0** | **0.7** | **1.7** |
| 3208 | Turnovers & other grain-based items | 0.0 | 0.0 | 0.1 |
| 3402 | Fried rice & lo/chow mein | 0.0 | 0.0 | 0.2 |
| 3404 | Stir-fry & soy-based sauce mixtures | 0.0 | 0.1 | 0.1 |
| 3406 | Egg rolls, dumplings, sushi | 0.0 | 0.0 | 0.0 |
| 3502 | Burritos & tacos | 0.0 | 0.0 | 0.2 |
| 3504 | Nachos | 0.0 | 0.0 | 0.0 |
| 3506 | Other Mexican mixed dishes | 0.0 | 0.0 | 0.3 |
| 3602 | Pizza | 0.0 | 0.1 | 0.9 |
| 3702 | Burgers (single code) | 0.0 | 0.1 | 0.2 |
| 3704 | Chicken/turkey sandwiches (single code) | 0.0 | 0.0 | 0.1 |
| 3706 | Egg/breakfast sandwiches (single code) | 0.0 | 0.0 | 0.0 |
| 3708 | Other sandwiches (single code) | 0.0 | 0.0 | 0.2 |
| **3802** | **Soups** | **0.0** | **1.2** | **1.5** |
| 4002 | Rice | 0.0 | 0.5 | 0.5 |
| 4004 | Pasta, noodles, cooked grains | 0.0 | 0.0 | 0.2 |
| **4202** | **Yeast breads** | **0.0** | **0.8** | **2.6** |
| 4204 | Rolls & buns | 0.0 | 0.1 | 0.3 |
| 4206 | Bagels & English muffins | 0.0 | 0.0 | 0.2 |
| 4208 | Tortillas | 0.0 | 0.3 | 0.7 |
| 4402 | Biscuits, muffins, quick breads | 0.0 | 0.1 | 0.3 |
| 4404 | Pancakes, waffles, French toast | 0.0 | 0.1 | 0.5 |
| 4602 | Ready-to-eat cereal, higher sugar (>21.2g/100g) | 0.0 | 0.3 | 1.2 |
| **4604** | **Ready-to-eat cereal, lower sugar (≤21.2g/100g)** | **0.0** | **1.2** | **2.0** |
| **4802** | **Oatmeal** | **0.0** | **0.6** | **2.2** |
| 4804 | Grits & other cooked cereals | 0.0 | 0.2 | 0.3 |
| 5002 | Potato chips | 0.0 | 0.0 | 0.5 |
| **5004** | **Tortilla, corn, other chips** | **0.0** | **0.2** | **1.0** |
| 5006 | Popcorn | 0.0 | 0.0 | 0.1 |
| 5008 | Pretzels/snack mix | 0.0 | 0.0 | 0.3 |
| **5202** | **Crackers, excludes saltines** | **0.0** | **0.3** | **1.0** |
| 5204 | Saltine crackers | 0.0 | 0.1 | 0.1 |
| 5402 | Cereal bars | 0.0 | 0.0 | 0.6 |
| 5404 | Nutrition bars | 0.0 | 0.0 | 0.0 |
| 5502 | Cakes & pies | 0.0 | 0.1 | 0.2 |
| **5504** | **Cookies & brownies** | **0.0** | **0.7** | **1.4** |
| 5506 | Doughnuts, sweet rolls, pastries | 0.0 | 0.1 | 0.3 |
| 5702 | Candy containing chocolate | 0.0 | 0.0 | 0.2 |
| 5704 | Candy not containing chocolate | 0.0 | 0.0 | 0.2 |
| 5802 | Ice cream & frozen dairy desserts | 0.0 | 0.1 | 0.5 |
| 5804 | Pudding | 0.0 | 0.1 | 0.1 |
| 5806 | Gelatins, ices, sorbets | 0.0 | 0.0 | 0.1 |
| 6002 | Apples | 0.0 | 0.3 | 0.5 |
| **6004** | **Bananas** | **0.1** | **2.6** | **3.5** |
| 6006 | Grapes | 0.0 | 0.0 | 0.3 |
| 6008 | Peaches & nectarines | 0.0 | 0.1 | 0.2 |
| 6010 | Berries | 0.0 | 0.1 | 0.4 |
| 6012 | Citrus fruits | 0.0 | 0.1 | 0.5 |
| 6014 | Melons | 0.0 | 0.1 | 0.4 |
| 6016 | Dried fruits | 0.0 | 0.1 | 0.3 |
| 6018 | Other fruits & fruit salads | 0.0 | 0.2 | 0.6 |
| 6402 | Tomatoes | 0.0 | 0.0 | 0.2 |
| 6404 | Carrots | 0.0 | 0.1 | 0.1 |
| 6406 | Other red & orange vegetables | 0.0 | 0.3 | 0.2 |
| 6408 | Dark green vegetables, excludes lettuce | 0.0 | 0.1 | 0.4 |
| 6410 | Lettuce & lettuce salads | 0.0 | 0.0 | 0.0 |
| 6412 | String beans | 0.0 | 0.1 | 0.4 |
| 6414 | Onions | 0.0 | 0.0 | 0.0 |
| 6416 | Corn | 0.0 | 0.1 | 0.4 |
| 6418 | Other starchy vegetables | 0.0 | 0.5 | 0.3 |
| 6420 | Other vegetables & combinations | 0.0 | 0.5 | 0.5 |
| 6422 | Vegetable mixed dishes | 0.0 | 0.0 | 0.2 |
| 6802 | White potatoes, baked or boiled | 0.0 | 0.1 | 0.2 |
| **6804** | **French fries & other fried white potatoes** | **0.0** | **0.2** | **1.0** |
| 6806 | Mashed potatoes & white potato mixtures | 0.1 | 0.5 | 0.6 |
| **7002** | **Citrus juice** | **0.0** | **0.2** | **1.3** |
| **7004** | **Apple juice** | **0.1** | **0.5** | **1.8** |
| **7006** | **Other fruit juice** | **0.1** | **0.9** | **2.6** |
| 7008 | Vegetable juice | 0.0 | 0.0 | 0.1 |
| 7102 | Diet soft drinks | 0.0 | 0.0 | 0.0 |
| 7106 | Other diet drinks | 0.0 | 0.0 | 0.0 |
| 7202 | Soft drinks | 0.0 | 0.0 | 0.0 |
| **7204** | **Fruit drinks** | **0.0** | **0.2** | **1.7** |
| 7206 | Sport & energy drinks | 0.0 | 0.0 | 0.1 |
| 7208 | Nutritional beverages | 0.0 | 0.0 | 0.5 |
| 7302 | Coffee | 0.0 | 0.0 | 0.0 |
| 7304 | Tea | 0.0 | 0.0 | 0.2 |
| 7702 | Tap water | 0.1 | 0.5 | 0.7 |
| 7704 | Bottled water | 0.2 | 0.4 | 0.9 |
| 7802 | Flavored or carbonated water | 0.0 | 0.0 | 0.0 |
| 7804 | Enhanced or fortified water | 0.0 | 0.0 | 0.0 |
| 8002 | Butter & animal fats | 0.0 | 0.0 | 0.0 |
| 8004 | Margarine | 0.0 | 0.0 | 0.0 |
| 8006 | Cream cheese, sour cream, whipped cream | 0.0 | 0.0 | 0.0 |
| 8008 | Cream & cream substitutes | 0.0 | 0.0 | 0.0 |
| 8010 | Mayonnaise | 0.0 | 0.0 | 0.0 |
| 8012 | Salad dressings & vegetable oils | 0.0 | 0.0 | 0.0 |
| 8402 | Tomato-based condiments | 0.0 | 0.0 | 0.1 |
| 8404 | Soy-based condiments | 0.0 | 0.0 | 0.0 |
| 8406 | Mustard & other condiments | 0.0 | 0.0 | 0.0 |
| 8408 | Olives, pickles, pickled vegetables | 0.0 | 0.0 | 0.0 |
| 8410 | Pasta sauces, tomato-based | 0.0 | 0.0 | 0.1 |
| 8412 | Dips, gravies, other sauces | 0.0 | 0.0 | 0.0 |
| 8802 | Sugars & honey | 0.0 | 0.0 | 0.0 |
| 8804 | Sugar substitutes | 0.0 | 0.0 | 0.0 |
| 8806 | Jams, syrups, toppings | 0.0 | 0.0 | 0.1 |
| **9002** | **Baby food: cereals** | **13.5** | **18.4** | **1.8** |
| **9004** | **Baby food: fruit** | **1.0** | **5.2** | **0.4** |
| **9006** | **Baby food: vegetable** | **1.9** | **6.1** | **0.4** |
| **9008** | **Baby food: meat & dinners** | **0.2** | **3.1** | **0.8** |
| 9010 | Baby food: yogurt | 0.0 | 0.4 | 0.1 |
| 9012 | Baby food: snacks & sweets | 0.0 | 0.7 | 0.2 |
| **9202** | **Baby juice** | **0.4** | **1.3** | **0.3** |
| 9204 | Baby water | 0.1 | 0.1 | 0.0 |
| **9402** | **Formula, ready-to-feed** | **2.0** | **1.5** | **0.5** |
| **9404** | **Formula, prepared from powder** | **59.6** | **28.4** | **0.9** |
| **9406** | **Formula, prepared from concentrate** | **5.3** | **2.4** | **0.0** |
| **9602** | **Human milk** | **15.0** | **3.3** | **0.4** |
| 9999 | Not included in a food category | 0.0 | 0.0 | 0.4 |
|  | Total | 100 | 100 | 100 |

^a^ Contributions from all minor WWEIA food categories are reported. Those in bold contribute ≥1% to daily intake in at least one age group.

**Table S16.** Food sources of iron among U.S. infants and toddlers aged 0–23.9 months by age group: NHANES 2005-12 (*n* = 2740) ^a^.

| **WWEIA Minor Food category code** | **Food category** | **% of daily intake** | | |
| --- | --- | --- | --- | --- |
|  |  | **0–5.9 mo (*n* = 765)** | **6–11.9 mo (*n* = 854)** | **12–23.9 mo  (*n* = 1121)** |
| **1002** | **Milk, whole** | **0.0** | **0.1** | **1.2** |
| 1004 | Milk, reduced fat | 0.0 | 0.0 | 0.2 |
| 1006 | Milk, low-fat | 0.0 | 0.0 | 0.0 |
| 1008 | Milk, non-fat | 0.0 | 0.0 | 0.0 |
| 1202 | Flavored milk, whole | 0.0 | 0.0 | 0.2 |
| 1204 | Flavored milk, reduced fat | 0.0 | 0.0 | 0.2 |
| 1206 | Flavored milk, low-fat | 0.0 | 0.0 | 0.1 |
| 1208 | Flavored milk, non-fat | 0.0 | 0.0 | 0.0 |
| 1402 | Milk shakes & other dairy drinks | 0.0 | 0.0 | 0.0 |
| 1404 | Milk substitutes | 0.0 | 0.1 | 0.9 |
| 1602 | Cheese | 0.0 | 0.1 | 0.5 |
| 1604 | Cottage/ricotta cheese | 0.0 | 0.0 | 0.0 |
| 1802 | Yogurt, whole & reduced fat | 0.0 | 0.0 | 0.1 |
| 1804 | Yogurt, low-fat & non-fat | 0.0 | 0.0 | 0.1 |
| 2002 | Beef, excludes ground | 0.0 | 0.0 | 0.4 |
| 2004 | Ground beef | 0.0 | 0.1 | 0.5 |
| 2006 | Pork | 0.0 | 0.0 | 0.1 |
| 2008 | Lamb, goat, game | 0.0 | 0.0 | 0.0 |
| 2010 | Liver & organ meats | 0.0 | 0.0 | 0.1 |
| 2202 | Chicken, whole pieces | 0.0 | 0.1 | 0.9 |
| 2204 | Chicken patties, nuggets & tenders | 0.0 | 0.0 | 0.8 |
| 2206 | Turkey, duck, other poultry | 0.0 | 0.0 | 0.1 |
| 2402 | Fish | 0.0 | 0.0 | 0.1 |
| 2404 | Shellfish | 0.0 | 0.0 | 0.0 |
| **2502** | **Eggs & omelets** | **0.0** | **0.3** | **2.3** |
| 2602 | Cold cuts & cured meats | 0.0 | 0.1 | 0.4 |
| 2604 | Bacon | 0.0 | 0.0 | 0.1 |
| 2606 | Frankfurters | 0.0 | 0.1 | 0.8 |
| 2608 | Sausages | 0.0 | 0.0 | 0.3 |
| **2802** | **Beans, peas, legumes** | **0.0** | **0.7** | **1.2** |
| 2804 | Nuts & seeds | 0.0 | 0.0 | 0.5 |
| 2806 | Processed soy products | 0.0 | 0.0 | 0.2 |
| **3002** | **Meat mixed dishes** | **0.0** | **0.2** | **1.2** |
| 3004 | Poultry mixed dishes | 0.0 | 0.1 | 0.9 |
| 3006 | Seafood mixed dishes | 0.0 | 0.0 | 0.2 |
| 3202 | Rice mixed dishes | 0.0 | 0.2 | 0.8 |
| **3204** | **Pasta mixed dishes, excludes macaroni & cheese** | **0.0** | **0.5** | **3.1** |
| **3206** | **Macaroni & cheese** | **0.0** | **0.3** | **1.6** |
| 3208 | Turnovers & other grain-based items | 0.0 | 0.0 | 0.1 |
| 3402 | Fried rice & lo/chow mein | 0.0 | 0.0 | 0.2 |
| 3404 | Stir-fry & soy-based sauce mixtures | 0.0 | 0.0 | 0.1 |
| 3406 | Egg rolls, dumplings, sushi | 0.0 | 0.0 | 0.0 |
| 3502 | Burritos & tacos | 0.0 | 0.0 | 0.3 |
| 3504 | Nachos | 0.0 | 0.0 | 0.0 |
| 3506 | Other Mexican mixed dishes | 0.0 | 0.0 | 0.4 |
| **3602** | **Pizza** | **0.0** | **0.0** | **1.4** |
| 3702 | Burgers (single code) | 0.0 | 0.1 | 0.3 |
| 3704 | Chicken/turkey sandwiches (single code) | 0.0 | 0.0 | 0.1 |
| 3706 | Egg/breakfast sandwiches (single code) | 0.0 | 0.0 | 0.0 |
| 3708 | Other sandwiches (single code) | 0.0 | 0.0 | 0.4 |
| **3802** | **Soups** | **0.0** | **0.4** | **2.1** |
| 4002 | Rice | 0.0 | 0.2 | 0.7 |
| 4004 | Pasta, noodles, cooked grains | 0.0 | 0.0 | 0.3 |
| **4202** | **Yeast breads** | **0.0** | **0.5** | **4.0** |
| 4204 | Rolls & buns | 0.0 | 0.1 | 0.9 |
| 4206 | Bagels & English muffins | 0.0 | 0.0 | 0.5 |
| 4208 | Tortillas | 0.0 | 0.1 | 0.5 |
| 4402 | Biscuits, muffins, quick breads | 0.0 | 0.1 | 0.7 |
| **4404** | **Pancakes, waffles, French toast** | **0.0** | **0.1** | **1.6** |
| **4602** | **Ready-to-eat cereal, higher sugar (>21.2g/100g)** | **0.0** | **0.4** | **6.4** |
| **4604** | **Ready-to-eat cereal, lower sugar (≤21.2g/100g)** | **0.0** | **2.6** | **12.4** |
| **4802** | **Oatmeal** | **0.0** | **0.4** | **3.8** |
| **4804** | **Grits & other cooked cereals** | **0.0** | **0.4** | **1.7** |
| 5002 | Potato chips | 0.0 | 0.0 | 0.2 |
| 5004 | Tortilla, corn, other chips | 0.0 | 0.1 | 0.6 |
| 5006 | Popcorn | 0.0 | 0.0 | 0.1 |
| 5008 | Pretzels/snack mix | 0.0 | 0.1 | 0.9 |
| **5202** | **Crackers, excludes saltines** | **0.0** | **0.2** | **2.3** |
| 5204 | Saltine crackers | 0.0 | 0.1 | 0.5 |
| 5402 | Cereal bars | 0.0 | 0.0 | 0.8 |
| 5404 | Nutrition bars | 0.0 | 0.0 | 0.0 |
| 5502 | Cakes & pies | 0.0 | 0.0 | 0.5 |
| **5504** | **Cookies & brownies** | **0.0** | **0.6** | **3.1** |
| 5506 | Doughnuts, sweet rolls, pastries | 0.0 | 0.1 | 0.9 |
| 5702 | Candy containing chocolate | 0.0 | 0.0 | 0.1 |
| 5704 | Candy not containing chocolate | 0.0 | 0.0 | 0.2 |
| 5802 | Ice cream & frozen dairy desserts | 0.0 | 0.0 | 0.1 |
| 5804 | Pudding | 0.0 | 0.0 | 0.1 |
| 5806 | Gelatins, ices, sorbets | 0.0 | 0.0 | 0.1 |
| 6002 | Apples | 0.0 | 0.1 | 0.3 |
| 6004 | Bananas | 0.0 | 0.2 | 0.6 |
| 6006 | Grapes | 0.0 | 0.0 | 0.2 |
| 6008 | Peaches & nectarines | 0.0 | 0.0 | 0.2 |
| 6010 | Berries | 0.0 | 0.0 | 0.2 |
| 6012 | Citrus fruits | 0.0 | 0.0 | 0.1 |
| 6014 | Melons | 0.0 | 0.0 | 0.1 |
| 6016 | Dried fruits | 0.0 | 0.0 | 0.3 |
| 6018 | Other fruits & fruit salads | 0.0 | 0.0 | 0.3 |
| 6402 | Tomatoes | 0.0 | 0.0 | 0.1 |
| 6404 | Carrots | 0.0 | 0.0 | 0.1 |
| 6406 | Other red & orange vegetables | 0.0 | 0.1 | 0.1 |
| 6408 | Dark green vegetables, excludes lettuce | 0.0 | 0.0 | 0.3 |
| 6410 | Lettuce & lettuce salads | 0.0 | 0.0 | 0.0 |
| 6412 | String beans | 0.0 | 0.1 | 0.5 |
| 6414 | Onions | 0.0 | 0.0 | 0.0 |
| 6416 | Corn | 0.0 | 0.0 | 0.2 |
| 6418 | Other starchy vegetables | 0.0 | 0.1 | 0.2 |
| 6420 | Other vegetables & combinations | 0.0 | 0.1 | 0.3 |
| 6422 | Vegetable mixed dishes | 0.0 | 0.0 | 0.2 |
| 6802 | White potatoes, baked or boiled | 0.0 | 0.0 | 0.1 |
| 6804 | French fries & other fried white potatoes | 0.0 | 0.1 | 0.7 |
| 6806 | Mashed potatoes & white potato mixtures | 0.0 | 0.1 | 0.2 |
| 7002 | Citrus juice | 0.0 | 0.0 | 0.4 |
| **7004** | **Apple juice** | **0.0** | **0.2** | **1.5** |
| **7006** | **Other fruit juice** | **0.0** | **0.2** | **1.2** |
| 7008 | Vegetable juice | 0.0 | 0.0 | 0.0 |
| 7102 | Diet soft drinks | 0.0 | 0.0 | 0.0 |
| 7106 | Other diet drinks | 0.0 | 0.0 | 0.0 |
| 7202 | Soft drinks | 0.0 | 0.0 | 0.1 |
| 7204 | Fruit drinks | 0.0 | 0.0 | 0.7 |
| 7206 | Sport & energy drinks | 0.0 | 0.0 | 0.0 |
| 7208 | Nutritional beverages | 0.0 | 0.0 | 0.4 |
| 7302 | Coffee | 0.0 | 0.0 | 0.0 |
| 7304 | Tea | 0.0 | 0.0 | 0.1 |
| 7702 | Tap water | 0.0 | 0.0 | 0.0 |
| 7704 | Bottled water | 0.0 | 0.0 | 0.0 |
| 7802 | Flavored or carbonated water | 0.0 | 0.0 | 0.0 |
| 7804 | Enhanced or fortified water | 0.0 | 0.0 | 0.0 |
| 8002 | Butter & animal fats | 0.0 | 0.0 | 0.0 |
| 8004 | Margarine | 0.0 | 0.0 | 0.0 |
| 8006 | Cream cheese, sour cream, whipped cream | 0.0 | 0.0 | 0.0 |
| 8008 | Cream & cream substitutes | 0.0 | 0.0 | 0.0 |
| 8010 | Mayonnaise | 0.0 | 0.0 | 0.0 |
| 8012 | Salad dressings & vegetable oils | 0.0 | 0.0 | 0.0 |
| 8402 | Tomato-based condiments | 0.0 | 0.0 | 0.0 |
| 8404 | Soy-based condiments | 0.0 | 0.0 | 0.0 |
| 8406 | Mustard & other condiments | 0.0 | 0.0 | 0.0 |
| 8408 | Olives, pickles, pickled vegetables | 0.0 | 0.0 | 0.1 |
| 8410 | Pasta sauces, tomato-based | 0.0 | 0.0 | 0.1 |
| 8412 | Dips, gravies, other sauces | 0.0 | 0.0 | 0.1 |
| 8802 | Sugars & honey | 0.0 | 0.0 | 0.0 |
| 8804 | Sugar substitutes | 0.0 | 0.0 | 0.0 |
| 8806 | Jams, syrups, toppings | 0.0 | 0.0 | 0.1 |
| **9002** | **Baby food: cereals** | **20.8** | **38.6** | **11.9** |
| 9004 | Baby food: fruit | 0.1 | 0.9 | 0.2 |
| **9006** | **Baby food: vegetable** | **0.4** | **1.4** | **0.2** |
| **9008** | **Baby food: meat & dinners** | **0.1** | **1.0** | **0.7** |
| 9010 | Baby food: yogurt | 0.0 | 0.1 | 0.0 |
| **9012** | **Baby food: snacks & sweets** | **0.0** | **1.1** | **1.5** |
| 9202 | Baby juice | 0.2 | 0.6 | 0.5 |
| 9204 | Baby water | 0.0 | 0.0 | 0.0 |
| **9402** | **Formula, ready-to-feed** | **2.7** | **2.5** | **1.2** |
| **9404** | **Formula, prepared from powder** | **68.5** | **39.1** | **3.3** |
| **9406** | **Formula, prepared from concentrate** | **6.2** | **3.2** | **0.1** |
| 9602 | Human milk | 0.9 | 0.2 | 0.1 |
| 9999 | Not included in a food category | 0.0 | 0.0 | 0.4 |
|  | Total | 100 | 100 | 100 |

^a^ Contributions from all minor WWEIA food categories are reported. Those in bold contribute ≥1% to daily intake in at least one age group.

**Table S17.** Food sources of zinc among U.S. infants and toddlers aged 0–23.9 months by age group: NHANES 2005-12 (*n* = 2740) ^a^.

| **WWEIA Minor Food category code** | **Food category** | **% of daily intake** | | |
| --- | --- | --- | --- | --- |
|  |  | **0-5.9 mo (*n* = 765)** | **6-11.9 mo (*n* = 854)** | **12-23.9 mo  (*n* = 1121)** |
| **1002** | **Milk, whole** | **0.0** | **2.2** | **20.0** |
| **1004** | **Milk, reduced fat** | **0.0** | **0.5** | **6.2** |
| 1006 | Milk, low-fat | 0.0 | 0.3 | 0.5 |
| 1008 | Milk, non-fat | 0.0 | 0.0 | 0.6 |
| 1202 | Flavored milk, whole | 0.0 | 0.0 | 0.8 |
| 1204 | Flavored milk, reduced fat | 0.0 | 0.0 | 0.4 |
| 1206 | Flavored milk, low-fat | 0.0 | 0.0 | 0.3 |
| 1208 | Flavored milk, non-fat | 0.0 | 0.0 | 0.0 |
| 1402 | Milk shakes & other dairy drinks | 0.0 | 0.0 | 0.0 |
| 1404 | Milk substitutes | 0.0 | 0.3 | 0.8 |
| **1602** | **Cheese** | **0.0** | **0.9** | **4.0** |
| 1604 | Cottage/ricotta cheese | 0.0 | 0.0 | 0.1 |
| 1802 | Yogurt, whole & reduced fat | 0.0 | 0.5 | 0.9 |
| **1804** | **Yogurt, low-fat & non-fat** | **0.0** | **0.5** | **1.3** |
| **2002** | **Beef, excludes ground** | **0.0** | **0.3** | **1.2** |
| **2004** | **Ground beef** | **0.0** | **0.5** | **1.5** |
| 2006 | Pork | 0.0 | 0.0 | 0.3 |
| 2008 | Lamb, goat, game | 0.0 | 0.0 | 0.0 |
| 2010 | Liver & organ meats | 0.0 | 0.0 | 0.1 |
| **2202** | **Chicken, whole pieces** | **0.0** | **0.3** | **2.0** |
| **2204** | **Chicken patties, nuggets & tenders** | **0.0** | **0.1** | **1.0** |
| 2206 | Turkey, duck, other poultry | 0.0 | 0.2 | 0.2 |
| 2402 | Fish | 0.0 | 0.0 | 0.1 |
| 2404 | Shellfish | 0.0 | 0.0 | 0.0 |
| **2502** | **Eggs & omelets** | **0.0** | **0.6** | **2.7** |
| 2602 | Cold cuts & cured meats | 0.0 | 0.3 | 0.9 |
| 2604 | Bacon | 0.0 | 0.0 | 0.2 |
| **2606** | **Frankfurters** | **0.0** | **0.3** | **1.6** |
| 2608 | Sausages | 0.0 | 0.3 | 0.8 |
| **2802** | **Beans, peas, legumes** | **0.0** | **1.8** | **1.1** |
| 2804 | Nuts & seeds | 0.0 | 0.0 | 0.9 |
| 2806 | Processed soy products | 0.0 | 0.0 | 0.1 |
| **3002** | **Meat mixed dishes** | **0.0** | **0.8** | **2.6** |
| **3004** | **Poultry mixed dishes** | **0.0** | **0.2** | **1.0** |
| 3006 | Seafood mixed dishes | 0.0 | 0.0 | 0.2 |
| 3202 | Rice mixed dishes | 0.0 | 0.2 | 0.4 |
| **3204** | **Pasta mixed dishes, excludes macaroni & cheese** | **0.0** | **0.9** | **2.7** |
| **3206** | **Macaroni & cheese** | **0.0** | **0.5** | **1.7** |
| 3208 | Turnovers & other grain-based items | 0.0 | 0.0 | 0.1 |
| 3402 | Fried rice & lo/chow mein | 0.0 | 0.0 | 0.2 |
| 3404 | Stir-fry & soy-based sauce mixtures | 0.0 | 0.1 | 0.1 |
| 3406 | Egg rolls, dumplings, sushi | 0.0 | 0.0 | 0.0 |
| 3502 | Burritos & tacos | 0.0 | 0.0 | 0.4 |
| 3504 | Nachos | 0.0 | 0.0 | 0.0 |
| 3506 | Other Mexican mixed dishes | 0.0 | 0.0 | 0.4 |
| **3602** | **Pizza** | **0.0** | **0.1** | **1.4** |
| 3702 | Burgers (single code) | 0.0 | 0.1 | 0.4 |
| 3704 | Chicken/turkey sandwiches (single code) | 0.0 | 0.0 | 0.1 |
| 3706 | Egg/breakfast sandwiches (single code) | 0.0 | 0.0 | 0.0 |
| 3708 | Other sandwiches (single code) | 0.0 | 0.0 | 0.4 |
| **3802** | **Soups** | **0.0** | **0.7** | **1.5** |
| 4002 | Rice | 0.0 | 0.2 | 0.4 |
| 4004 | Pasta, noodles, cooked grains | 0.0 | 0.0 | 0.2 |
| **4202** | **Yeast breads** | **0.0** | **0.4** | **1.7** |
| 4204 | Rolls & buns | 0.0 | 0.0 | 0.3 |
| 4206 | Bagels & English muffins | 0.0 | 0.0 | 0.2 |
| 4208 | Tortillas | 0.0 | 0.1 | 0.3 |
| 4402 | Biscuits, muffins, quick breads | 0.0 | 0.1 | 0.2 |
| 4404 | Pancakes, waffles, French toast | 0.0 | 0.1 | 0.4 |
| **4602** | **Ready-to-eat cereal, higher sugar (>21.2g/100g)** | **0.0** | **0.6** | **5.0** |
| **4604** | **Ready-to-eat cereal, lower sugar (≤21.2g/100g)** | **0.0** | **3.5** | **6.8** |
| **4802** | **Oatmeal** | **0.0** | **0.3** | **1.4** |
| 4804 | Grits & other cooked cereals | 0.0 | 0.1 | 0.2 |
| 5002 | Potato chips | 0.0 | 0.0 | 0.3 |
| 5004 | Tortilla, corn, other chips | 0.0 | 0.1 | 0.5 |
| 5006 | Popcorn | 0.0 | 0.0 | 0.1 |
| 5008 | Pretzels/snack mix | 0.0 | 0.0 | 0.3 |
| 5202 | Crackers, excludes saltines | 0.0 | 0.2 | 0.7 |
| 5204 | Saltine crackers | 0.0 | 0.0 | 0.1 |
| 5402 | Cereal bars | 0.0 | 0.0 | 0.6 |
| 5404 | Nutrition bars | 0.0 | 0.0 | 0.0 |
| 5502 | Cakes & pies | 0.0 | 0.0 | 0.2 |
| 5504 | Cookies & brownies | 0.0 | 0.3 | 0.8 |
| 5506 | Doughnuts, sweet rolls, pastries | 0.0 | 0.0 | 0.2 |
| 5702 | Candy containing chocolate | 0.0 | 0.0 | 0.2 |
| 5704 | Candy not containing chocolate | 0.0 | 0.0 | 0.1 |
| 5802 | Ice cream & frozen dairy desserts | 0.0 | 0.1 | 0.5 |
| 5804 | Pudding | 0.0 | 0.0 | 0.1 |
| 5806 | Gelatins, ices, sorbets | 0.0 | 0.0 | 0.1 |
| 6002 | Apples | 0.0 | 0.0 | 0.1 |
| 6004 | Bananas | 0.0 | 0.3 | 0.5 |
| 6006 | Grapes | 0.0 | 0.0 | 0.1 |
| 6008 | Peaches & nectarines | 0.0 | 0.0 | 0.1 |
| 6010 | Berries | 0.0 | 0.0 | 0.1 |
| 6012 | Citrus fruits | 0.0 | 0.0 | 0.3 |
| 6014 | Melons | 0.0 | 0.0 | 0.1 |
| 6016 | Dried fruits | 0.0 | 0.0 | 0.1 |
| 6018 | Other fruits & fruit salads | 0.0 | 0.0 | 0.2 |
| 6402 | Tomatoes | 0.0 | 0.0 | 0.1 |
| 6404 | Carrots | 0.0 | 0.0 | 0.1 |
| 6406 | Other red & orange vegetables | 0.0 | 0.1 | 0.1 |
| 6408 | Dark green vegetables, excludes lettuce | 0.0 | 0.0 | 0.2 |
| 6410 | Lettuce & lettuce salads | 0.0 | 0.0 | 0.0 |
| 6412 | String beans | 0.0 | 0.0 | 0.2 |
| 6414 | Onions | 0.0 | 0.0 | 0.0 |
| 6416 | Corn | 0.0 | 0.0 | 0.2 |
| 6418 | Other starchy vegetables | 0.0 | 0.2 | 0.2 |
| 6420 | Other vegetables & combinations | 0.0 | 0.2 | 0.3 |
| 6422 | Vegetable mixed dishes | 0.0 | 0.0 | 0.1 |
| 6802 | White potatoes, baked or boiled | 0.0 | 0.0 | 0.1 |
| 6804 | French fries & other fried white potatoes | 0.0 | 0.1 | 0.5 |
| 6806 | Mashed potatoes & white potato mixtures | 0.0 | 0.1 | 0.2 |
| 7002 | Citrus juice | 0.0 | 0.0 | 0.2 |
| 7004 | Apple juice | 0.0 | 0.0 | 0.2 |
| 7006 | Other fruit juice | 0.0 | 0.1 | 0.5 |
| 7008 | Vegetable juice | 0.0 | 0.0 | 0.0 |
| 7102 | Diet soft drinks | 0.0 | 0.0 | 0.0 |
| 7106 | Other diet drinks | 0.0 | 0.0 | 0.0 |
| 7202 | Soft drinks | 0.0 | 0.0 | 0.1 |
| 7204 | Fruit drinks | 0.0 | 0.0 | 0.3 |
| 7206 | Sport & energy drinks | 0.0 | 0.0 | 0.0 |
| 7208 | Nutritional beverages | 0.0 | 0.0 | 0.4 |
| 7302 | Coffee | 0.0 | 0.0 | 0.0 |
| 7304 | Tea | 0.0 | 0.0 | 0.0 |
| 7702 | Tap water | 0.0 | 0.1 | 0.2 |
| 7704 | Bottled water | 0.0 | 0.0 | 0.0 |
| 7802 | Flavored or carbonated water | 0.0 | 0.0 | 0.0 |
| 7804 | Enhanced or fortified water | 0.0 | 0.0 | 0.0 |
| 8002 | Butter & animal fats | 0.0 | 0.0 | 0.0 |
| 8004 | Margarine | 0.0 | 0.0 | 0.0 |
| 8006 | Cream cheese, sour cream, whipped cream | 0.0 | 0.0 | 0.0 |
| 8008 | Cream & cream substitutes | 0.0 | 0.0 | 0.0 |
| 8010 | Mayonnaise | 0.0 | 0.0 | 0.0 |
| 8012 | Salad dressings & vegetable oils | 0.0 | 0.0 | 0.0 |
| 8402 | Tomato-based condiments | 0.0 | 0.0 | 0.0 |
| 8404 | Soy-based condiments | 0.0 | 0.0 | 0.0 |
| 8406 | Mustard & other condiments | 0.0 | 0.0 | 0.0 |
| 8408 | Olives, pickles, pickled vegetables | 0.0 | 0.0 | 0.0 |
| 8410 | Pasta sauces, tomato-based | 0.0 | 0.0 | 0.1 |
| 8412 | Dips, gravies, other sauces | 0.0 | 0.0 | 0.1 |
| 8802 | Sugars & honey | 0.0 | 0.0 | 0.0 |
| 8804 | Sugar substitutes | 0.0 | 0.0 | 0.0 |
| 8806 | Jams, syrups, toppings | 0.0 | 0.0 | 0.1 |
| **9002** | **Baby food: cereals** | **2.7** | **9.8** | **1.7** |
| 9004 | Baby food: fruit | 0.1 | 0.6 | 0.1 |
| **9006** | **Baby food: vegetable** | **0.4** | **1.7** | **0.1** |
| **9008** | **Baby food: meat & dinners** | **0.2** | **2.9** | **0.9** |
| 9010 | Baby food: yogurt | 0.0 | 0.2 | 0.1 |
| 9012 | Baby food: snacks & sweets | 0.0 | 0.6 | 0.3 |
| 9202 | Baby juice | 0.0 | 0.2 | 0.1 |
| 9204 | Baby water | 0.0 | 0.0 | 0.0 |
| **9402** | **Formula, ready-to-feed** | **2.8** | **3.2** | **1.1** |
| **9404** | **Formula, prepared from powder** | **75.8** | **51.1** | **2.2** |
| **9406** | **Formula, prepared from concentrate** | **7.1** | **4.6** | **0.1** |
| **9602** | **Human milk** | **10.7** | **3.3** | **0.6** |
| 9999 | Not included in a food category | 0.0 | 0.0 | 0.3 |
|  | Total | 100 | 100 | 100 |

^a^ Contributions from all minor WWEIA food categories are reported. Those in bold contribute ≥1% to daily intake in at least one age group.
